# Rationally designed ruthenium complexes for 1- and 2-photon photodynamic therapy

**DOI:** 10.1038/s41467-020-16993-0

**Published:** 2020-06-26

**Authors:** Johannes Karges, Shi Kuang, Federica Maschietto, Olivier Blacque, Ilaria Ciofini, Hui Chao, Gilles Gasser

**Affiliations:** 1 Chimie ParisTech, PSL University, CNRS, Institute of Chemistry for Life and Health Sciences, Laboratory for Inorganic Chemical Biology, 75005 Paris, France; 2 MOE Key Laboratory of Bioinorganic and Synthetic Chemistry, School of Chemistry, Sun Yat-sen University, Guangzhou, 510275 P.R. China; 3 Chimie ParisTech, PSL University, CNRS, Institute of Chemistry for Life and Health Sciences, Theoretical Chemistry and Modelling, 75005 Paris, France; 4 Department of Chemistry, University of Zurich, Winterthurerstrasse 190, CH-8057 Zurich, Switzerland

**Keywords:** Cancer therapy, Targeted therapies, Bioinorganic chemistry

## Abstract

The use of photodynamic therapy (PDT) against cancer has received increasing attention over recent years. However, the application of the currently approved photosensitizers (PSs) is limited by their poor aqueous solubility, aggregation, photobleaching and slow clearance from the body. To overcome these limitations, there is a need for the development of new classes of PSs with ruthenium(II) polypyridine complexes currently gaining momentum. However, these compounds generally lack significant absorption in the biological spectral window, limiting their application to treat deep-seated or large tumors. To overcome this drawback, ruthenium(II) polypyridine complexes designed in silico with (*E*,*E*′)-4,4′-bisstyryl-2,2′-bipyridine ligands show impressive 1- and 2-Photon absorption up to a magnitude higher than the ones published so far. While nontoxic in the dark, these compounds are phototoxic in various 2D monolayer cells, 3D multicellular tumor spheroids and are able to eradicate a multiresistant tumor inside a mouse model upon clinically relevant 1-Photon and 2-Photon excitation.

## Abstract (web summary)

Photosensitizers that  are stable in biological conditions with absorption in the biological spectral window are needed for photodynamic therapy. Here, the authors report on the development of a Ruthenium complex for 1 and 2-photon therapy to address these issues and demonstrate application in vivo.

## Introduction

Due to the increasing impact of cancer on the life quality and mortality of humans, increasing research efforts are devoted toward the development of anticancer drugs and strategies. Among the most commonly used techniques to fight this disease (i.e., surgery, chemotherapy, and radiotherapy), photodynamic therapy (PDT) is receiving increasing attention during the last decades. In PDT, a photosensitizer (PS) is activated upon light irradiation to generate reactive oxygen species (ROS)^1,2^. As the majority  of currently approved PSs are based on tetrapyrrolic structures (i.e., porphyrin, chlorin, and phthalocyanine), these compounds share similar drawbacks (e.g., poor aqueous solubility, aggregation, photobleaching, slow clearance from the body, and hepatotoxicity)^3–5^. To overcome these limitations, there is a need for the development of new classes of PSs. Among others, the use of transition metal complexes^6–11^ and especially, Ru(II) polypyridine complexes are gaining momentum due to their attractive photophysical and chemical properties (i.e., strong luminescence, high singlet oxygen production, high chemical, and photophysical stability)^12–22^, with the compound TLD-1433 having just entered phase II clinical trials for the treatment of non-muscle invasive bladder cancer^23–25^. Despite these remarkable properties, the vast majority of Ru(II) polypyridine complexes are excited using either blue or UV-A light. As the light tissue penetration depth is rather poor at these wavelengths, the application of these compounds to treat deep-seated or large tumors is limited^26–30^. To circumvent this drawback, there is a need for the development of PSs with an absorption towards the biological spectral window (600–900 nm), which can be achieved by a red-shifted one-photon (1P) absorption or the use of a two-photon (2P) absorption process for 2P PDT, a technique that is not employed yet in the clinic. Worthy of note, the use of dihydrolipoic acid-coated gold nanoclusters was recently reported as an efficient 2P PDT agent^31^. However, the ability of Ru(II) polypyridine complexes to absorb 2P simultaneously, expressed as the 2P cross-section, remains relatively weak (~40–250 Goeppert-Mayer (GM)), limiting their applications in this field of research^32–38^.

To tackle these drawbacks, herein, we report the design of Ru(II) polypyridine complexes with a red-shifted 1P and exceptionally strong 2P absorption using an in silico optimization. The resulting compounds were synthesized, characterized, and photophysically and biologically evaluated in-depth. Strikingly, while being able to overcome the limitations of clinically applied PS, the compounds are phototoxic in various 2D monolayer cells, 3D multicellular tumor spheroids (MCTS) and able to eradicate a multiresistant tumor inside a mouse model upon clinically relevant 1P and 2P excitation.

## Results

### Rational design

With the aim of enhancing the absorption properties of Ru(2,2′-bipyridine)_3_ without deterioration of its 1P absorption properties, the bipyridine ligand was functionalized with rigid π conjugated substituents acting as electron-donating groups. This is expected to induce a 1P absorption red-shift toward the biological spectral window by intercalation of donor-centered orbitals in the Ru centered frontier orbitals manifold (allowing transitions of metal-to-ligand charge transfer (MLCT) and ligand-to-metal charge transfer (LMCT) character at low energy). As *trans*-stilbene are highly effective 2P dyes^39,40^, it would be of high interest to extend the ligand scaffold with such a moiety as well as to include terminal donor groups, namely (*E*,*E*′)-4,4′-bisstyryl-2,2′-bipyridine (L-H), (*E,E*′)-4,4′-bis[*p*-(*N*,*N*-dimethylamino)styryl]-2,2′-bipyridine (L-NMe_2_) and (*E,E*′)-4,4′-bis[*p*-methoxystyryl]-2,2′-bipyridine (L-OMe). The predicted UV–vis spectra for the resulting complexes of L-H and the L-OMe series (Supplementary Fig. 1) show the presence of two main bands in the 400–600 nm region, stemming from both MLCT/LMCT type transitions but also from ligand-centered (LC) charge-transfer excitations. Detailed analysis of the 1P and of the lowest lying 2P computed data (Supplementary Tables 1–4) indicated that the most intense lowest lying 2P absorption processes are associated with LCCT transitions with an average CT distance of 2.9–6.3 Å. Overall, the computed data suggests that Ru(II) coordinated (*E*,*E*′)-4,4′-bisstyryl-2,2′-bipyridine complexes with donor substituents, have a red-shifted 1P and a strong 2P absorption.

### Synthesis and characterization

Before the synthesis of the final compounds (Fig. 1), the experimental procedures to obtain the corresponding ligands were optimized^41–44^ to a one-step high-yielding synthesis using mild conditions (Supplementary Fig. 2). Having the ligands in hand, the desired complexes **1**–**7** were synthesized. All compounds were analyzed by ^1^H, ^13^C-NMR, ESI-HRMS (Supplementary Figs. 3–25) and their purity confirmed by HPLC as well as elemental analysis. In addition, the ligands L-H, L-NMe_2_, and L-OMe as well as **3** were characterized using single-crystal X-ray crystallography (Supplementary Tables 5–6, Supplementary Figs. 26–29).

**Fig. 1:**
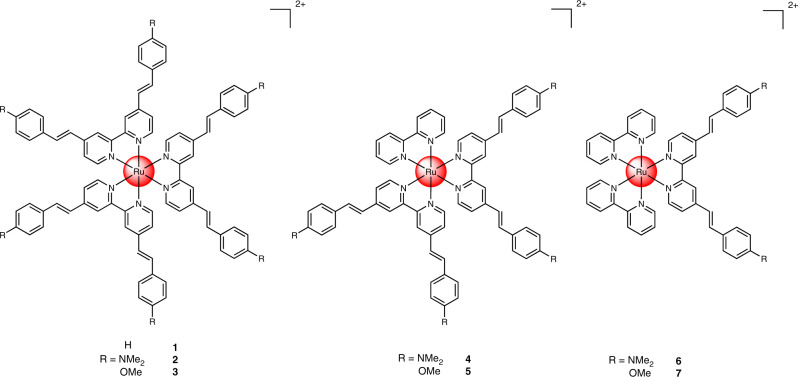
Chemical structures of complexes **1**–**7** investigated in this study. The complexes were isolated as hexafluorophosphate salts.

### Photophysical evaluation

**Fig. 2:**
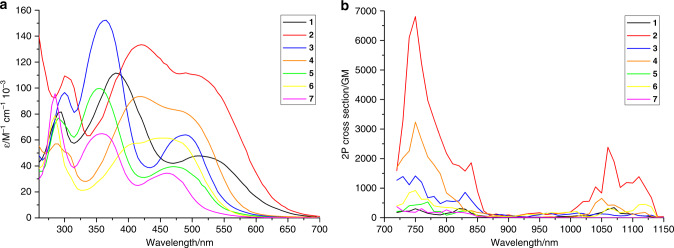
Absorption spectra of the complexes **1**–**7**. **a** 1P absorption spectrum in acetonitrile and **b** 2P absorption spectrum in dichloromethane.

The photophysical properties of the complexes (Table 1, Supplementary Fig. 30) were then experimentally investigated to evaluate their potential as PDT PSs. In agreement with theoretical findings, the compounds generally show a red-shift of the first 1P absorption (Fig. 2a) band maximum of about 50–70 nm for the symmetric Ru(II) complexes, in comparison to Ru(2,2′-bipyridine)_3_^45^ as well as an absorption tail toward the near-infrared region. Strikingly and in agreement with theoretical data, the reported compounds have an exceptionally strong 2P absorption (Fig. 2b) with values up to ~6800 GM, which is an order of magnitude higher than the ones reported before for other Ru(II) polypyridine complexes^32–38^. Interestingly, the L-NMe_2_ coordinated complexes were found with larger σ_2_ values than the L-OMe and L-H coordinated compounds. Overall, the compounds have a 1P absorption tail towards, and a strong 2P absorption, in the biological spectral window, potentially allowing for the treatment of deep-seated or large tumors.

**Table 1:** Spectroscopic properties and singlet oxygen quantum yields in acetonitrile and aqueous solution.

	Spectroscopic properties						Singlet oxygen quantum yield/%					
	*λ*_abs_/nm (*ε*/M^−1^ cm^−1^ × 10^−3^)	σ_2_/GM	*λ*_em_/nm	*Φ*_em_/%	*τ* air/ns	*τ* degassed/ns	Direct 450 nm CH_3_CN	Direct 450 nm D_2_O	Indirect 450 nm CH_3_CN	Indirect 450 nm PBS	Indirect 540 nm CH_3_CN	Indirect 540 nm PBS
**1**	300 (81.6), 385 (111.6), 515 (47.8)	147	677	1.9	86	385	65	n.d.	66	5	61	3
**2**	305 (109.5), 425 (133.4), 495 (111.8)	2175	709	>0.1	48	222	18	n.d.	25	3	16	1
**3**	305 (96.6), 370 (152.3), 495 (63.9)	573	682	1.1	76	338	52	n.d.	48	6	49	5
**4**	290 (57.0), 425 (93.5), 485 (82.6)	838	703	0.4	69	417	34	n.d.	40	2	37	3
**5**	295 (76.9), 360 (99.8), 475 (39.5)	313	674	1.4	36	231	54	n.d.	51	8	46	6
**6**	290 (79.0), 415 (57.3), 460 (61.4)	349	697	0.5	54	405	46	n.d.	53	2	38	2
**7**	290 (95.7), 365 (64.8), 465 (34.4)	263	664	2.8	96	542	75	n.d.	77	11	68	10

Average of three independent measurements.

*λ*_abs_ absorption maximum, *σ*_2_ 2P absorption cross section, *λ*_em_ emission maximum, *Φ*_em_ luminescence quantum yield, *τ* excited state lifetime, n.d. not detectable.

The luminescence quantum yields of the L-OMe coordinated complexes (**3**: 1.1%, **5**: 1.4%, **7**: 2.8%) were found to be significantly higher than those of the L-H coordinated (**1**: 1.9%) or L-NMe_2_ coordinated (**2**: >0.1%, **4**: 0.4%, **6**: 0.5%) compounds. All complexes were found to have excited state lifetimes in the nanosecond range (Supplementary Figs. 31–37) in degassed (222–542 ns) and aerated saturated (36–96 ns) solutions. As the lifetimes drastically decrease in the presence of air, it indicates that the excited state can interact with a component in the air. For identification of the type of ROS produced upon light exposure, electron spin resonance (ESR) spectroscopy was employed using the singlet oxygen (^1^O_2_) scavenger 2,2,6,6–tetramethylpiperidine and the ^•^OOH or ^•^OH radical scavenger 5,5-dimethyl-1-pyrroline *N*-oxide. While no signals for the formation of ^•^OOH or ^•^OH radicals were detected, the formation of ^1^O_2_ in acetonitrile and PBS was confirmed by observation of the characteristic ^1^O_2_-induced triplet signal in the ESR spectrum (Supplementary Figs. 38–44). The amount of generated ^1^O_2_ was quantitatively determined by two methods: directly by measurement of the phosphorescence of ^1^O_2_; indirectly by monitoring the change in absorbance of a ^1^O_2_ scavenger. The singlet oxygen quantum yields were found to be between 16–77% in acetonitrile and 1–11% in an aqueous solution (Table 1). The comparison between the values indicates that the L-OMe coordinated complexes (**3**, **5**, **7**) are able to produce ^1^O_2_ more efficiently than **1** and the L-NMe_2_ coordinated Ru(II) complexes (**2**, **4**, **6**). Overall, complex **7** was found with the highest singlet oxygen quantum yield (acetonitrile: 68–77%, aqueous solution: 10–11%).

### Stability

The stability of the compounds was investigated by incubation in human plasma at 37 °C for 48 h. The comparison of the HPLC chromatograms (Supplementary Figs. 45–51) showed no change before and after incubation in human plasma for all compounds, indicative of the stability of the complexes under biological conditions. Additionally, the stability upon irradiation was investigated (Supplementary Figs. 52–60) as the majority of current clinically employed PSs suffer from this drawback. Importantly, no significant differences in the absorption spectra for the L-OMe coordinated complexes (**3**, **5**, **7**) and **1** were observed over time, indicating their photostability. On the contrary, small changes in the absorption spectra of the L-NMe_2_ coordinated complexes (**2**, **4**, **6**), especially of **2**, were observed, suggesting that the complexes are slightly photobleaching. Worthy of note, under identical experimental conditions, the absorption spectra of the known PS Protoporphyrin IX (PpIX) was found to be drastically changed, indicating a significantly stronger photobleaching. The study of the effect of the irradiation on the molecular structure of **2** by NMR spectroscopy suggests the decomposition of the compound (Supplementary Fig. 61).

### Biological evaluation on 2D monolayer cells

All compounds were found to be lipophilic with high distribution coefficients between an organic octanol and an aqueous PBS phase (Supplementary Table 7). The time-dependent cellular uptake of the complexes was then investigated in human cervical carcinoma (HeLa) cells by determining the amount of Ru inside the cells by inductively coupled plasma mass spectrometry (ICP-MS) at each time point. The results show that the asymptotic maximum (Supplementary Figs. 62–68) of the uptake of the compounds was reached within 8 h. As expected, the compounds with a higher lipophilicity, with the exception of **2**, were found to have the highest uptake (Supplementary Fig. 69). The uptake mechanism of the complexes was then investigated by blocking various pathways by preincubation with a cationic transporter (tetraethylammonium chloride), metabolic (2-deoxy-*D*-glucose and oligomycin), and endocytotic inhibitors (ammonium chloride or chloroquine) as well as at reduced temperature (4 °C). Since all compounds were found with a similar profile (Supplementary Figs. 70–76), it suggests that these are internalized by the same mechanism, namely an energy-dependent endocytosis pathway. The localization of the complexes in HeLa cells was then investigated by confocal laser scanning microscopy. The distribution pattern of the luminescence of the compounds by 1P-(Supplementary Fig. 77) or 2P-(Supplementary Fig. 78) excitation was compared with the ones of commercial dyes for major cellular organelles (i.e., nucleus, mitochondria, lysosomes, golgi apparatus, and endoplasmic reticulum). As no significant congruency was detected, it suggests that the complexes do not majorly localize in these organelles. In addition, the cellular localization was also investigated by separately extracting the cellular organelles (i.e., cytoplasm, mitochondria, lysosome, and nucleus) and determining the amount of Ru inside each organelle by ICP-MS. The results (Supplementary Fig. 79) indicate that all complexes majorly localize in the cytoplasm with a small amount of unselective accumulation.

After an assessment of the uptake and the generation of ^1^O_2_ upon light exposure in a cuvette, the generation of ROS inside of HeLa cells upon 1P-(488 nm, Supplementary Fig. 80) or 2P- (800 nm, Supplementary Fig. 81) excitation was confirmed using the probe 2′, 7′ -dichlorofluorescein diacetate. To study their efficiency as PDT PSs, their cytotoxicity in the dark as well as upon irradiation at 480 nm (10 min, 5.2 mW cm^−2^, 3.1 J cm^−2^) or 540 nm (40 min, 4.0 mW cm^−2^, 9.5 J cm^−2^) toward noncancerous retinal pigment epithelium (RPE-1), HeLa, mouse colon carcinoma (CT-26), and human glioblastoma astrocytoma (U373) cells was investigated. Importantly, all complexes were found to be nontoxic in the dark (IC_50,dark_ > 100 μM) in all cell lines (Supplementary Tables 8–9). This is an important requirement for a PDT agent. As desired, all compounds were found to be phototoxic in the micromolar range (IC_50,480 nm_ = 0.7 ± 0.4 – 53.6 ± 3.2 μM, IC_50,540 nm_ = 0.9 ± 0.3 – 83.1 ± 6.7 μM) in the different cell lines employed in this study. As the lead compound of this study, complex **7** had an IC_50_ value in the nanomolar range in CT-26 cells (IC_50,dark_ > 100 μM, IC_50,480 nm_ = 0.7 ± 0.4 μM, IC_50,540 nm_ = 0.9 ± 0.3 μM) with a PI value > 143. Under identical experimental conditions, the anticancer drug cisplatin and the well-known PS PpIX display a magnitude lower (photo-)toxicity. The cell death mechanism of the complexes was then evaluated by measuring the cell viability upon preincubation with autophagy (3-methyladenine), apoptosis (Z-VAD-FMK), paraptosis (cycloheximide) and necrosis (necrostatin-1) inhibitors (Supplementary Fig. 82). While apoptosis was found to be the cell death mechanism for **1**–**5**, **6**–**7** triggered cell death by a combination of apoptosis and paraptosis pathways.

### Biological evaluation on 3D multicellular tumor spheroids

**Fig. 3:**
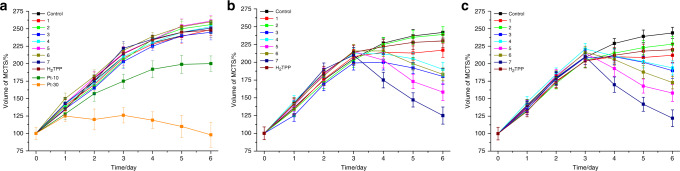
Tumor growth inhibition assay in HeLa MCTS. Change of the volume in MCTS in correlation to the time of the treatment. The MCTS were treated with compounds **1**–**7** (20 μM, 2% DMSO, *v*%), H_2_TPP (20 μM, 2% DMSO, *v*%), and cisplatin (10 μM and 30 μM). The MCTS were **a** strictly kept in the dark, **b** exposed to 1P irradiation (500 nm, 16.7 min, 10.0 mW cm^−2^, and 10 J cm^−2^), **c** exposed to 2P irradiation (800 nm, 10 J cm^−2^ with a section interval of 5 μm) on day 3. The error bars correspond to the standard deviation of the three replicates.

After evaluation of the biological effects of the compounds on 2D monolayer cells, their ability to act on 3D MCTS was investigated. MCTS simulate the conditions found in clinically treated tumors including hypoxia and proliferation gradients to the center^46^. Consequently, the penetration of the compounds inside of HeLa MCTS with a diameter of 800 μm was investigated by 1P and 2P z-stack confocal laser scanning microscopy. **4**–**7** completely penetrated the MCTS within 12 h with a strong luminescence signal at every section depth, whereas **1**–**3** were mostly found on the outer sphere (Supplementary Figs. 83–89). Upon an increased incubation time up to 60 h, the remaining compounds were also able to penetrate the MCTS, with the exception of **2**, which was still found mostly on the outer sphere (Supplementary Figs. 90–94). Following this, the tumor growth inhibition effect of **1**–**7** (20 μM) in HeLa MCTS was investigated and compared with the well-known PS tetraphenylporphyrin (H_2_TPP) (20 μM) and cisplatin (10 μM, 30 μM). The compounds were incubated for 3 days in the dark as this time was shown to be required for complete MCTS penetration. The MCTS were then exposed to 1P (500 nm, 16.7 min, 10.0 mW cm^−2^, and 10 J cm^−2^) or 2P irradiation (800 nm, 10 J cm^−2^, and section interval of 5 μm) on day 3. During the whole time period, the shape and volume of the MCTS was constantly monitored. As expected, the MCTS treated with **1**–**7** or H_2_TPP in the dark (Fig. 3a, representative image of MCTS: Supplementary Fig. 95) were asymptotically growing in the same manner than the control group, indicating that the compounds do not show any inhibitory effect, whereas cisplatin showed a weak effect on the tumor growth. On the contrary, the volume of the MCTS treated with complexes **1**–**7** and exposed to 1P or 2P irradiation (Fig. 3b, c, representative image of MCTS: Supplementary Figs. 96–97) significantly shrank, demonstrating their strong tumor inhibition effect.

**Fig. 4:**
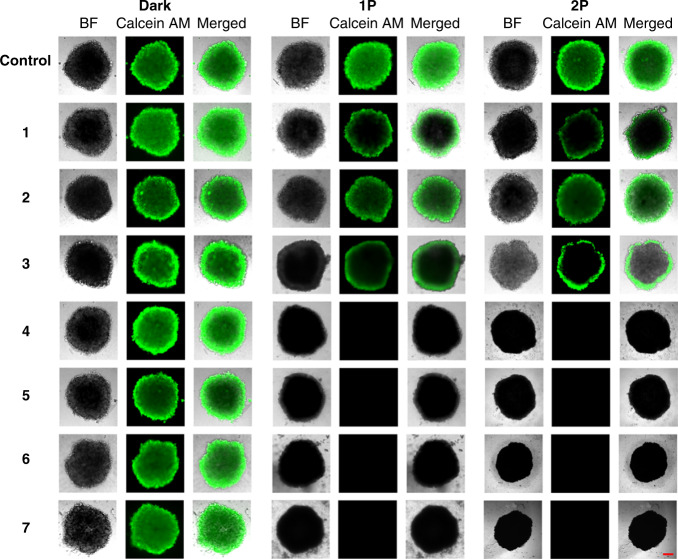
Representative image of the cell viability assay in HeLa MCTS. MCTS were treated with compounds **1**–**7** (20 μM, 2% DMSO, *v*%) in the dark for 3 days. After this time, MCTS were kept in the dark, exposed to 1P irradiation (500 nm, 16.7 min, 10.0 mW cm^−2^, and 10 J cm^−2^) or 2P irradiation (800 nm, 10 J cm^−2^, and section interval of 5 μm). After 2 days, the cell viability was assessed by measurement of the fluorescence of calcein (*λ*_ex_ = 495 nm, *λ*_em_ = 515 nm), which is generated in living cells from Calcein AM. The scale bar represents a length of 200 µm.

To further study the effect the complexes have on the tumor survival, the treated MCTS were stained with a cellular living cell kit using Calcein AM (Fig. 4). The fluorescence images confirmed that the MCTS treated with **1**–**7** in the dark and with **1**–**3** upon a 1P irradiation (500 nm, 16.7 min, 10.0 mW cm^−2^, and 10 J cm^−2^) or 2P irradiation (800 nm, 10 J cm^−2^, and section interval of 5 μm) are still intact. The low phototoxic effect of **1**–**3** is caused by the poor MCTS penetration. Promisingly, the MCTS treated with **4**–**7** and exposed to 1P or 2P light were completely eradicated.

The photodynamic effect using a 1P (500 nm, 16.7 min, 10.0 mW cm^−2^, and 10 J cm^−2^) and 2P irradiation (800 nm, 10 J cm^−2^, and section interval of 5 μm) was then quantified by determination of the ATP concentration (Supplementary Table 10). Importantly, no measurable cytotoxicity in the dark could be observed in HeLa MCTS for all compounds and **1**–**3** upon light exposure in agreement with the previous investigations. **4**–**6** were found to be phototoxic in the micromolar range (IC_50,500 nm_ = 6.8 ± 0.2 – 78.3 ± 5.1 μM, IC_50,800 nm_ = 1.4 ± 0.2 – 87.3 ± 6.8 μM). Strikingly, the lead compound of this study **7** was found to be nontoxic in the dark at even higher concentrations (IC_50,dark_ > 300 μM), while being highly phototoxic in the low micromolar range (IC_50,500 nm_ > 6.8 ± 0.2 μM, IC_50,800 nm_ > 1.4 ± 0.2) with exceptionally high PI values (PI_500 nm_ > 44, PI_800 nm_ > 250). Importantly, under identical experimental conditions, treatment with H_2_TPP did not show a cytotoxic effect (IC_50, dark_ = IC_50,500 nm_ = IC_50,800 nm_ > 100 μM), indicating that **7** is able to act at low drug and light doses compared to a clinically utilized tetrapyrrolic compound.

### Biological evaluation inside a mouse model

**Fig. 5:**
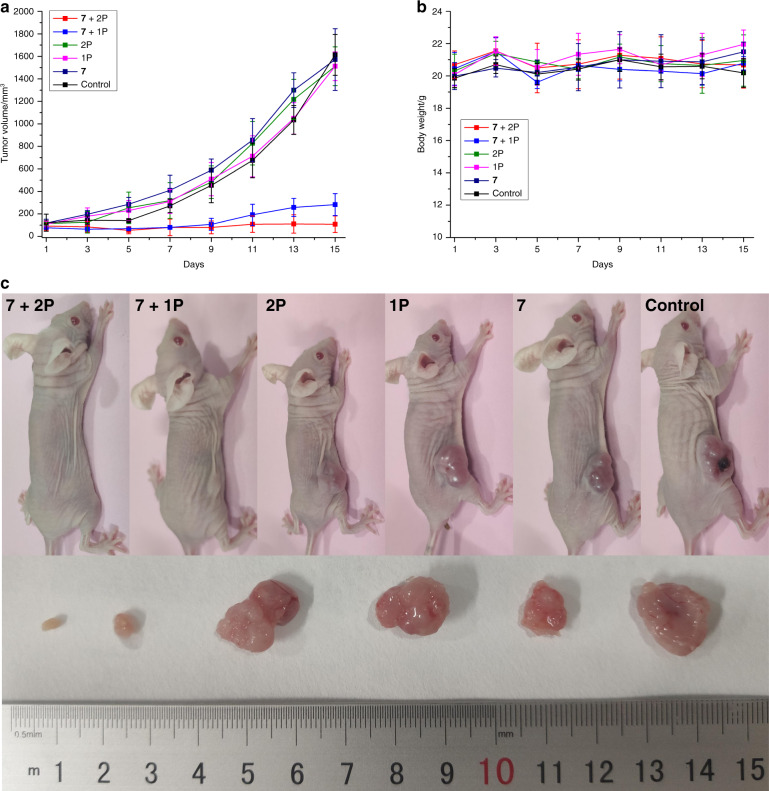
Biological Evaluation of **7** inside a mouse model. In vivo PDT study of **7** using 1P (500 nm, 60 min, 10.0 mW cm^−2^, and 36 J cm^−2^) or 2P (800 nm, 50 mW, 1 kHz, pulse width 35 fs, and 5 s mm^−1^) excitation on nude mice bearing a doxorubicin-selected P-gp-overexpressing human colon cancer tumor (SW620/AD300). **a** Tumor growth inhibition curves upon treatment. **b** Average body weights of the tumor-bearing mice. **c** Representative photographs of the tumor-bearing mice. The error bars correspond to the standard deviation of the five replicates.

Capitalizing on the superior photophysical (i.e., highest ^1^O_2_ production) and biological properties (i.e., highest phototoxicity in 2D monolayer cells and 3D MCTS, high cell penetration), the properties of **7** to act as a PDT PS were further investigated in a mouse model. To ensure solubility and biocompatibility, the PS was converted to a chloride salt using a counter ion exchange resin. We note at this stage that a change of the counter ion can have a significant effect on the biological properties of a metal complex. It was previously shown that a counter ion can cause an undesired cytotoxicity or the formation of nanoaggregates or a colloid due to poor water solubility^47^. It was also reported that the cellular uptake of a Ru(II) complex can dramatically change^48^. A challenging to treat multiresistant doxorubicin-selected P-gp-overexpressing human colon cancer tumor model (SW620/AD300) was used to evaluate the PS potential. The biodistribution of **7** (2 mg kg^−1^) inside this mouse model was then time-dependently (30 min, 1 h, 2 h) studied by intravenous tail injection into nude mice. After each time point, the mice were sacrificed and the major organs (i.e., blood, spleen, intestine, stomach, liver, kidney, uterus, lung, heart, brain, and tumor) were separated, ground and the Ru content determined by ICP-MS. Interestingly, **7** was absorbed from the blood stream within 1 h with a high accumulation in the intestine and some accumulation in the tumor (Supplementary Fig. 98). Following this, 1 h after an intravenous tail injection of **7** (2 mg kg^−1^), in vivo PDT experiments using a 1P (500 nm, 60 min, 10.0 mW cm^−2^, and 36 J cm^−2^) or 2P irradiation (800 nm, 50 mW, 1 kHz, pulse width 35 fs, and 5 s mm^−1^) were performed on mice with an 80 mm^3^ tumor. Encouragingly, after only one PDT treatment, the tumor drastically shrank until they were nearly eradicated whereas the tumors treated with the light or **7** in the dark (Fig. 5a, c) kept growing. Importantly, the animals treated with the compound behaved normally, without signs of pain, stress or discomfort and did not lose or gain weight (Fig. 5b). After the treatment, the mice were sacrificed, the tumor and organs were separated and histologically examined by an H&E stain. The tumor tissue treated with **7** and exposed to light displayed pathological alterations caused by the PDT treatment (Supplementary Fig. 99), while all other organs did not show any significant effect (Supplementary Fig. 100). Overall, this study demonstrates the enormous potential of **7** as a PS for 1P and 2P PDT.

In summary, Ru(II) polypyridine complexes with (*E*,*E*′)-4,4′-bisstyryl-2,2′-bipyridine ligands have been rationally designed using DFT calculations and were found to have a remarkable red-shifted 1P absorption as well as exceptionally high 2P cross sections. The complexes were found to be taken up by cancerous cells through an energy-dependent endocytosis pathway and to accumulate in the cytoplasm. Upon irradiation, they were found to generate ^1^O_2_, causing a phototoxic effect by apoptosis and paraptosis pathways in various monolayer cells as well as MCTS. In vivo studies confirmed the impressive ability of **7** to act as a PS upon treatment at clinically relevant 1P (500 nm) and 2P (800 nm) irradiation with an eradication of the tumor. Importantly, these compounds were found to be highly water soluble, stable in human plasma, as well as upon constant irradiation and therefore are able to overcome the limitations of currently employed PSs. We strongly believe that these complexes have great potential for preclinical trials.

## Methods

### Computational details

All calculations were performed at DFT (ground state) and TD-DFT (excited state) level using the global hybrid B3LYP functional. The Los Alamos (LANL2) effective core potential and the corresponding triple-zeta basis set was applied to describe the Ruthenium atom, with all other atoms were described by a Pople double-zeta basis set with a single set of polarization functions on non-hydrogen atoms (6–31G(d)). All structures used correspond to ground state minima on the ground state potential energy surfaces. Solvent effect (acetonitrile) were taken into account using the PCM model for structural optimizations while single point calculations in the gas phase were performed to simulate 1P and 2P absorption spectra. All calculations were performed using the Gaussian suite of programs except for the 2PA spectra which were simulated using the DALTON software.

### Materials

All chemicals were purchased from commercial sources and used without further purification. If necessary, solvents were dried over molecular sieves. The Ru(II) precursors Ru(DMSO)_4_Cl_2_^49^ and Ru(2,2′-bipyridine)_2_Cl_2_^50^ were synthesized according to previously reported procedures. The pooled human plasma was obtained from Biowest. The cell culture media and reagents were purchased from Fisher Scientific.

### Instrumentation and methods

^1^H and ^13^C-NMR spectra were measured on a Bruker 400 MHz or 500 MHz NMR spectrometer. Chemical shifts (*δ*) are reported in parts per million (ppm) referenced to tetramethylsilane (*δ* 0.00) ppm using the residual proton solvent peaks as internal standards and coupling constants (J) in Hertz (Hz). The multiplicity of the peaks is abbreviated as follows: *s* (singlet), *d* (doublet), *dd* (doublet of doublet), *t* (triplet), *m* (multiplet). ESI-MS experiments were carried out using a LTQ-Orbitrap XL from Thermo Scientific and operated in positive ionization mode with a spray voltage of 3.6 kV. No Sheath and auxiliary gas was used. Applied voltages were 40 and 100 V for the ion transfer capillary and the tube lens, respectively. The ion transfer capillary was held at 275 °C. Detection was achieved in the Orbitrap with a resolution set to 100,000 (at m z^−1^ 400) and a m z^−1^ range between 150 and 2000 in profile mode. Spectrum was analyzed using the acquisition software XCalibur 2.1 (Thermo Fisher Scientific). The automatic gain control allowed accumulation of up to 2 × 10^5^ ions for FTMS scans, maximum injection time was set to 300 ms and 1 µscan was acquired. Ten microliters was injected using a Thermo Finnigan Surveyor HPLC system (Thermo Fisher Scientific) with a continuous infusion of methanol at 100 µL min^−1^. Elemental microanalyses were performed on a Thermo Flash 2000 elemental analyzer. For analytic and preparative HPLC the following system has been used: 2 × Agilent G1361 1260 Prep Pump system with Agilent G7115A 1260 DAD WR Detector equipped with an Agilent Pursuit XRs 5C18 (Analytic: 100 Å, C18 5 μm, 250 × 4.6 mm, Preparative: 100 Å, C18 5 μm 250 × 300 mm) column and an Agilent G1364B 1260-FC fraction collector. The solvents (HPLC grade) were millipore water (0.1% trifluoroacetic acid, solvent A) and acetonitrile (0.1% trifluoroacetic acid, solvent B). ICP-MS experiments were carried out on an iCAP RQ ICP-MS instrument (Thermo Fisher).

### Synthesis

*(E,E*′)-*4,4*′-*Bisstyryl-2,2*′*-bipyridine*: The synthesis of (*E*,*E*′)-4,4′-Bisstyryl-2,2′-bipyridine is already published but in this study another synthetic route was employed. 4,4′-Dimethyl-2,2′-bipyridine (1000 mg, 5.43 mmol, 1.0 equiv.) was dissolved in dry *N*,*N*-dimethylformamide under nitrogen atmosphere and benzaldehyde (1.2 mL, 11.84 mmol, 2.2 equiv.) was added to the solution. Afterwards potassium *tert*-butoxide (2436 mg, 21.72 mmol, 4.0 equiv.) was added slowly. The color of the solution turned to green and the mixture was stirred for 24 h. After that the mixture was poured into water (400 mL) and the suspension cooled down to 5 °C. The crude product which precipitated, was filtered and washed with methanol. The product was purified by recrystallization from boiling acetic acid. The obtained solid was dissolved in dichloromethane and the mixture was washed with a 5% aqueous lithium chloride solution, brine and water. The solvent was removed and the product was isolated by recrystallization from boiling acetic acid. 1292 mg of (*E*,*E*′)-4,4′-Bisstyryl-2,2′-bipyridine (3.58 mmol, 66%) were yielded as a beige solid. The experimental data obtained is in agreement with the previous literature^51^.

*(E,E′)-4,4*′*-Bis[p-(N,N-dimethylamino)styryl]-2,2*′*-bipyridine*: The synthesis of (*E*,*E*′)-4,4′-Bis[*p*-(*N*,*N*-dimethylamino)styryl]-2,2′-bipyridine is already published but in this study another synthetic route was employed. 4,4′-Dimethyl-2,2′-bipyridine (1000 mg, 5.43 mmol, 1.0 equiv.) was dissolved in dry *N*,*N*-dimethylformamide (100 mL) under nitrogen atmosphere and potassium *tert*-butoxide (2437 mg, 21.72 mmol, 4.0 equiv.) was added slowly. After 1.5 h of stirring, 4-(dimethylamino)benzaldehyde (1701 mg, 11.40 mmol, 2.1 equiv.) was added to the reaction mixture. The color of the solution turned to yellow and the mixture was heated at 90 °C for 19 h. After that the mixture was poured into water (400 mL) and the suspension cooled down to 5 °C. The crude product which precipitated, was filtered and washed with water and diethyl ether. The product was isolated by recrystallization from dichloromethane/pentane. 1541 mg of (*E*,*E*′)-4,4′-Bis[*p*-(*N*,*N*-dimethylamino)styryl]-2,2′-bipyridine (3.45 mmol, 64%) were yielded as a yellow solid. The experimental data obtained is in agreement with the previous literature^52^.

*(E,E*′*)-4,4*′*-Bis[p-methoxystyryl]-2,2*′*-bipyridine*: The synthesis of (*E*,*E*′)-4,4′-Bis[*p*-methoxystyryl]-2,2′-bipyridine is already published but in this study another synthetic route was employed. 4,4′-Dimethyl-2,2′-bipyridine (532 mg, 2.89 mmol, 1.0 equiv.) was dissolved in dry *N*,*N*-dimethylformamide (25 mL) under nitrogen atmosphere and 4-methoxybenzaldehyde (0.88 mL, 7.22 mmol, 2.5 equiv.) was added to the solution. Afterwards potassium *tert*-butoxide (1360 mg, 12.13 mmol, 4.2 equiv.) was added slowly. The color of the solution turned to green and the mixture was stirred for 24 h. After that the mixture which turned bright was poured into water (200 mL) and the suspension cooled down to 5 °C. The crude product which precipitated, was filtered, and washed with methanol. The product was purified by recrystallization from boiling acetic acid. The obtained solid was dissolved in dichloromethane and the mixture was washed with a 5% aqueous lithium chloride solution, brine and water. The solvent was removed and the product was isolated by recrystallization from boiling acetic acid. 925 mg of (*E*,*E*′)-4,4′-Bis[*p*-methoxystyryl]-2,2′-bipyridine (2.20 mmol, 76%) were yielded as a beige solid. The experimental data obtained is in agreement with the previous literature^52^.

*[Ru(E,E′)-4,4′-Bisstyryl-2,2′-bipyridine)*_3_*][PF*_*6*_*]*_*2*_ *(****1****): (E,E*′*)*-4,4′-Bisstyryl-2,2′-bipyridine (400 mg, 1.11 mmol, 4.0 equiv.) and Ru(DMSO)_4_Cl_2_ (134 mg, 0.28 mmol, 1.0 equiv.) were suspended in dry ethanol (150 mL) under nitrogen atmosphere and the mixture was refluxed for 24 h. Then the solution was cooled down and undissolved residue was removed by filtration. To the residual solution a saturated aqueous solution of ammonium hexafluorophosphate was added. The crude product, which precipitated as a hexafluorophosphate salt was collected by centrifugation and washed with ethanol, water and diethyl ether. The product was dissolved in dichloromethane and washed with a 5% aqueous lithium chloride solution, brine and water. After drying, 323 mg of **1** (0.22 mmol, 79%) were yielded as a red solid. ^1^H-NMR (500 MHz, CD_3_CN): *δ* 8.76 (*d*, *J* = 1.8 Hz, 6H), 7.78 (*d*, *J* = 16.4 Hz, 6H), 7.76 (*d*, *J* = 5.9 Hz, 6H), 7.71–7.69 (*m*, 12H), 7.51 (dd, *J* = 5.9, 1.8 Hz, 6H), 7.49–7.46 (*m*, 12H), 7.44–7.40 (*m*, 6H), 7.33 (*d*, *J* = 16.4 Hz, 6H); ^13^C-NMR (125 MHz, CD_3_CN): *δ* 158.2, 152.4, 147.6, 137.3, 136.8, 130.6, 130.1, 128.4, 125.4, 125.1, 121.7; HRMS (*m*/*z*): [M]^2+^ calcd. for C_78_H_60_N_6_Ru, 591.1956; found, 591.1978; analysis (calcd., found for C_78_H_60_F_12_N_6_P_2_Ru + 4^.^H_2_O): C (60.66, 60.62), H (4.44, 4.43), N (5.44, 5.76). [Ru(*E*,*E*′)-4,4′-Bisstyryl-2,2′-bipyridine)_3_][Cl]_2_: The counter ion hexafluorophosphate was exchanged to chloride by elution with methanol from the ion exchange resin Amberlite IRA-410. Analysis (calcd., found for C_78_H_60_Cl_2_N_6_Ru): C (74.75, 74.36), H (4.83, 4.51), N (6.71, 6.37).

*[Ru((E,E*′*)-4,4*′*-Bis[p-(N,N-dimethylamino)styryl]-2,2*′*-bipyridine)*_*3*_*][PF*_*6*_*]*_*2*_ *(****2****)*: (*E*,*E*′)-4,4′-Bis[*p*-(*N*,*N*-dimethylamino)styryl]-2,2′-bipyridine (338 mg, 0.76 mmol, 4.0 equiv.), Ru(DMSO)_4_Cl_2_ (92 mg, 0.19 mmol, 1.0 equiv.) and lithium chloride (401 mg, 9.46 mmol, 50.0 equiv.) were dissolved in dry *N*,*N*-dimethylformamide (50 mL) under nitrogen atmosphere. The mixture was refluxed for 48 h. The solution was then cooled down and a saturated aqueous solution of ammonium hexafluorophosphate was added. The crude product, which precipitated as a hexafluorophosphate salt was collected by centrifugation and washed with ethanol, water and diethyl ether. The residue was dissolved in dichloromethane and washed with a 5% aqueous lithium chloride solution, brine and water. The solvent was removed under reduced pressure and the crude product recrystallized from dichloromethane/pentane. The product was isolated via fractionated precipitation from acetonitrile by adding dropwise diethyl ether. 86 mg of **2** (0.05 mmol, 26%) were yielded as a black solid. ^1^H-NMR (500 MHz, CD_3_CN): *δ* 8.62 (*d*, *J* = 1.9 Hz, 6H), 7.66 (*d*, *J* = 16.2 Hz, 6H), 7.65 (*d*, *J* = 6.1 Hz, 6H), 7.55–7.52 (*m*, 12H), 7.38 (dd, *J* = 6.1, 1.9 Hz, 6H), 7.03 (*d*, *J* = 16.2 Hz, 6H), 6.81–6.78 (*m*, 12H), 3.01 (*s*, 36H); ^13^C-NMR (125 MHz, CD_3_CN): *δ* 159.4, 158.1, 152.6, 148.3, 137.7, 130.0, 124.5, 124.4, 120.6, 119.7, 113.2, 40.4; HRMS (*m*/*z*): [M]^2+^ calcd. for C_90_H_90_N_12_Ru, 720.3222; found, 720.3247; analysis (calcd., found for C_90_H_90_F_12_N_12_P_2_Ru): C (62.46, 62.54), H (5.24, 5.17), N (9.71, 9.79). [Ru((*E*,*E*′)-4,4′-Bis[*p*-(*N*,*N*-dimethylamino)styryl]-2,2′-bipyridine)_3_][Cl]_2_: The counter ion hexafluorophosphate was exchanged to chloride by elution with methanol from the ion exchange resin Amberlite IRA-410. Analysis (calcd., found for C_90_H_90_Cl_2_N_12_Ru): C (71.51, 71.19), H (6.00, 5.93), N (11.12, 10.84).

*[Ru((E,E*′*)-4,4*′*-Bis[p-methoxystyryl]-2,2*′*-bipyridine)*_*3*_*][PF*_*6*_*]*_*2*_ *(****3****)*: (*E*,*E*′)-4,4′-Bis[*p*-methoxystyryl]-2,2′-bipyridine (286 mg, 0.68 mmol, 4.0 equiv.) and Ru(DMSO)_4_Cl_2_ (82 mg, 0.17 mmol, 1.0 equiv.) were suspended in dry ethanol (50 mL) under nitrogen atmosphere. The mixture was refluxed for 15 h. The solution was then cooled down and undissolved solid was removed by filtration. A saturated aqueous solution of ammonium hexafluorophosphate was added and the crude product, which precipitated as a hexafluorophosphate salt was collected by filtration. The solid was washed with water and diethyl ether. The residue was purified via fractionated precipitation from acetonitrile by adding dropwise diethyl ether. The collected product was dissolved in dichloromethane and washed with a 5% aqueous lithium chloride solution, brine and water. After drying, 218 mg of **3** (0.13 mmol, 76%) were yielded as a black solid. ^1^H-NMR (500 MHz, CD_3_CN): *δ* 8.79 (*d*, *J* = 1.7 Hz, 6H), 7.79 (*d*, *J* = 16.4 Hz, 6H), 7.71 (*d*, *J* = 6.0 Hz, 6H), 7.64 (*d*, *J* = 8.9 Hz, 12H), 7.44 (*d*, *J* = 6.0, 1.7 Hz, 6H), 7.17 (*d*, *J* = 16.4 Hz, 6H), 7.00 (*d*, *J* = 8.9 Hz, 12H), 3.83 (*s*, 18H). ^13^C-NMR (125 MHz, CD_3_CN): *δ* 162.0, 158.2, 152.2, 148.0, 137.0, 130.1, 129.5, 125.0, 122.7, 121.3, 115.5, 56.2; HRMS (*m*/*z*): [M]^2+^ calcd. for C_84_H_72_N_6_O_6_Ru, 681.2290; found, 681.2273; analysis (calcd., found for C_84_H_72_F_12_N_6_O_6_P_2_Ru): C (61.05, 61.17), H (4.39, 4.44), N (5.09, 5.21). [Ru((*E*,*E*′)-4,4′-Bis[*p*-methoxystyryl]-2,2′-bipyridine)_3_][Cl]_2_: The counter ion hexafluorophosphate was exchanged to chloride by elution with methanol from the ion exchange resin Amberlite IRA-410. Analysis (calcd., found for C_84_H_72_Cl_2_N_6_O_6_Ru): C (70.38, 70.62), H (5.06, 5.28), N (5.86, 5.57).

*[Ru(2,2*′*-bipyridine)((E,E*′*)-4,4*′*-Bis[p-(N,N-dimethylamino)styryl]-2,2*′*-bipyridine)*_*2*_*][PF*_*6*_*]*_*2*_ *(****4****)*: (*E*,*E*′)-4,4′-Bis[*p*-(*N*,*N*-dimethylamino)styryl]-2,2′-bipyridine (220 mg, 0.49 mmol, 2.0 equiv.), Ru(DMSO)_4_Cl_2_ (119 mg, 0.25 mmol, 1.0 equiv.), and lithium chloride (1044 mg, 24.63 mmol, 100 equiv.) were suspended in dry *N*,*N*-dimethylformamide (30 mL) under nitrogen atmosphere. The mixture was refluxed for 4 h. The solution was then cooled down and water was added. The crude product, which precipitated was collected by filtration and washed with water and diethyl ether. The formation of [Ru((*E*,*E*′)-4,4′-Bis[*p*-(*N*,*N*-dimethylamino)styryl]-2,2′-bipyridine)_2_Cl_2_] was analyzed via HPLC. [Ru((*E*,*E*′)-4,4′-Bis[*p*-(*N*,*N*-dimethylamino)styryl]-2,2′-bipyridine)_2_Cl_2_] and 2,2′-bipyridine (47 mg, 0.3 mmol, 1.2 equiv.) were suspended in dry ethanol (50 mL) under nitrogen atmosphere. The mixture was refluxed for 7 h. The solution was then cooled down and undissolved solid was removed by filtration. A saturated aqueous solution of ammonium hexafluorophosphate was added and the crude product, which precipitated as a hexafluorophosphate salt was collected by filtration. The solid was washed with water and diethyl ether. The residue was purified via preparative HPLC as a trifluoroacetic acid salt. The solvents were millipore water with 0.1% trifluoroacetic acid (solvent A) and acetonitrile (solvent B). The following HPLC gradient has been used: 0–3 min: isocratic 50% A (50% B); 3–17 min: linear gradient from 50% A (50% B) to 0% A (100% B); 17–23 min: isocratic 0% A (100% B). The flow rate was 20 mL min^−1^ and the chromatogram was detected at 250, 350, and 450 nm. The collected product was dissolved in water and a saturated aqueous solution of ammonium hexafluorophosphate was added. The product, which precipitated as a hexafluorophosphate salt was collected by filtration and washed with water, diethyl ether and pentane. 89 mg of **4** (0.06 mmol, 24%) were yielded as a dark red solid. ^1^H-NMR (400 MHz, CD_3_CN): *δ* 8.61 (*s*, 4H), 8.50 (*d*, *J* = 8.2 Hz, 2H), 8.04 (td, *J* = 8.0, 1.5 Hz, 2H), 7.86 (ddd, *J* = 5.7, 1.4, 0.6 Hz, 2H), 7.66 (dd, *J* = 16.2, 1.9 Hz, 4H), 7.64 (*d*, J = 6.3 Hz, 2H), 7.56–7.50 (*m*, 10H), 7.43–7.34 (*m*, 6H), 7.02 (dd, *J* = 16.2 Hz, 4H), 6.81–6.77 (*m*, 8H), 3.02 (*s*, 12H), 3.01 (*s*, 12H); ^13^C-NMR (100 MHz, CD_3_CN): *δ* 158.1, 158.0, 152.7, 152.6, 151.9, 151.9, 148.5, 138.3, 137.9, 130.0, 128.4, 125.1, 124.4, 120.7,119.6, 113.2, 40.4; HRMS (*m*/*z*): [M]^2+^ calcd. for C_70_H_68_N_10_Ru, 575.2330; found, 575.2347; analysis (calcd., found for C_70_H_68_F_12_N_10_P_2_Ru): C (58.37, 58.19), H (4.76, 4.62), N (9.72, 9.72).

*[Ru(2,2*′*-bipyridine)((E,E*′*)-4,4*′*-Bis[p-methoxystyryl]-2,2*′*-bipyridine)*_*2*_*][PF*_*6*_*]*_*2*_ *(****5****)*: (*E*,*E*′)-4,4′-Bis[*p*-methoxystyryl]-2,2′-bipyridine (490 mg, 1.17 mmol, 2.0 equiv.), Ru(DMSO)_4_Cl_2_ (282 mg, 0.58 mmol, 1.0 equiv.), and lithium chloride (2470 mg, 58.26 mmol, 100 equiv.) were suspended in dry *N*,*N*-dimethylformamide (75 mL) under nitrogen atmosphere. The mixture was refluxed for 6 h. The solution was then cooled down and purged into water. The crude product, which precipitated was collected by filtration and washed with water and diethyl ether. The formation of [Ru((*E*,*E*′)-4,4′-Bis[*p-*methoxystyryl]-2,2′-bipyridine)_2_Cl_2_] was analyzed via HPLC. [Ru((*E*,*E*′)-4,4′-Bis[*p*-methoxystyryl]-2,2′-bipyridine)_2_Cl_2_] and 2,2′-bipyridine (109 mg, 0.70 mmol, 1.2 equiv.) were suspended in dry ethanol (100 mL) under nitrogen atmosphere. The mixture was refluxed for 6 h. The solution was then cooled down and undissolved solid was removed by filtration. A saturated aqueous solution of ammonium hexafluorophosphate was added and the crude product, which precipitated as a hexafluorophosphate salt was collected by centrifugation. The solid was washed with water and diethyl ether. The residue was purified via preparative HPLC as a trifluoroacetic acid salt. The solvents were millipore water with 0.1% trifluoroacetic acid (solvent A) and acetonitrile (solvent B). The following HPLC gradient has been used: 0–3 min: isocratic 50% A (50% B); 3–17 min: linear gradient from 50% A (50% B) to 0% A (100% B); 17–23 min: isocratic 0% A (100% B). The flow rate was 20 mL min^−1^ and the chromatogram was detected at 250, 350, and 450 nm. The collected product was dissolved in water and a saturated aqueous solution of ammonium hexafluorophosphate was added. The product, which precipitated as a hexafluorophosphate salt was collected by filtration and washed with water, diethyl ether and hexane. 248 mg of **5** (0.18 mmol, 31%) were yielded as a dark red solid. ^1^H-NMR (400 MHz, CD_3_CN): *δ* 8.72 (*d*, *J* = 1.6 Hz, 4H), 8.51 (*d*, *J* = 8.2 Hz, 2H), 8.06 (td, *J* = 8.0, 1.3 Hz, 2H), 7.85 (dd, *J* = 5.6, 1.1 Hz, 2H), 7.75–7.68 (*m*, 6H), 7.67–7.61 (*m*, 8H), 7.60 (*d*, *J* = 5.9 Hz, 2H), 7.46–7.39 (*m*, 6H), 7.17 (dd, *J* = 16.4, 2.1 Hz, 4H), 7.05–6.99 (*m*, 8H), 3.85 (*s*, 6H), 3.84 (*s*, 6H); ^13^C-NMR (100 MHz, CD_3_CN): *δ* 162.0, 158.1, 152.6, 152.2, 148.1, 138.6, 137.1, 132.8, 130.1, 129.5, 128.5, 125.2, 125.0, 122.7, 121.3, 115.5, 56.2; HRMS (*m*/*z*): [M]^2+^ calcd. for C_66_H_56_N_6_O_4_Ru, 549.1698; found, 549.1707; analysis (calcd., found for C_66_H_56_F_12_N_6_O_4_P_2_Ru + C_6_H_14_): C (57.96, 58.33), H (4.44, 4.08), N (5.87, 5.79).

*[Ru(2,2*′*-bipyridine)*_*2*_*((E,E*′*)-4,4*′*-Bis[p-(N,N-dimethylamino)styryl]-2,2*′*-bipyridine)][PF*_*6*_*]*_*2*_ *(****6****)*: Ru(2,2′-bipyridine)_2_Cl_2_ (350 mg, 0.72 mmol, 1.0 equiv.) and (*E*,*E*′)-4,4′-Bis[*p*-(*N*,*N*-dimethylamino)styryl]-2,2′-bipyridine (388 mg, 0.87 mmol, 1.2 equiv.) were suspended in dry ethanol (50 mL) under nitrogen atmosphere and the mixture was refluxed for 6 h. Then the solution was cooled down and a saturated aqueous solution of ammonium hexafluorophosphate was added. The crude product, which precipitated as a hexafluorophosphate salt was collected by filtration and washed with water and diethyl ether. The product was isolated via fractionated precipitation from acetonitrile by adding dropwise diethyl ether. 449 mg of **6** (0.39 mmol, 54%) were yielded as a dark red solid. ^1^H-NMR (500 MHz, CD_3_CN): *δ* 8.62 (*d*, *J* = 1.7 Hz, 2H), 8.50 (*d*, *J* = 8.2 Hz, 4H), 8.07–8.02 (*m*, 4H), 7.87–7.84 (*m*, 2H), 7.75–7.72 (*m*, 2H), 7.67 (*d*, J = 16.3 Hz, 2H), 7.57–7.52 (*m*, 4H), 7.52–7.49 (*d*, *J* = 6.0 Hz, 2H), 7.44–7.34 (*m*, 6H), 7.02 (*d*, *J* = 16.3 Hz, 2H), 6.81–6.76 (*m*, 4H), 3.01 (*s*, 12H); ^13^C-NMR (125 MHz, CD_3_CN): *δ* 158.1, 158.0, 158.0, 152.7, 152.7, 152.6, 151.9, 148.8, 138.6, 138.0, 130.0, 128.5, 128.5, 125.2, 124.4, 124.3, 120.8, 119.5, 113.1, 40.4; HRMS (m/z): [M]^2+^ calcd. for C_50_H_46_N_8_Ru, 430.1439; found, 430.1441; analysis (calcd., found for C_50_H_46_F_12_N_8_P_2_Ru): C (52.22, 51.97), H (4.03, 4.04), N (9.74, 9.71).

*[Ru(2,2′-bipyridine)*_2_*((E,E′)-4,4′-Bis[p-methoxystyryl]-2,2′-bipyridine)][PF*_*6*_*]*_*2*_*(****7****)*: Ru(2,2′-bipyridine)_2_Cl_2_ (432 mg, 0.89 mmol, 1.0 equiv.) and (*E*,*E*′)-4,4′-Bis[*p*-methoxystyryl]-2,2′-bipyridine (450 mg, 1.07 mmol, 1.2 equiv.) were suspended in dry ethanol (100 mL) under nitrogen atmosphere and the mixture was refluxed for 6 h. Then the solution was cooled down and a saturated aqueous solution of ammonium hexafluorophosphate was added. The crude product, which precipitated as a hexafluorophosphate salt was collected by filtration and washed with water and diethyl ether. The product was isolated via fractionated precipitation from acetonitrile by adding dropwise diethyl ether. 358 mg of **7** (0.32 mmol, 36%) were yielded as a dark red solid. ^1^H-NMR (500 MHz, CD_3_CN): *δ* 8.71 (*d*, *J* = 1.4 Hz, 2H), 8.51 (dd, *J* = 8.2, 0.7 Hz, 4H), 8.06 (td, *J* = 8.0, 1.5 Hz, 4H), 7.86–7.84 (*m*, 2H), 7.76–7.73 (*m*, 2H), 7.72 (*d*, *J* = 16.4 Hz, 2H), 7.66–7.62 (*m*, 4H), 7.59 (*d*, *J* = 6.0 Hz, 2H), 7.45–7.38 (*m*, 6H), 7.16 (*d*, *J* = 16.4 Hz, 2H), 7.03–6.98 (*m*, 4H), 3.83 (*s*, 6H). ^13^C-NMR (125 MHz, CD_3_CN,): *δ* 162.0, 158.1, 158.0, 152.7, 152.6, 152.2, 148.2, 138.7, 138.7, 137.1, 130.1, 129.5, 128.6, 128.5, 125.2, 125.0, 122.6, 121.4, 115.5, 56.2; HRMS (*m*/*z*): [M]^2+^ calcd. for C_48_H_40_N_6_O_2_Ru, 417.1123; found, 417.1126; analysis (calcd., found for C_48_H_40_F_12_N_6_O_2_P_2_Ru): C (51.30, 51.23), H (3.59, 3.48), N (7.48, 7.61). [Ru(2,2′-bipyridine)_2_((*E*,*E*′)-4,4′-Bis[*p*-(*N*,*N*-methoxy)styryl]-2,2′-bipyridine)][Cl]_2_: The counter ion hexafluorophosphate was exchanged to chloride by elution with methanol from the ion exchange resin Amberlite IRA-410. Analysis (calcd., found for C_48_H_40_Cl_2_N_6_O_2_Ru): C (63.70, 63.51), H (4.46, 4.30), N (9.29, 9.11).

### X-ray crystallography

Single-crystal X-ray diffraction data were collected at 160(1) K for compounds L-H, L-NMe_2_, L-OMe, and **3** on a Rigaku OD XtaLAB Synergy Dualflex (Pilatus 200K detector) diffractometer associated with an Oxford liquid-nitrogen Cryostream cooler. The wavelength used for all experiments is 1.54184 Å and corresponds to the Cu K_α_ radiation (from a micro-focus sealed X-ray tube). The single crystals were selected and cut (if needed) in polybutene oil, mounted on a flexible loop, and transferred to the diffractometer on the goniometer head. The program suite CrysAlisPro (version 1.171.39.13a) was used for the pre-experiments, data collections, data reductions, and also for the analytical absorption corrections^53^ based on the indexed faces. Using the Olex2 software^54^, the crystal structures were solved with the SHELXT^55^ small molecule structure solution program and refined with the SHELXL program package^56^ by full-matrix least-squares minimization on *F*^2^. Molecular graphics were generated using Mercury 4.0^57^. The crystal data collections and structure refinement parameters are summarized in Tables S1 and S2. CCDC 1951466 (for **L-H**), CCDC 1951467 (for **3**), CCDC 1951468 (for L-NMe_2_), and CCDC 1951469 (for L-OMe) contain the supplementary crystallographic data for these compounds, and can be obtained free of charge from the Cambridge Crystallographic Data Center via www.ccdc.cam.ac.uk/data_request/cif.

In the crystal structure of L-H, the substituted bipyridine is located on a center of inversion, only one half of the molecule had to be refined, the second part being reproduced by a symmetry operation. The main species cocystallized with solvent molecules of acetic acid in a ratio 1:2, respectively.

In the crystal structure of L-OMe, there are two crystallographically independent bipyridine molecules in the asymmetric unit: one complete molecule and two half molecules on special positions for which the second half is reproduced by a symmetry operation. Solvent molecules of acetic acid cocrystallized with the main species in a ratio 2:1.

In the crystal structure of **3**, the asymmetric unit contains two dicationic Ru species, four anionic hexafluorophosphate counterions and one solvent molecule of tetrahydropyran (disordered over three positions). The model was difficult to refine because of the large number of non-hydrogen atoms in the asymmetric unit, and of the disorders observed for the flexible terminal C=C(H)-C_6_H_4_OCH_3_ groups. The crystal and the data collection were of a rather good quality but the final refinement parameters remained relatively high (*R*_1_ = 12% and wR_2_ = 40%) or poor (Goof = 1.5). The solvent molecules could be identified in residual density peaks but refinement was complicated and finally the real geometry of the tetrahydropyran molecule was substituted by a regular planar six-membered ring. Many restraints and constraints were used to get stable refinement cycles but the result seems to be reliable, especially there is no ambiguity about the main charged species.

### Spectroscopic measurements

The absorption spectra of the sample was measured with a SpectraMax M2 Spectrometer (Molecular Devices). For the measurements of the emission, the sample was irradiated at 355 nm with a NT342B Nd-YAG pumped optical parametric oscillator (Ekspla). The emission was focused at right angle to the excitation pathway and directed to a Princeton Instruments Acton SP-2300i monochromator. The signal was detected with a XPI-Max 4 CCD camera (Princeton Instruments).

### Two-photon absorption cross section

The two-photon absorption cross section spectra of the sample was determined upon direction comparison to Rhodamine B. The sample was irradiated with an OpoletteTM 355II (pulse width ≤ 100 fs, 80 MHz repetition rate, Spectra Physics Inc.) laser. The experimental excitation and detection conditions were conducted with negligible reabsorption processes, which could affect two-photon absorption measurements. The quadratic dependence of the two-photon induced luminescence intensity on the excitation power was verified at an excitation wavelength of 800 nm. The two-photon absorption cross section was calculated using the following equation:

1 $$\begin{array}{l}\sigma _{{\mathrm{2,}}{\mathrm{sample}}} \,=\, \sigma _{{\mathrm{2,}}{\mathrm{reference}}} \,\times\, \left( {{{\Phi }}_{{\mathrm{reference}}} \,\times\, {{c}}_{{\mathrm{reference}}} \,\times\, {{I}}_{{\mathrm{sample}}} \,\times\, {{n}}_{{\mathrm{sample}}}} \right)/\\ \left( {{{\Phi }}_{{\mathrm{sample}}} \,\times\, {{c}}_{{\mathrm{sample}}} \,\times\, {{I}}_{{\mathrm{reference}}} \,\times\, {{n}}_{{\mathrm{reference}}}} \right)\end{array},$$

σ_2_ = two-photon cross section, *Φ* = luminescence quantum yield, *c* = concentration, *I* = integrated luminescence intensity, *n* = refractive index

### Luminescence quantum yield

The sample was prepared in an acetonitrile solution with an absorption of 0.1 at 355 nm. The sample was irradiated at 355 nm with a NT342B Nd-YAG pumped optical parametric oscillator (Ekspla). The emission was focused at right angle to the excitation pathway and directed to a Princeton Instruments Acton SP-2300i monochromator. The signal was detected with a XPI-Max 4 CCD camera (Princeton Instruments). The luminescence quantum yields were determined by comparison with the reference [Ru(2,2′-bipyridine)_3_]Cl_2_ in acetonitrile (*Φ*_em_ = 5.9%)^58^ applying the following formula:

2 $${{\Phi }}_{{\mathrm{em,}}{\mathrm{sample}}} \,= \, {{\Phi }}_{{\mathrm{em, reference}}} \,\times\, \left( {{{F}}_{{\mathrm{reference}}}{{/F}}_{{\mathrm{sample}}}} \right) \\ \times\, \left( {{{I}}_{{\mathrm{sample}}}{{/I}}_{{\mathrm{reference}}}} \right) \,\times\, \left( {{{n}}_{{\mathrm{sample}}}{{/n}}_{{\mathrm{reference}}}} \right)^{\mathrm{2}},$$

3 $${{F}} \,=\, {\mathrm{1}}\,-\,{\mathrm{10}}^{{\mathrm{ - A}}}.$$

*Φ*_em_ = luminescence quantum yield, *F* = fraction of light absorbed, *I* = integrated emission intensities, *n* = refractive index, *A* = absorbance of the sample at irradiation wavelength.

### Lifetime

The sample was prepared in an air saturated as well as a degassed acetonitrile solution with an absorption of 0.2 at 355 nm. The sample was irradiated at 355 nm with a NT342B Nd-YAG pumped optical parametric oscillator (Ekspla). The emission was focused at right angle to the excitation pathway and directed to a Princeton Instruments Acton SP-2300i monochromator. The signal was detected with a R928 photomultiplier tube (Hamamatsu).

### Electron spin resonance (ESR)

The sample (10 μM) was dissolved in acetonitrile or PBS containing 20 mM TEMP (2,2,6,6–tetramethylpiperidine) as a ^1^O_2_ scavenger or 20 mM DMPO (5,5-dimethyl-1-pyrroline *N*-oxide) as a ^•^OOH or ^•^OH radical scavenger. Capillary tubes were filled with the solution and sintered by fire. EPR spectra were recorded on a Bruker A300 spectrometer with 1 G field modulation, 100 G scan range, and 20 mW microwave power. The samples were measured in exclusion from light and after irradiation (450 ± 10 nm, 60 s, and 21.8 mW cm^−2^).

### Singlet oxygen

*Direct evaluation*: the sample was prepared in an air-saturated acetonitrile or deuterated water solution with an absorption of 0.2 at 450 nm. The sample was irradiated at 450 nm with a mounted M450LP1 LED (Thorlabs), whose light was focused with aspheric condenser lenses. Using a T-Cube LED Driver (Thorlabs), the intensity of the irradiation was varied and monitored with an optical power and energy meter. The emission was focused at right angle to the excitation pathway and directed to a Princeton Instruments Acton SP-2300i monochromator. To cut off light at wavelengths shorter than 850 nm, a longpass glass filter was placed in front of the monochromator entrance slit. The signal was detected with an EO-817L IR-sensitive liquid-nitrogen cooled germanium diode detector (North Coast Scientific Corp.). The luminescence signal, centered at 1270 nm, was measured from 1100 to 1400 nm. The obtained data were analyzed upon plotting the integrated luminescence peaks against the percentage of the irradiation intensity. The slope of the linear regression was calculated and compared with the reference Rose Bengal (*Φ* = 76%)^59^. The absorption of the sample was corrected with an absorption correction factor. The singlet oxygen quantum yields were calculated using the following formula:

4 $${{\Phi }}_{{\mathrm{sample}}} \,=\, {{\Phi }}_{{\mathrm{reference}}} \,\times\, \left( {{{S}}_{{\mathrm{sample}}}{{/S}}_{{\mathrm{reference}}}} \right) \,\times\, \left( {{{I}}_{{\mathrm{reference}}}{{/I}}_{{\mathrm{sample}}}} \right),$$

5 $${{I \,=\, I}}_{\mathrm{0}} \,\times\, \left( {{\mathrm{1}}\,-\,{\mathrm{10}}^{{\mathrm{ - A}}}} \right).$$

*Φ* = singlet oxygen quantum yield, *S* = slope of the linear regression of the plot of the areas of the singlet oxygen luminescence peaks against the irradiation intensity, *I* = absorption correction factor, *I*_0_ = light intensity of the irradiation source, and *A* = absorption of the sample at irradiation wavelength.

*Indirect evaluation*: measurement in acetonitrile: the sample was prepared in an air-saturated acetonitrile solution with an absorption of 0.2 at the irradiation wavelength, *N*,*N*-dimethyl-4-nitrosoaniline aniline (RNO, 24 µM) and imidazole (12 mM). Measurement in PBS buffer: the sample was prepared in an air-saturated PBS solution containing the complex with an absorption of 0.2 at the irradiation wavelength, *N*,*N*-dimethyl-4-nitrosoaniline aniline (RNO, 20 µM) and histidine (10 mM). The samples were irradiated for various time points with an Atlas Photonics LUMOS BIO irradiator. The absorption of the samples was constantly monitored with a SpectraMax M2 Microplate Reader (Molecular Devices). The difference in absorption (*A*_0_ − *A*) at 420 nm for the measurement in acetonitrile or at 440 nm for the measurement in PBS was determined. The difference in absorption was then plotted against the irradiation times and the slope of the linear regression calculated. The absorption of the sample was corrected with an absorption correction factor. The singlet oxygen quantum yields were calculated using the same formulas as used for the direct evaluation.

### Stability in human plasma

The stability of the sample was evaluated upon incubation in pooled human plasma with caffeine as an internal standard, which previously demonstrated to be stable under these conditions^60^. The sample (20 μM) and caffeine (40 μM) were prepared in dimethyl sulfoxide stock solutions. One aliquot of both solutions was added to 975 μL of human plasma achieving a total volume of 1000 μL. The resulting solutions were incubated upon continuous gentle shaking (ca. 300 rpm) for 48 h at 37 °C. After this time, the incubation was ended by addition of 3 mL of methanol. The mixture was centrifuged for 60 min at 3000 rpm/958 gand 4 °C. The solution was filtered through a 0.2 μm membrane filter. The solvent was evaporated and the residue was redissolved in 1:1 (v/v) acetonitrile/water with 0.1% trifluoroacetic acid. The resulting solution was then filtered through a 0.2 μm membrane filter and analyzed with a HPLC System. The solvents (HPLC grade) were millipore water with 0.1% trifluoroacetic acid (solvent A) and acetonitrile (solvent B). Method M1: 0–3 min: isocratic 50% A (50% B); 3–17 min: linear gradient from 50% A (50% B) to 0% A (100% B); Method M2: 0–3 min: isocratic 95% A (5% B); 3–17 min: linear gradient from 95% A (5% B) to 0% A (100% B); 17–23 min: isocratic 0% A (100% B). The flow rate was 1 mL min^−1^ and the chromatogram was detected at 250 nm.

### Photostability

The sample was prepared in an air-saturated acetonitrile solution. Using a Atlas Photonics LUMOS BIO irradiator, the sample was constantly irradiated at 450 nm in time intervals from 0 to 10 min (13.2 J cm^−2^). The absorption spectra from 350 to 700 nm was monitored with a SpectraMax M2 Microplate Reader (Molecular Devices) at each time interval. As positive and negative controls, respectively, [Ru(2,2′-bipyridine)_3_]Cl_2_ and Protoporphyrin IX were used.

### Photobleaching

The sample was dissolved in air saturated 0.7 mL CD_3_CN and stored in a NMR tube in the dark. A ^1^H-NMR spectrum was measured directly after preparation to ensure the identity of the sample. The sample was then irradiated at 450 nm with an Atlas Photonics LUMOS BIO irradiator (10 min, 13.2 J cm^−2^). Following this treatment, a ^1^H-NMR spectrum was again measured.

### Distribution coefficient

Using the “shake-flask” method, the distribution coefficient between the PBS and octanol phase of the sample was determined. The PBS and octanol phases were previously saturated in each other. The sample was dissolved in the phase, which is dissolving best the compound. This solution was added to an equal volume of the other phase and mixed at 190 rpm overnight with a sample mixer. Following this, the solutions were equilibrated for 4 h. The phases were carefully separated from each other. The amount of the sample in each phase was determined by ICP-MS. The evaluation was repeated three times and the ratio between the organic and aqueous phase determined.

### Cell culture

Human glioblastoma astrocytoma (U373) cells were cultured in MEM medium supplemented with 10% FBS, 1% NEAA (nonessential amino acids), and 1% penicillin/streptomycin. Human cervical carcinoma (HeLa), doxorubicin-resistant human colon adenocarcinoma (SW620/AD300), and the mouse colon carcinoma (CT-26) cells were cultured in DMEM medium supplemented with 10% FBS and 1% penicillin/streptomycin. Human retinal epithelial (RPE-1) cells were cultured in DMEM-F12 medium supplemented with 10% FBS and 1% penicillin/streptomycin. All cell lines were obtained from the American Type Culture Collection and cultured at 37 °C and 5% CO_2._ Before an experiment, the cells were passaged three times.

### Time-dependent cellular uptake

A total of 6 × 10^6^ cells were incubated with the sample (10 μM, 2% DMSO, *v*%) for varying time points (0.5, 1, 2, 4, 8, 12 h) at 37 °C. After this time, the media was removed and the cells washed with cell media. The cells were trypsinised, harvested, centrifuged, and resuspended. The number of cells on the dish was counted. The sample was digested using a 60% HNO_3_ solution for 3 days and, following this, diluted to a 2% HNO_3_ solution in water. The Ru content was determined with an ICP-MS apparatus and the results compared with the Ru references. The Ru content was then associated with the number of cells.

### Cellular uptake mechanism

The cellular uptake mechanism was investigated upon pretreatment of 1 × 10^6^ cells with pathways inhibitors and determination of the amount of Ru inside the cells with an ICP-MS apparatus.

*Control*: the cells were incubated with the sample (10 μM, 2% DMSO, *v*%) for 1 h at 37 °C and then washed with PBS. The cells were detached with trypsin, harvested, centrifuged, and resuspended. The number of cells on the dish was counted. The sample was digested using a 60% HNO_3_ solution for 3 days and following this diluted to a 2% HNO_3_ solution in water. The Ru content was determined with an ICP-MS apparatus and the results compared with the Ru references. The Ru content was then associated with the number of cells.

*Low temperature*: the cells were preincubated at 4 °C for 1 h. After this time, the cells were incubated with the sample (10 μM, 2% DMSO, *v*%) for 1 h at 37 °C and then washed with PBS. The cells were detached with trypsin, harvested, centrifuged, and resuspended. The number of cells on the dish was counted. The sample was digested using a 60% HNO_3_ solution for 3 days and following this diluted to a 2% HNO_3_ solution in water. The Ru content was determined with an ICP-MS apparatus and the results compared with the Ru references. The Ru content was then associated with the number of cells.

*Metabolic inhibition*: the cells were preincubated with 2-deoxy-*D*-glucose (50 mM) and oligomycin (5 μM) for 1 h. After this time, the cells were incubated with the sample (10 μM, 2% DMSO, *v*%) for 1 h at 37 °C and then washed with PBS. The cells were detached with trypsin, harvested, centrifuged, and resuspended. The number of cells on the dish was counted. The sample was digested using a 60% HNO_3_ solution for 3 days and following this diluted to a 2% HNO_3_ solution in water. The Ru content was determined with an ICP-MS apparatus and the results compared with the Ru references. The Ru content was then associated with the number of cells.

*Endocytic inhibition*: the cells were preincubated with ammonium chloride (50 mM) or chloroquine (100 μM) for 1 h. After this time, the cells were incubated with the sample (10 μM, 2% DMSO, *v*%) for 1 h at 37 °C and then washed with PBS. The cells were detached with trypsin, harvested, centrifuged, and resuspended. The number of cells on the dish was counted. The sample was digested using a 60% HNO_3_ solution for 3 days and following this diluted to a 2% HNO_3_ solution in water. The Ru content was determined with an ICP-MS apparatus and the results compared with the Ru references. The Ru content was then associated with the number of cells.

*Cation transporter inhibition*: the cells were preincubated with tetraethylammonium chloride (1 mM) for 1 h. After this time, the cells were incubated with the sample (10 μM, 2% DMSO, *v*%) for 1 h at 37 °C and then washed with PBS. The cells were detached with trypsin, harvested, centrifuged, and resuspended. The number of cells on the dish was counted. The sample was digested using a 60% HNO_3_ solution for 3 days and following this diluted to a 2% HNO_3_ solution in water. The Ru content was determined with an ICP-MS apparatus and the results compared with the Ru references. The Ru content was then associated with the number of cells.

### Intracellular distribution by confocal luminescence imaging

A total of 1 × 10^4^ cells were seeded on confocal dishes and allowed to adhere overnight. The cells were incubated with the sample (10 μM, 2% DMSO, *v*%) for 8 h at 37 °C in the dark. After this time, the cells were washed with PBS. The cells were further each incubated with LysoTracker Green, MitoTracker Deep Red, Cell-light Golgi-GFP, ER tracker Green and Hoechst 33342 for 30 min at 37 °C in the dark. The cells were washed three times with PBS. Confocal images were taken with a LSM 880 (Carl Zeiss) laser scanning confocal microscope. The organelle trackers were excited and detected as recommended by the supplier. The samples were detected under usage of their 1-Photon (*λ*_ex_ = 458 nm, *λ*_em_ = 600–750 nm) or 2-Photon (*λ*_ex_ = 800 nm, *λ*_em_ = 600–750 nm) luminescence properties.

### Intracellular distribution by ICP-MS

A total of 10 × 10^6^ cells were incubated with the sample (10 μM, 2% DMSO, *v*%) for 8 h at 37 °C in the dark. After this time, the cells were detached with trypsin and harvested. The number of cells was counted and equally divided into four portions. In the first portion, the nucleus was extracted using a nucleus extraction kit (Sangon Biotech); in the second portion, the mitochondria was extracted using a mitochondria extraction kit (Sangon Biotech), and in the third portion, the lysosome was extracted using a lysosome extraction kit (GenMed Scientific). Using the fourth portion, the cytoplasm was extracted. The cells were detached with trypsin, harvested, and centrifuged. The cell pellet was resuspended in lysis buffer and the cells lysed. The cytoplasm was separated by centrifugation(Optima MAX-XP ultracentrifuge, Beckman Coulter) for 150 min at 200,000 g at 4 °C. The supernatant solution was separated. The sample was digested using a 60% HNO_3_ solution for 3 days and following this diluted to a 2% HNO_3_ solution in water. The Ru content was determined with an ICP-MS apparatus and the results compared with the Ru references. The Ru content was then associated with the number of cells.

### One-photon induced cellular singlet oxygen generation

A total of 1 × 10^4^ cells were seeded on confocal dishes and allowed to adhere overnight. The cells were incubated with the sample (10 μM, 2% DMSO, *v*%) for 4 h at 37 °C in the dark. After this time, the cells were washed with PBS. The cells were further incubated with 2′,7′-dichlorofluorescein diacetate for 30 min. The cells were washed three times with PBS. The sample was irradiated upon 1-Photon excitation (*λ*_ex_ = 488 nm). Confocal images (*λ*_ex_ = 488 nm, *λ*_em_ = 510–550 nm) were taken with a LSM 880 (Carl Zeiss) laser scanning confocal microscope.

### Two-photon induced cellular singlet oxygen generation

A total of 1 × 10^4^ cells were seeded on confocal dishes and allowed to adhere overnight. The cells were incubated with the sample (10 μM, 2% DMSO, *v*%) for 4 h at 37 °C in the dark. After this time, the cells were washed with PBS. The cells were further incubated with 2′,7′-dichlorofluorescein diacetate for 30 min. The cells were washed three times with PBS. The sample was irradiated upon 2-Photon excitation (*λ*_ex_ = 800 nm). Confocal images (*λ*_ex_ = 488 nm, *λ*_em_ = 510–550 nm) were taken with a LSM 880 (Carl Zeiss) laser scanning confocal microscope.

### (Photo-)cytotoxicity on 2D cell monolayers

A total of 4 × 10^3^ cells were seeded on 96-well plates and allowed to adhere overnight. The cells were treated with increasing concentrations of the sample diluted in cell media achieving a total volume of 200 μL. The cells were incubated with the sample for 8 h and, after this time, the medium refreshed. To study the phototoxic effect of the sample, the cells were exposed to 480 nm (spectral half-width: 20 nm, 10 min, 5.2 mW cm^−2^, 3.1 J cm^−2^) or 540 nm (spectral half-width: 30 nm, 40 min, 4.0 mW cm^−2^, 9.5 J cm^−2^) light using an Atlas Photonics LUMOS BIO irradiator. To study the dark cytotoxicity of the sample, the cells were not irradiated and the medium exchanged. The cells were grown for an additional 40 h at 37 °C. After this time, the medium was replaced with fresh medium containing resazurin with a final concentration of 0.2 mg mL^−1^. The cells were incubated for 4 h and the amount of the fluorescent product resorufin determined (*λ*_ex_ = 540 nm, *λ*_em_ = 590 nm) with a SpectraMax M2 Microplate Reader (Molecular Devices). The obtained data was analyzed with the GraphPad Prism software.

### Cell death mechanism

The cell death mechanism was investigated by measuring the cell viability upon preincubation with various cell death pathway inhibitors. 3-Methyladenine (100 μM), Z-VAD-FMK (20 μM), cycloheximide (0.1 μM), and necrostatin-1 (60 μM) were preincubated with the cells for 40 min. Following this, the sample (5 μM) was added and incubated for 8 h. The cells were washed, irradiated with a LED (500 nm, 10 min, 10.0 mW cm^−2^, 6 J cm^−2^) light source, and incubated for 12 h. After this time, 3-(4,5-dimethylthiazol-2-yl)-2,5-diphenyltetrazolium bromide was added and the cells were incubated for additional 4 h. The liquid was disposed and 150 μL DMSO was added. The plates were shaken for 2 min and the absorbance at 595 nm was measured using a microplate reader (TecanInfiniteF200, Bio-rad).

### Generation and analysis of 3D multicellular tumor spheroids (MCTS)

A suspension of 0.75% agarose in PBS was heated inside a high-pressure autoclave. The hot emulsion was transferred into a 96-well plate (50 μL per well). The plates were exposed for 3 h to UV irradiation and allowed to cool down. After this time, a cell suspension of 3 × 10^3^ cells was seeded on top the agarose ground layer. Within 2–3 days, MCTS were formed from the cell suspension. The MCTS were cultivated and maintained at 37 °C in a cell culture incubator at 37 °C with 5% CO_2_ atmosphere. The culture medium was replaced every 2 days. The formation, integrity, diameter, and volume of the MCTS was monitored by an Axio Observer Z1 (Carl Zeiss) phase contrast microscope. The volume was calculated using the following equation: *V* = 4/3πr^3^. The luminescence images along the z-axis were captured by 1-Photon (*λ*_ex_ = 458 nm, *λ*_em_ = 600–750 nm) or 2-Photon (*λ*_ex_ = 800 nm, *λ*_em_ = 600–750 nm) excitation in the z-stack mode with a an LSM 880 (Carl Zeiss) laser scanning confocal microscope equipped with Argon or Coherent Chameleon 2-Photon laser and a GaAsP detector.

### 3D multicellular tumor spheroids (MCTS) growth inhibition assay

MCTS were treated with the sample (20 μM **1**–**7**, 20 μM tetraphenylporphyrin H_2_TPP, 10 μM cisplatin, 30 μM cisplatin, 2% DMSO, *v*%) by replacing 50% of the media with drug supplemented media in the dark for 3 days. After this time, the MCTS were exposed to a 1-Photon (500 nm, 16.7 min, 10.0 mW cm^−2^, and 10 J cm^−2^) irradiation using a LED light source or 2-Photon (800 nm, 10 J cm^−2^ with a section interval of 5 μm) irradiation using a LSM 880 (Carl Zeiss) laser scanning confocal microscope equipped with a Coherent Chameleon 2-Photon laser. The cell culture media was replaced every 2 days. The integrity and diameter of the MCTs was monitored with an Axio Observer Z1 (Carl Zeiss) phase contrast microscope every 24 h.

### 3D multicellular tumor spheroids (MCTS) viability assay

MCTS were treated with the sample (20 μM, 2% DMSO, *v*%) by replacing 50% of the media with drug supplemented media in the dark for 3 days. After this time, the MCTS were exposed to a 1-Photon (500 nm, 16.7 min, 10.0 mW cm^−2^, and 10 J cm^−2^) irradiation using a LED light source or 2-Photon (800 nm, 10 J cm^−2^ with a section interval of 5 μm) irradiation using a LSM 880 (Carl Zeiss) laser scanning confocal microscope equipped with a Coherent Chameleon 2-Photon laser. The cell culture media was replaced every 2 days. Two days after the irradiation the MCTS viability was tested using a Viability/Cytotoxicity Kit for mammalian cells (Invitrogen). Living cells can be identified from dead cells through the presence of ubiquitous intracellular esterase activity which can be monitored by the enzymatic conversion of the non-fluorescent calcein AM to the intensely fluorescent calcein (*λ*_ex_ = 495 nm, *λ*_em_ = 515 nm). As the spectroscopic properties of the dead cell probe EthD-1 overlaps with the one of the investigated compounds, this probe was not used and only calcein AM as a probe for living cells was used. The MCTS were incubated with calcein AM (2 μM) for 30 min and images of the MCTS taken with an Axio Observer Z1 (Carl Zeiss, Germany) inverted fluorescence microscope.

### (Photo-)cytotoxicity on 3D multicellular tumor spheroids (MCTS)

The cytotoxicity of the compounds in 3D MCTS was assessed by measurement of the ATP concentration. MCTS were treated with increasing concentrations of the compound (2% DMSO, *v*%) by replacing 50% of the media with drug supplemented media and incubation for 12 h. After this time, the MCTS were divided in three identical groups. The first group was strictly kept in the dark. The second group was exposed to a 1-Photon irradiation (500 nm, 16.7 min, 10.0 mW cm^−2^, and 10 J cm^−2^) using a LED light source and the third group was exposed to a 2-Photon irradiation (800 nm, 10 J cm^−2^ with a section interval of 5 μm) using a LSM 880 (Carl Zeiss, Germany) laser scanning confocal microscope equipped with a Coherent Chameleon 2-Photon laser. After the irradiation, all groups were incubated additional 48 h. The ATP concertation was measured using a CellTiter-Glo 3D Cell Viability kit (Promega) by measuring the generated chemiluminescence with an infinite M200 PRO (Tecan) plate reader. The obtained data was analyzed with the GraphPad Prism software.

### In vivo experiment

Six weeks old nu/nu female mice were purchased from Charles River. For the tumor xenograft, 6 × 10^6^ SW620/AD300 cells were subcutaneously (s.c.) injected with a 150 μL Matrigel and saline (1:1, *v/v*) suspension into the mice. After a week, the tumor volumes of the mice reached ~80 mm^3^. All experiments were conducted in compliance with the ethical regulations for animal testing and received approval of the experimental animal managing committee of Sun Yat Sen University.

### In vivo biodistribution

Three nude mice were tail-intravenous (i.v.) injected with 2 mg kg^−1^ of the sample. One hour later, the mice were sacrificed and the major organs (including the heart, liver, spleen, lung, kidney, brain, intestine, and blood) were separated, weighted and ground. The grinded organ as well as the blood were digested using a 60% HNO_3_ solution for 3 days, and following this, diluted to a 2% HNO_3_ solution in water. The Ru content was determined with an ICP-MS apparatus and the results compared with the Ru references.

### In vivo time-dependent biodistribution

Six SW620/AD300 nude tumor-bearing nude mice were separated into three groups, and i.v. injected with 2 mg kg^−1^ of the sample. 30 min, 1 h, 2 h later, the mice were euthanized and the distribution of the sample determined using the previous described method.

### In vivo (Photo-)cytotoxicity

30 SW620/AD300 nude tumor-bearing nude mice were randomly separated into six groups, resulting in five mice for each group. After been anaesthetized, the mice was fixed in a warm three-axes holder. Before irradiation, the tumor site was disinfected with ethanol. The tumor was exposed to 1P or 2P light. Following the irradiation, the operative area was disinfection by iodophor.

*Group 1*: injected **7** (2 mg kg^−1^, 50 μL) intravenously and irradiate under 800 nm laser (50 mW, 1 kHz, pulse width 35 fs, 5 s mm^−1^) 1 h after injection;

*Group 2*: injected **7** (2 mg kg^−1^, 50 μL) intravenously and irradiate under 500 nm light (60 min, 10.0 mW cm^−2^, 36 J cm^−2^) 1 h after injection;

*Group 3*: injected by physiological saline (50 μL) and treated with 800 nm laser (50 mW, 1 kHz, pulse width 35 fs, 5 s mm^−1^) 1 h after injection;

*Group 4*: injected by physiological saline (50 μL) and treated with 500 nm light (60 min, 10.0 mW cm^−2^, 36 J cm^−2^) 1 h after injection;

*Group 5*: intravenous injected **7** (2 mg kg^−1^, 50 μL);

*Group 6*: injected 50 μL physiological saline.

The mice were anesthetized by injection of a 4% chloral hydrate aqueous solution (0.2 mL, 20 g^−1^) before the treatment. The tumor volume and body weight were measured and recorded every 2 days. Tumor volume was calculated by the following formula:

6 $${\mathrm{Volume}} \,=\, \left( {{\mathrm{Length}} \,\times\, {\mathrm{Width}}^{\mathrm{2}}} \right){\mathrm{/2}}.$$

### Histological examination

The tumor and all major organs (including the heart, liver, spleen, lung, kidney, brain, intestine, and blood) were collected and a slice of each one was fixated with 4% paraformaldehyde. The obtained slices were stained with hematoxylin and eosin. The histological images were taken with a Carl Zeiss Axio Imager Z2 microscope.

### Reporting summary

Further information on research design is available in the Nature Research Reporting Summary linked to this article.

## Supplementary information

Supplementary Information

Reporting Summary

## Data Availability

The authors declare that all data supporting the findings of this study are available within the paper and its Supplementary Information files. Correspondence and requests for materials should be addressed to H.C. or G.G.
