# Supplementary material for: Rationally designed ruthenium complexes for 1- and 2-photon photodynamic therapy
Source: Nat Commun. 2020 Jun 26;11:3262. doi: 10.1038/s41467-020-16993-0 (PMC7320011; doi:10.1038/s41467-020-16993-0)
Supplement: Supplementary file 1 — Supplementary Information [file 41467_2020_16993_MOESM1_ESM.pdf]

Supplementary Information:

Rationally Designed Ruthenium Complexes for 1- and 2-Photon Photodynamic Therapy

Karges et al.

TABLE OF CONTENT

Figure 1. Simulated absorption spectra of compounds **1**, **3**, **5** and **7**. 7

Table 1. Theoretical data for **1**. 7

Table 2. Theoretical data for **3**. 10

Table 3. Theoretical data for **5**. 12

Table 4. Theoretical data for **7**. 14

Figure 2. Synthesis of the (*E*,*E*')-4,4′-bisstyryl-2,2′-bipyridine based ligands. 16

Figure 3. Synthesis of **1**-**3**. 17

Figure 4. Synthesis of **4**-**7**. 18

Figure 5. ^{1}H NMR spectrum of **1** in CD_3CN. 19

Figure 6. ^{13}C NMR spectrum of **1** in CD_3CN. 19

Figure 7. ESI-HRMS spectrum of **1**. 20

Figure 8. ^{1}H NMR spectrum of **2** in CD_3CN. 20

Figure 9. ^{13}C NMR spectrum of **2** in CD_3CN. 21

Figure 10. ESI-HRMS spectrum of **2**. 21

Figure 11. ^{1}H NMR spectrum of **3** in CD_3CN. 22

Figure 12. ^{13}C NMR spectrum of **3** in CD_3CN. 22

Figure 13. ESI-HRMS spectrum of **3**. 23

Figure 14. ^{1}H NMR spectrum of **4** in CD_3CN. 23

Figure 15. ^{13}C NMR spectrum of **4** in CD_3CN. 24

Figure 16. ESI-HRMS spectrum of **4**. 24

Figure 17. ^{1}H NMR spectrum of **5** in CD_3CN. 25

Figure 18. ^{13}C NMR spectrum of **5** in CD_3CN. 25

Figure 19. ESI-HRMS spectrum of **5**. 26

Figure 20. ^{1}H NMR spectrum of **6** in CD_3CN. 26

Figure 21. ^{13}C NMR spectrum of **6** in CD_3CN. 27

Figure 22. ESI-HRMS spectrum of **6**. 27

Figure 23. ^{1}H NMR spectrum of **7** in CD_3CN. 28

Figure 24. ^{13}C NMR spectrum of **7** in CD_3CN. 28

Figure 25. ESI-HRMS spectrum of **7**. 29

Table 5. Crystal data and structure refinement parameters for L-H and L-NMe_2. 30

Table 6. Crystal data and structure refinement parameters for L-OMe and **3**. 31
Figure 26. Molecular structure of L-H. 32
Figure 27. Molecular structure of L-NMe_2. 32
Figure 28. Molecular structure of L-OMe. 33
Figure 29. Molecular structure of **3**. 33
Figure 30. Normalized emission spectra of **1**-**7**. 34
Figure 31. Lifetime spectra of the complex **1** in aerated and degassed CH_3CN. 35
Figure 32. Lifetime spectra of the complex **2** in aerated and degassed CH_3CN. 36
Figure 33. Lifetime spectra of the complex **3** in aerated and degassed CH_3CN. 37
Figure 34. Lifetime spectra of the complex **4** in aerated and degassed CH_3CN. 38
Figure 35. Lifetime spectra of the complex **5** in aerated and degassed CH_3CN. 39
Figure 36. Lifetime spectra of the complex **6** in aerated and degassed CH_3CN. 40
Figure 37. Lifetime spectra of the complex **7** in aerated and degassed CH_3CN. 41
Figure 38. ESR spectra of the complex **1** trapped by TEMP in CH_3CN or PBS. 42
Figure 39. ESR spectra of the complex **2** trapped by TEMP in CH_3CN or PBS. 43
Figure 40. ESR spectra of the complex **3** trapped by TEMP in CH_3CN or PBS. 44
Figure 41. ESR spectra of the complex **4** trapped by TEMP in CH_3CN or PBS. 45
Figure 42. ESR spectra of the complex **5** trapped by TEMP in CH_3CN or PBS. 46
Figure 43. ESR spectra of the complex **6** trapped by TEMP in CH_3CN or PBS. 47
Figure 44. ESR spectra of the complex **7** trapped by TEMP in CH_3CN or PBS. 48
Figure 45. HPLC chromatogram of **1** after incubation in human plasma. 49
Figure 46. HPLC chromatogram of **2** after incubation in human plasma. 49
Figure 47. HPLC chromatogram of **3** after incubation in human plasma. 50
Figure 48. HPLC chromatogram of **4** after incubation in human plasma. 50
Figure 49. HPLC chromatogram of **5** after incubation in human plasma. 51
Figure 50. HPLC chromatogram of **6** after incubation in human plasma. 51
Figure 51. HPLC chromatogram of **7** after incubation in human plasma. 51
Figure 52. Change of the UV/Vis spectra of $[Ru(bipy)_3]Cl_2$ upon irradiation. 52
Figure 53. Change of the UV/Vis spectra of PpIX upon irradiation. 52
Figure 54. Change of the UV/Vis spectra of **1** upon irradiation. 53
Figure 55. Change of the UV/Vis spectra of **2** upon irradiation. 53
Figure 56. Change of the UV/Vis spectra of **3** upon irradiation. 54
Figure 57. Change of the UV/Vis spectra of **4** upon irradiation. 54

Figure 58. Change of the UV/Vis spectra of **5** upon irradiation. 55
Figure 59. Change of the UV/Vis spectra of **6** upon irradiation. 55
Figure 60. Change of the UV/Vis spectra of **7** upon irradiation. 56
Figure 61. Change of the NMR spectrum of **2** upon irradiation. 56
Table 7. Distribution coefficient for compounds **1**-**7**. 57
Figure 62. Time dependent cellular uptake of **1** in HeLa cells. 57
Figure 63. Time dependent cellular uptake of **2** in HeLa cells. 58
Figure 64. Time dependent cellular uptake of **3** in HeLa cells. 58
Figure 65. Time dependent cellular uptake of **4** in HeLa cells. 59
Figure 66. Time dependent cellular uptake of **5** in HeLa cells. 59
Figure 67. Time dependent cellular uptake of **6** in HeLa cells. 60
Figure 68. Time dependent cellular uptake of **7** in HeLa cells. 60
Figure 69. Comparison of uptake of **1**-**7** in HeLa cells. 61
Figure 70. Cell uptake mechanism study of **1**. 61
Figure 71. Cell uptake mechanism study of **2**. 62
Figure 72. Cell uptake mechanism study of **3**. 62
Figure 73. Cell uptake mechanism study of **4**. 63
Figure 74. Cell uptake mechanism study of **5**. 63
Figure 75. Cell uptake mechanism study of **6**. 64
Figure 76. Cell uptake mechanism study of **7**. 64
Figure 77. Confocal luminescence image of HeLa cells incubated with the compounds **1**-**7** (1P). 65
Figure 78. Confocal luminescence image of HeLa cells incubated with the compounds **1**-**7** (2P). 66
Figure 79. Cellular distribution of **1**-**7** in HeLa cells determined via ICP-MS. 67
Figure 80. Confocal luminescence image of HeLa cells incubated with 2′,7′-dichlorofluorescein diacetate and the compounds **1**-**7** (1P). 68
Figure 81. Confocal luminescence image of HeLa cells incubated with 2′,7′-dichlorofluorescein diacetate and the compounds **1**-**7** (2P). 69
Table 8. IC_{50} values in the dark and upon irradiation at 480 and 540 nm for **1-7** in RPE-1 and HeLa cells. 70
Table 9. IC_{50} values in the dark and upon irradiation at 480 and 540 nm for **1-7** in CT-26 and U373 cells. 71

Figure 82. Cell death mechanism study. 72

Figure 83. 1P and 2P Z-stack confocal laser scanning microscopy images in HeLa MCTS after incubation of **1** after 12 h. 72

Figure 84. 1P and 2P Z-stack confocal laser scanning microscopy images in HeLa MCTS after incubation of **2** after 12 h. 73

Figure 85. 1P and 2P Z-stack confocal laser scanning microscopy images in HeLa MCTS after incubation of **3** after 12 h. 73

Figure 86. 1P and 2P Z-stack confocal laser scanning microscopy images in HeLa MCTS after incubation of **4** after 12 h. 74

Figure 87. 1P and 2P Z-stack confocal laser scanning microscopy images in HeLa MCTS after incubation of **5** after 12 h. 74

Figure 88. 1P and 2P Z-stack confocal laser scanning microscopy images in HeLa MCTS after incubation of **6** after 12 h. 75

Figure 89. 1P and 2P Z-stack confocal laser scanning microscopy images in HeLa MCTS after incubation of **7** after 12 h. 75

Figure 90. 1P and 2P Z-stack confocal laser scanning microscopy images in HeLa MCTS after incubation of **1** after 60 h. 76

Figure 91. 1P and 2P Z-stack confocal laser scanning microscopy images in HeLa MCTS after incubation of **2** after 60 h. 76

Figure 92. 1P and 2P Z-stack confocal laser scanning microscopy images in HeLa MCTS after incubation of **3** after 60 h. 77

Figure 93. 1P and 2P Z-stack confocal laser scanning microscopy images in HeLa MCTS after incubation of **4** after 60 h. 77

Figure 94. 1P and 2P Z-stack confocal laser scanning microscopy images in HeLa MCTS after incubation of **5** after 60 h. 78

Figure 95. Representative image of the growth inhibition assay in HeLa MCTS treated in the dark. 79

Figure 96. Representative image of the growth inhibition assay in HeLa MCTS treated exposed 1P irradiation. 80

Figure 97. Representative image of the growth inhibition assay in HeLa MCTS treated exposed 2P irradiation. 81

Table 10. IC_{50} values in HeLa MCTS in the dark and upon 1P or 2P irradiation for **1-7**. 82

Figure 98. Time dependent biodistribution of **7** in a mouse model with a SW620/AD300 tumor. 82
Figure 99. The picture of tumor H&E stain slices. 83
Figure 100. The picture of organ H&E stain slices. 83

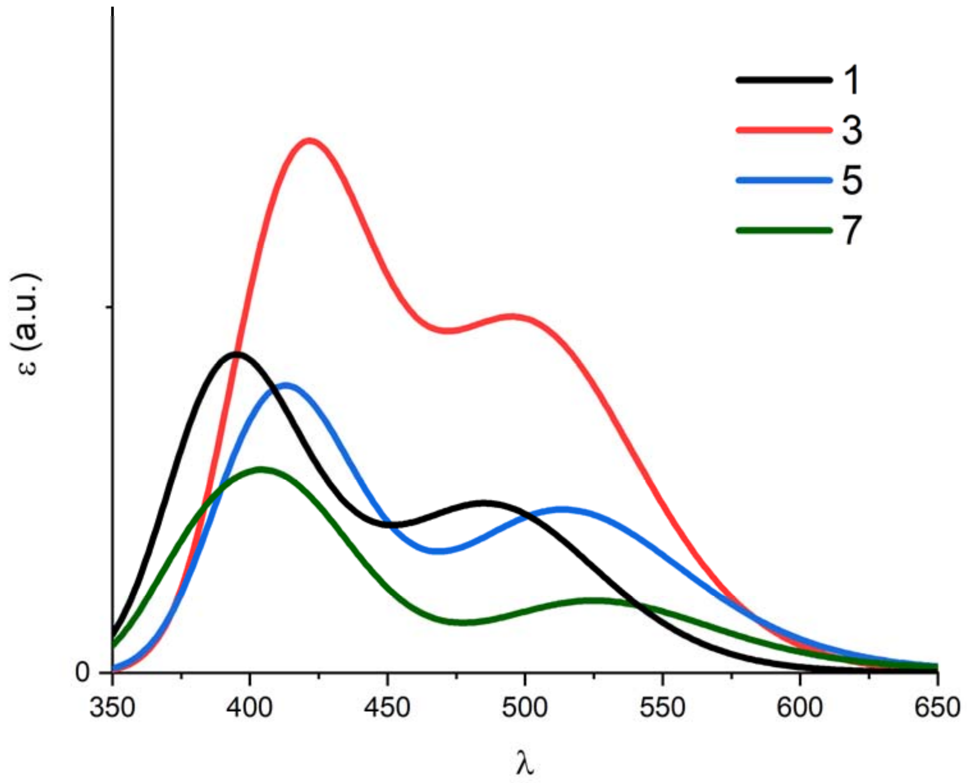

Figure 1. Simulated absorption spectra of compounds **1**, **3**, **5** and **7**.

Table 1. Theoretical data for **1**.

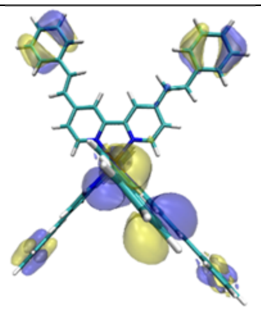 284	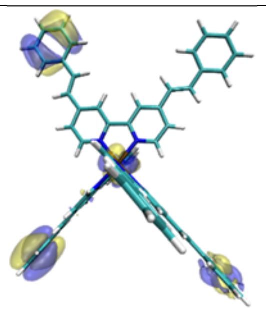 285	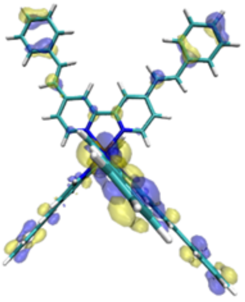 286

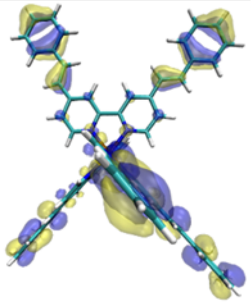

287

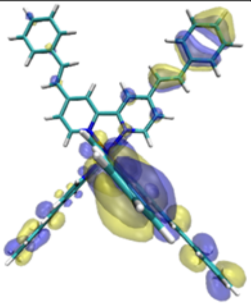

288

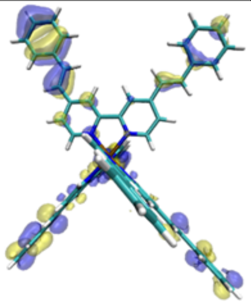

289

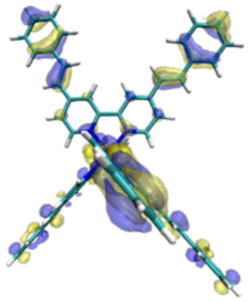

290

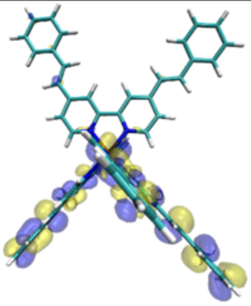

291

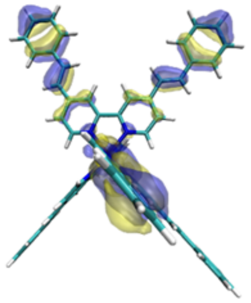

292 (HOMO)

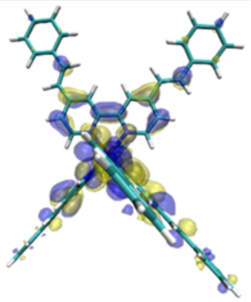

293 (LUMO)

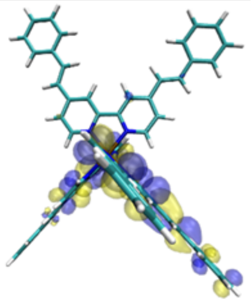

294

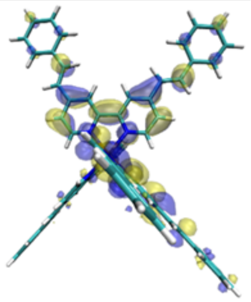

295

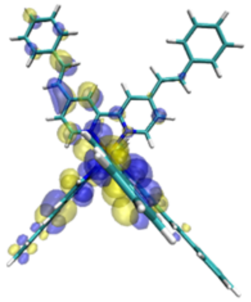

296

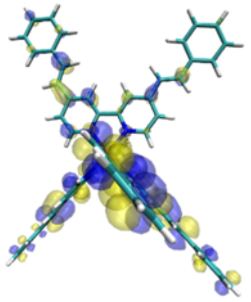

297

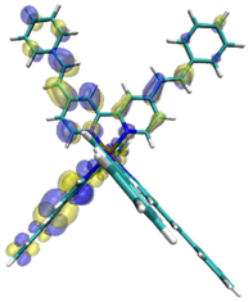

298

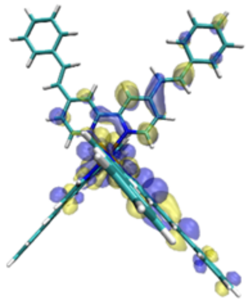

299

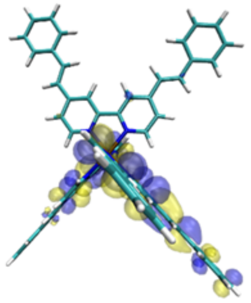

300

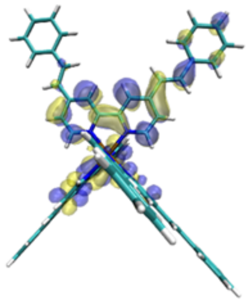

301

N states	E (nm)	f (a.u.)	Main contribution weight	Initial MO	Final MO	D_{CT} (Å)
1	510.42	0.0845	0.65464	290	293	0.438
2	505.21	0.0066	0.61305	290	294	2.471
3	505.03	0.0063	0.59814	290	295	2.32
4	502.2	0.1436	0.66059	292	293	2.869
5	501.79	0.1497	0.65222	291	293	2.44
6	498.29	0.0255	0.46693	292	294	0.401
7	486.37	0.4254	0.34868	291	294	1.028
8	486.19	0.4411	0.33803	291	295	0.971
9	456.49	0.01	0.44342	292	295	0.539
10	435.81	0.1449	0.67352	288	293	3.499
11	435.77	0.1491	0.67328	289	293	3.658
12	426.42	0.0545	0.48255	287	293	1.219
13	423.83	0.1987	0.44949	287	293	1.512
14	421.52	0.014	0.37096	288	295	0.116
15	421.36	0.0164	0.36836	288	294	0.634
16	416.54	0.0054	0.3784	289	295	0.314
17	413.49	0.0533	0.6338	287	294	4.283
18	413.32	0.0536	0.63597	287	295	4.688
19	403.62	0.0371	0.6433	286	293	0.253
20	401.28	0.2013	0.64189	292	296	2.88
21	401.11	0.1972	0.6409	291	296	3.306
22	399.44	0.0044	0.55382	290	296	1.781
23	396.43	0.0509	0.55104	286	294	2.597
24	396.34	0.0576	0.5396	286	295	2.491
25	393.29	0.2274	0.48401	290	297	2.718
26	393.07	0.2546	0.56272	290	298	2.489
27	392.45	0.4004	0.51363	292	298	2.048
28	391.76	0.1081	0.44566	291	298	1.993
29	391.55	0.1276	0.41726	291	297	2.065
30	388.53	0.2751	0.43654	291	298	1.375
31	380.73	0.0301	0.44261	279	293	3.431
32	380.56	0.0331	0.44262	278	293	3.173
33	378.87	0.1613	0.60909	292	299	2.779
34	378.71	0.1677	0.61099	291	299	2.804
35	377	0.0033	0.45261	290	299	0.64
36	374.26	0.0057	0.28909	279	294	0.214
37	371.87	0.0228	0.28816	279	295	0.207
38	371.84	0.024	0.29189	278	295	0.485
39	365.12	0.0161	0.33862	280	293	1.819
40	364.46	0.0108	0.37609	281	293	5.414

Table 2. Theoretical data for **3**.

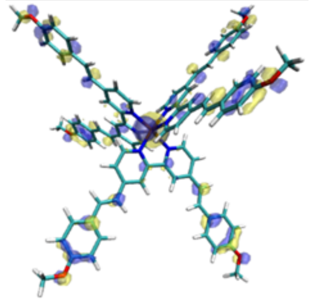 334	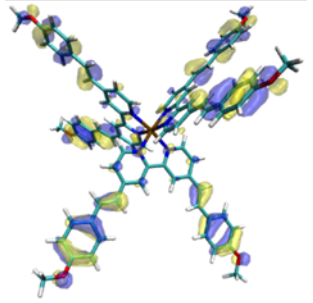 335	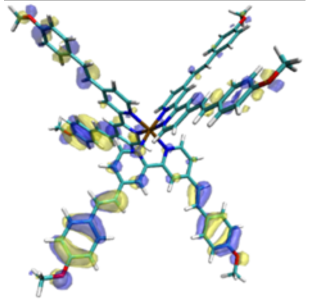 336
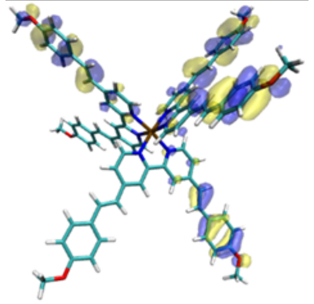 337	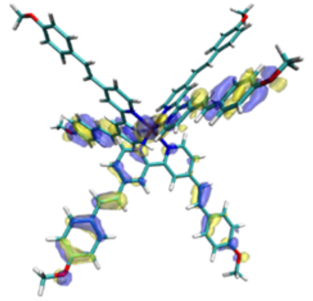 338	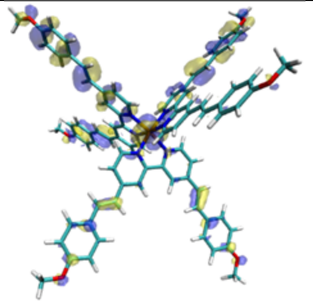 339
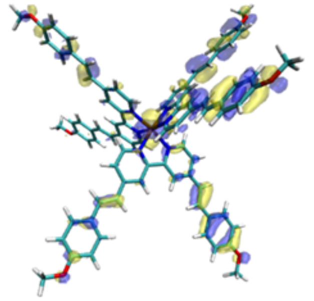 340 (HOMO)	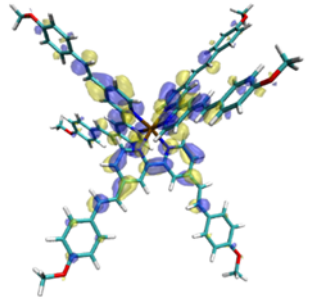 341 (LUMO)	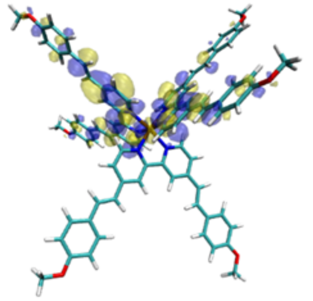 342
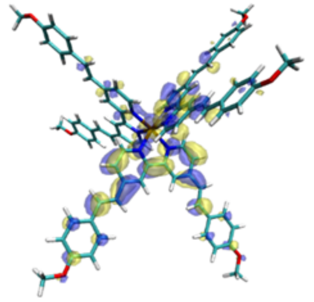 343	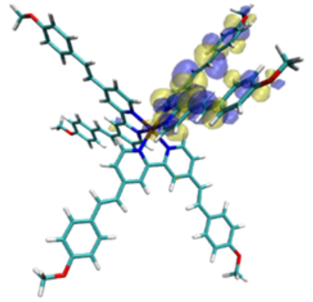 344	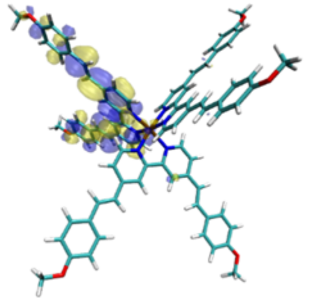 345
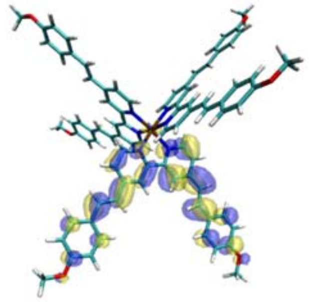 346		

N states	E (nm)	f (a.u.)	Main contribution weight	Initial MO	Final MO	D_{CT} (Å)	2PA (nm)	σ_2 (GM)
1	539.34	0.191	0.65277	340	341	5.045	1079	23.2
2	537.3	0.182	0.55645	338	341	2.123	1074	12.3
3	535.92	0.2311	0.59956	339	341	3.409	1074	31.8
4	528.12	0.0182	0.51345	340	342	5.216	1056	5.3
5	525.92	0.0538	0.49973	338	342	2.159	1051	5.3
6	525.06	0.038	0.55535	338	343	2.62	1051	11.3
7	519.01	0.3767	0.46164	339	342	0.356	1038	25.2
8	518.18	0.414	0.52857	339	343	2.807	1038	26.6
9	494.97	0.0291	0.39407	339	342	2.033	993	57.9
10	493.53	0.1519	0.60862	337	341	3.544	989	137.0
11	491.11	0.1617	0.6756	336	341	3.67	985	178.0
12	479.65	0.0755	0.4206	335	341	1.623	962	361.0
13	476.11	0.1214	0.50529	337	343	3.588	954	421.0
14	474.74	0.0291	0.48603	337	342	2.672	951	201.0
15	474.08	0.0575	0.53982	336	342	3.467	947	123.0
16	468.89	0.0506	0.45308	336	343	2.308	940	143.0
17	465.55	0.0644	0.55215	335	342	3.09	933	30.6
18	463.65	0.0823	0.66687	335	343	4.82	929	26.7
19	438.11	0.0206	0.65631	334	341	0.795	877	2.6
20	432.37	0.0134	0.62125	334	342	1.807	865	163.0
21	430.83	0.0111	0.62154	334	343	2.547	862	161.0
22	427.88	0.2267	0.63968	340	344	4.449	856	17.0
23	426.33	0.2596	0.64329	339	344	2.957	853	14.0
24	422.19	0.0373	0.55341	338	344	2.489	844	24.5
25	418.74	0.3769	0.52865	340	345	4.682	838	14.9
26	418	0.2808	0.50914	339	345	2.474		
27	416.44	0.3315	0.37951	339	346	3.212		
28	415.43	0.38	0.42787	338	345	5.258		
29	413.69	0.2762	0.51583	338	346	4.824		
30	412.53	0.1007	0.53337	339	346	5.507		

Table 3. Theoretical data for **5**.

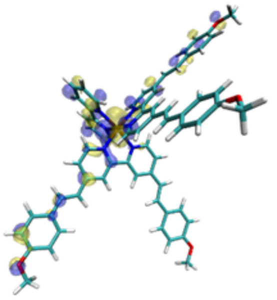 264	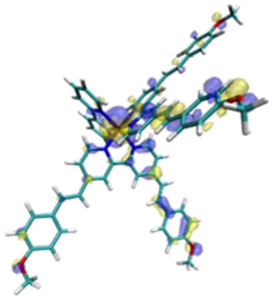 265	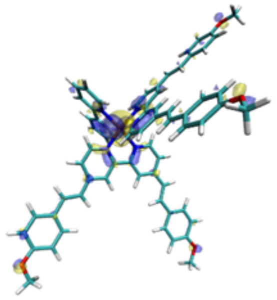 266
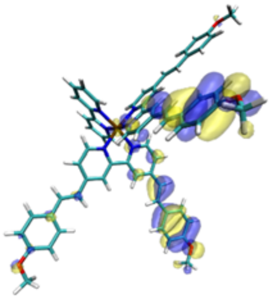 267	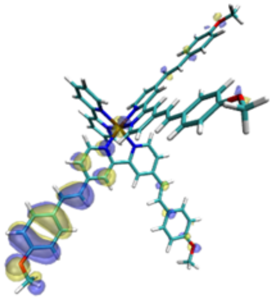 268	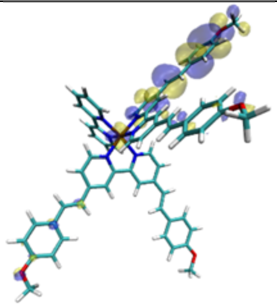 269
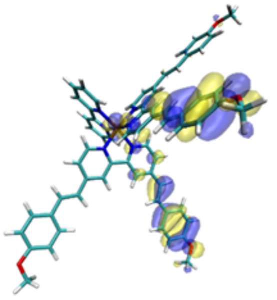 270 (HOMO)	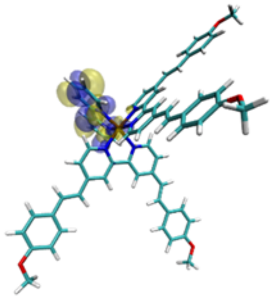 271 (LUMO)	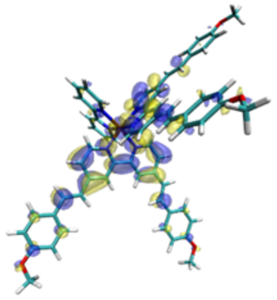 272
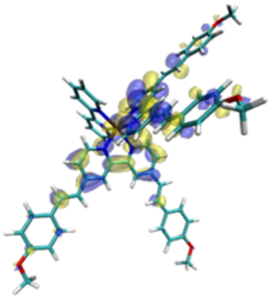 273	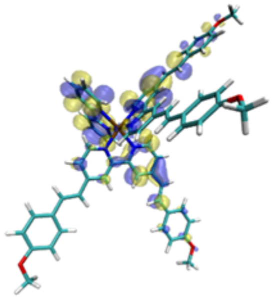 274	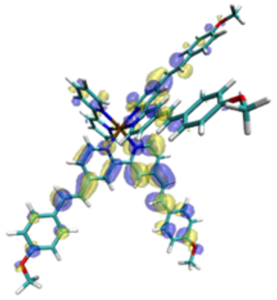 275
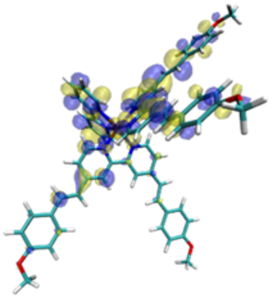 276		

N states	E (nm)	f (a.u.)	Main contribution weight	Initial MO	Final MO	D_{CT} (Å)	E 2PA (nm)	σ_2 (GM)
1	607.42	0.0082	0.66841	270	271	7.006	1216	4.8
2	595.91	0.0121	0.61297	269	271	4.486	1193	5.1
3	576.77	0.0106	0.62101	268	271	4.135	1154	45.9
4	552.19	0.012	0.6875	267	271	9.167	1103	30.7
5	535.78	0.2576	0.68462	270	272	4.319	1074	40.2
6	523.15	0.3737	0.62941	269	272	5.295	1047	41.4
7	520.01	0.152	0.54014	270	273	3.618	1043	118.0
8	511.52	0.1034	0.53896	269	273	6.67	1025	10.1
9	508.24	0.0131	0.49565	268	272	2.858	1017	162.0
10	503.58	0.1556	0.63404	268	273	7.426	1009	20.9
11	495.85	0.1294	0.46214	267	272	4.698	992	244.0
12	490.98	0.1845	0.54086	267	273	5.822	981	254.0
13	484.68	0.0018	0.64309	266	271	3.887	969	2.6
14	447.55	0.0073	0.63659	265	271	5.259	896	4.9
15	435.01	0.1606	0.55967	264	271	3.095	871	125.0
16	433.23	0.0886	0.59507	270	274	5.21	868	32.7
17	430.74	0.0078	0.62279	266	272	3.071	862	41.9
18	428.07	0.0132	0.5699	266	273	3.399	856	46.6
19	426.86	0.2059	0.62165	269	274	5.099	856	61.8
20	422.41	0.066	0.59824	268	274	5.615	847	52.0
21	418.04	0.4223	0.60359	270	275	4.335	835	14.2
22	414.55	0.0104	0.48704	267	274	6.416	830	25.1
23	411.19	0.3046	0.46562	270	276	3.806	824	13
24	410.77	0.4444	0.52518	269	275	2.564	823	10.8
25	406.32	0.1864	0.44272	268	275	5.094	814	2.6
26	404.94	0.0082	0.47796	264	273	3.957		
27	403.06	0.1056	0.43073	267	275	3.069		
28	402.11	0.085	0.41385	264	272	0.892		
29	398.56	0.1514	0.57807	268	276	11.02		
30	396.95	0.2333	0.42106	267	276	4.57		

Table 4. Theoretical data for **7**.

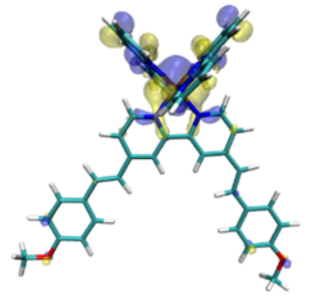<br>194	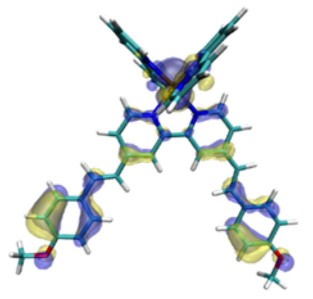<br>195	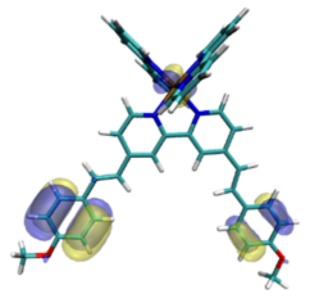<br>196
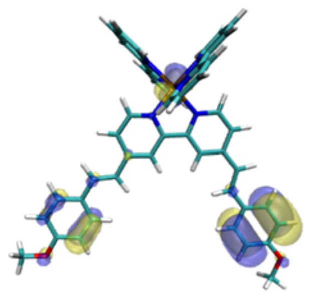<br>197	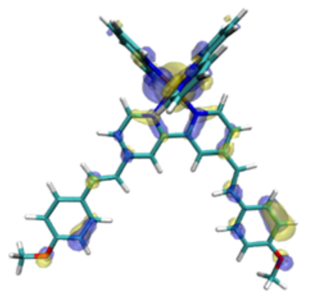<br>198	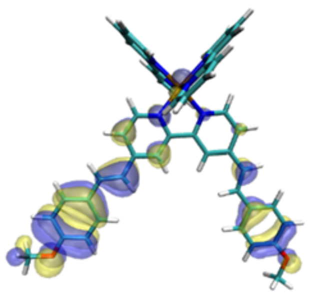<br>199
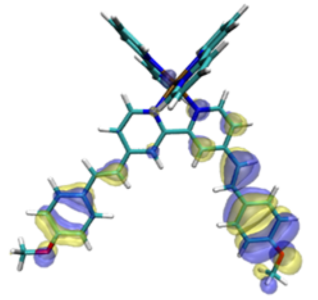<br>200 (HOMO)	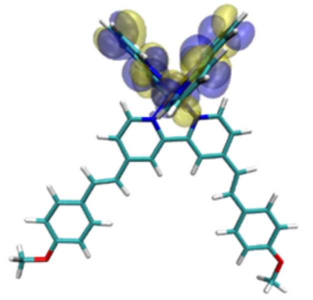<br>201 (LUMO)	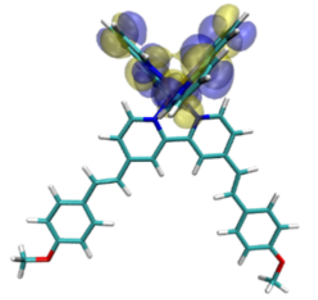<br>202
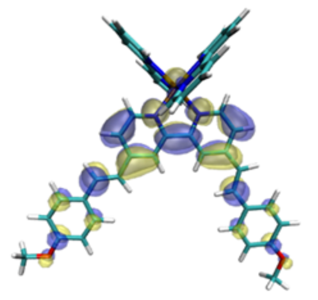<br>203	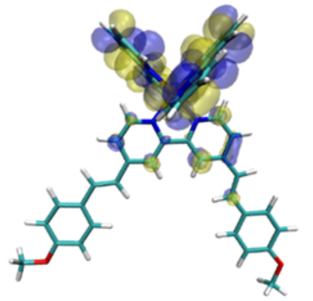<br>204	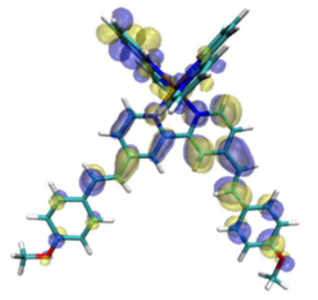<br>205
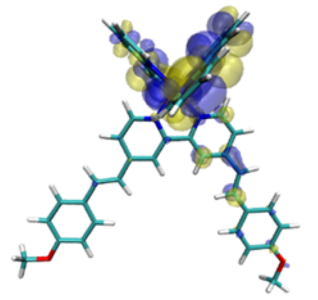<br>206	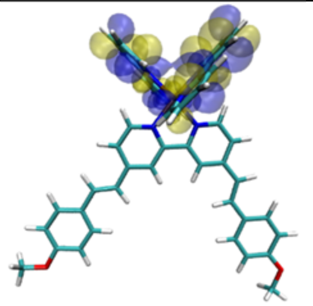<br>207	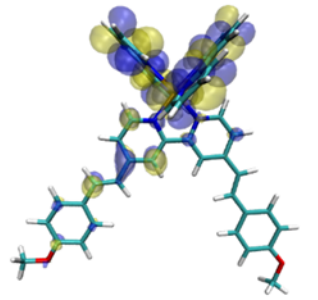<br>208

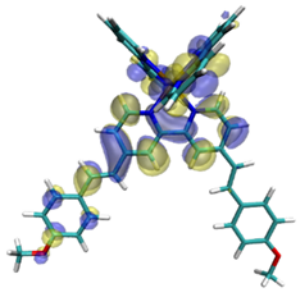 209		

N states	E (nm)	f (a.u.)	Main contribution weight	Initial MO	Final MO	D_{CT} (Å)	2PA (nm)	σ_2 (GM)
1	645.2	0.0072	0.6787	200	201	9.55	1292.	18.60
2	640.52	0.0115	0.67336	199	201	9.198	1279.	26.30
3	623.35	0.0011	0.63714	200	202	9.749	1246.	2.080
4	617.3	0.001	0.63241	199	202	9.89	1234.	2.150
5	538.1	0.2304	0.69288	200	203	6.213	1078.	148.0
6	520.33	0.3431	0.68093	199	203	5.945	1042.	278.0
7	477.35	0.0037	0.61054	198	201	2.843	954.3	0.353
8	475.12	0.0013	0.61373	198	202	2.955	950.7	1.240
9	454.63	0.0762	0.66405	200	204	9.452	908.9	28.80
10	452.88	0.0135	0.6587	199	204	9.068	905.6	13.20
11	446.21	0.0059	0.43818	194	201	2.946	892.5	3.040
12	442.68	0.0001	0.52639	195	201	3.473	886.2	28.50
13	426.76	0.0877	0.51484	194	201	2.422	855.6	18.70
14	419.91	0.1879	0.69227	199	205	7.348	841.1	6.590
15	417.85	0.5569	0.53545	200	205	4.89	835.4	8.550
16	415.52	0.0387	0.60438	200	206	7.875	832.6	4.800
17	411.7	0.0531	0.67015	199	206	8.461	824.3	4.880
18	408.52	0.0551	0.36165	198	203	4.107	818.9	23.10
19	406.68	0.0299	0.52521	198	203	1.032	813.5	55.20
20	404.27	0.1411	0.41276	199	208	7.089	808.2	14.80
21	401.37	0.0321	0.46582	199	207	8.741	803.0	1.810
22	400.73	0.0784	0.48849	200	207	9.154	803.0	3.97
23	396.76	0.0912	0.3846	199	207	9.569	795.3	11.2
24	392.8	0.0164	0.65246	200	209	8.189	785.2	15.0
25	384.93	0.0098	0.57857	197	201	10.975	770.6	0.43
26	383.77	0.0023	0.66852	194	203	3.471		
27	382.96	0.015	0.59281	196	201	10.729		
28	379.99	0.7208	0.53637	199	209	5.601		
29	375.6	0.0015	0.58588	197	202	11.306		
30	372.81	0.0003	0.59467	196	202	11.498		

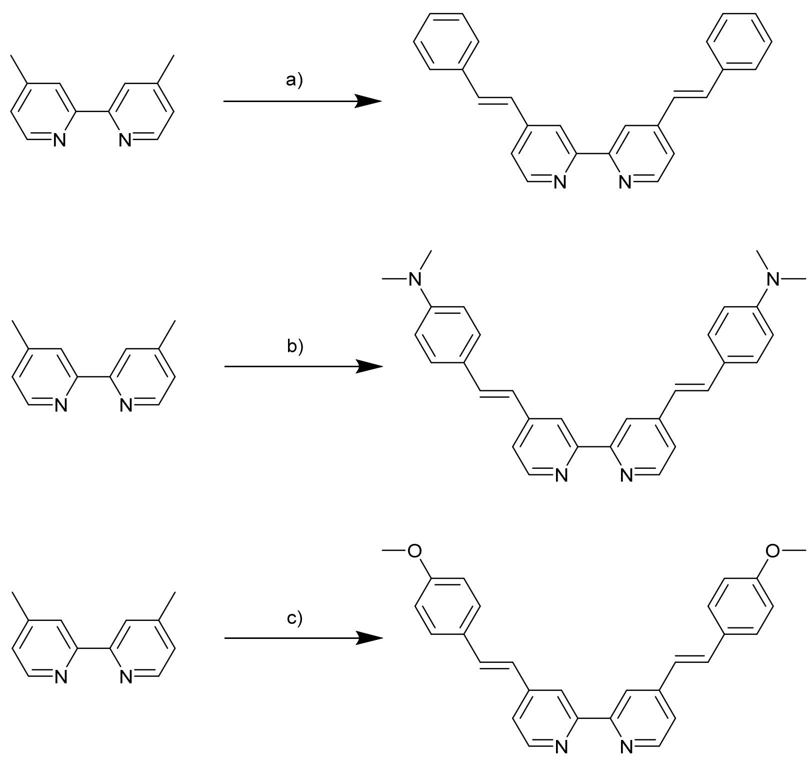

Figure 2. Synthesis of the (*E*,*E*')-4,4′-bisstyryl-2,2′-bipyridine based ligands. a) Benzaldehyde, potassium *tert*-butoxide, DMF, RT, 24 h, nitrogen atmosphere b) 4-(Dimethylamino)benzaldehyde, potassium *tert*-butoxide, DMF, 90°C, 19 h, nitrogen atmosphere, c) 4-Methoxybenzaldehyde, potassium *tert*-butoxide, DMF, RT, 24 h, nitrogen atmosphere.

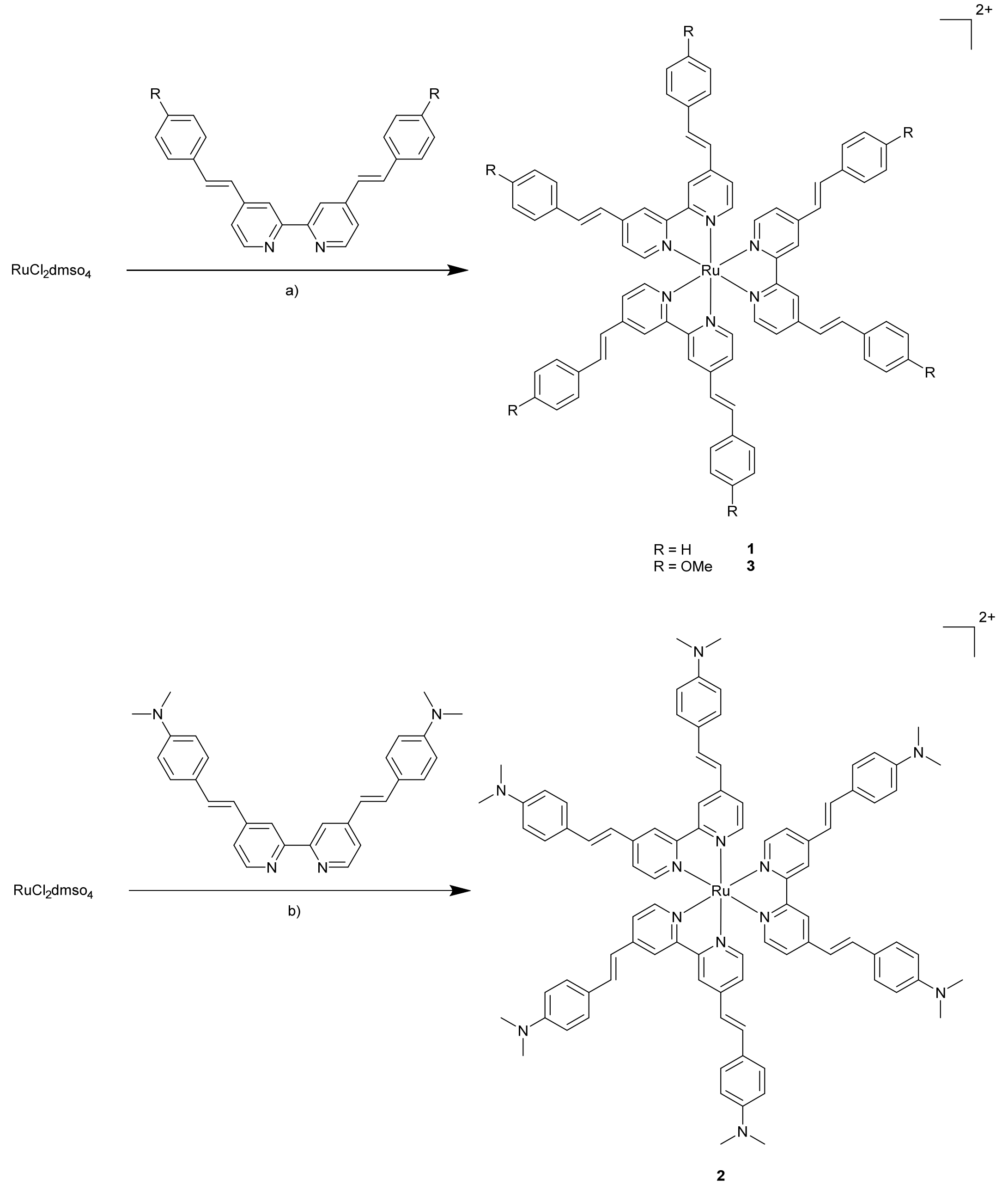

Figure 3. Synthesis of **1-3**. a) EtOH, reflux, 15-24 h, nitrogen atmosphere, b) LiCl, DMF, reflux, 48 h, nitrogen atmosphere.

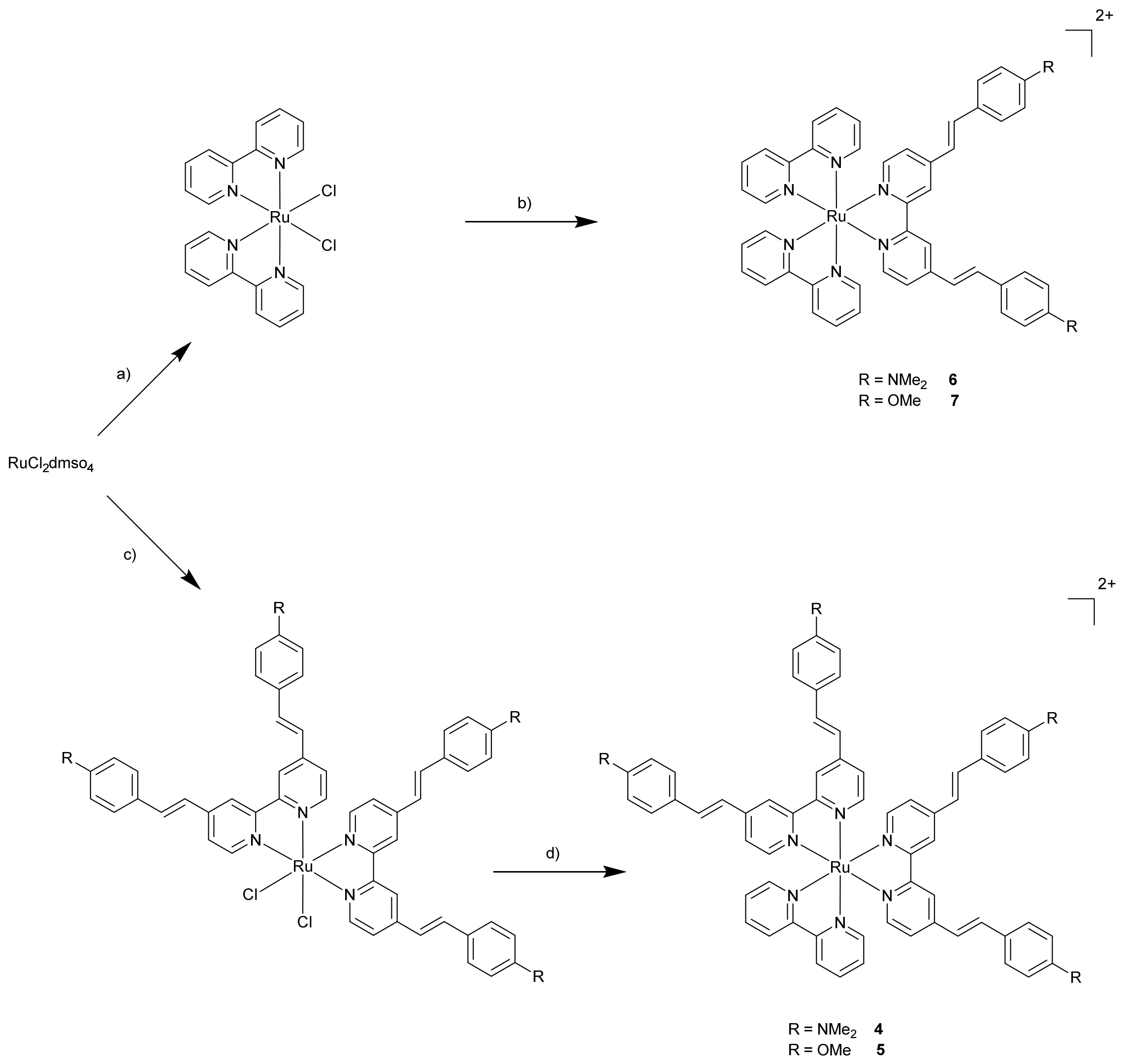

Figure 4. Synthesis of **4-7**. a) 2,2´-Bipyrdine, LiCl, DMF, reflux, 4h, nitrogen atmosphere, b) (*E*,*E*')-4,4´-Bisstyryl-2,2´-bipyridine derivative, EtOH, 6 h, nitrogen atmosphere, c) (*E*,*E*')-4,4´-Bisstyryl-2,2´-bipyridine derivative, LiCl, DMF, reflux, 4-6h, nitrogen atmosphere, d) 2,2´-Bipyrdine, EtOH, 6-7 h, nitrogen atmosphere.

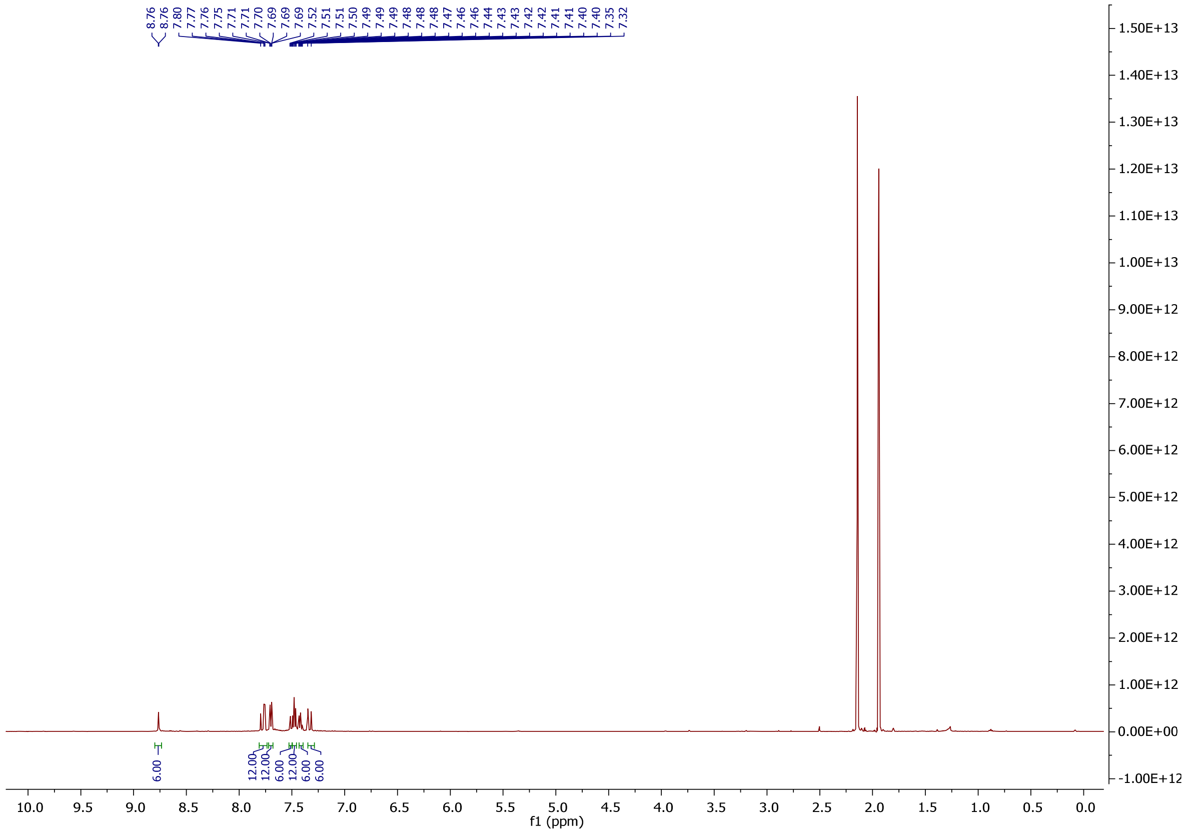

Figure 5. ^{1}H NMR spectrum of **1** in CD_3CN, 500 MHz.

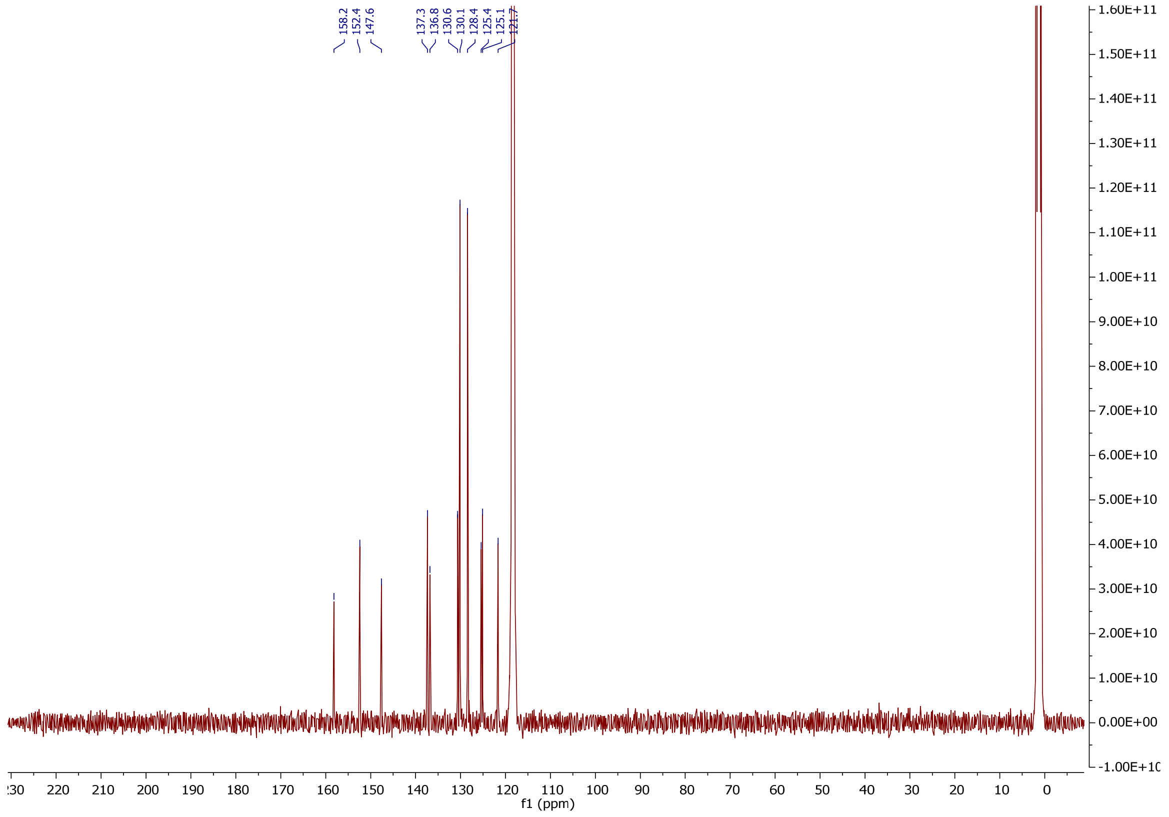

Figure 6. ^{13}C NMR spectrum of **1** in CD_3CN, 125 MHz.

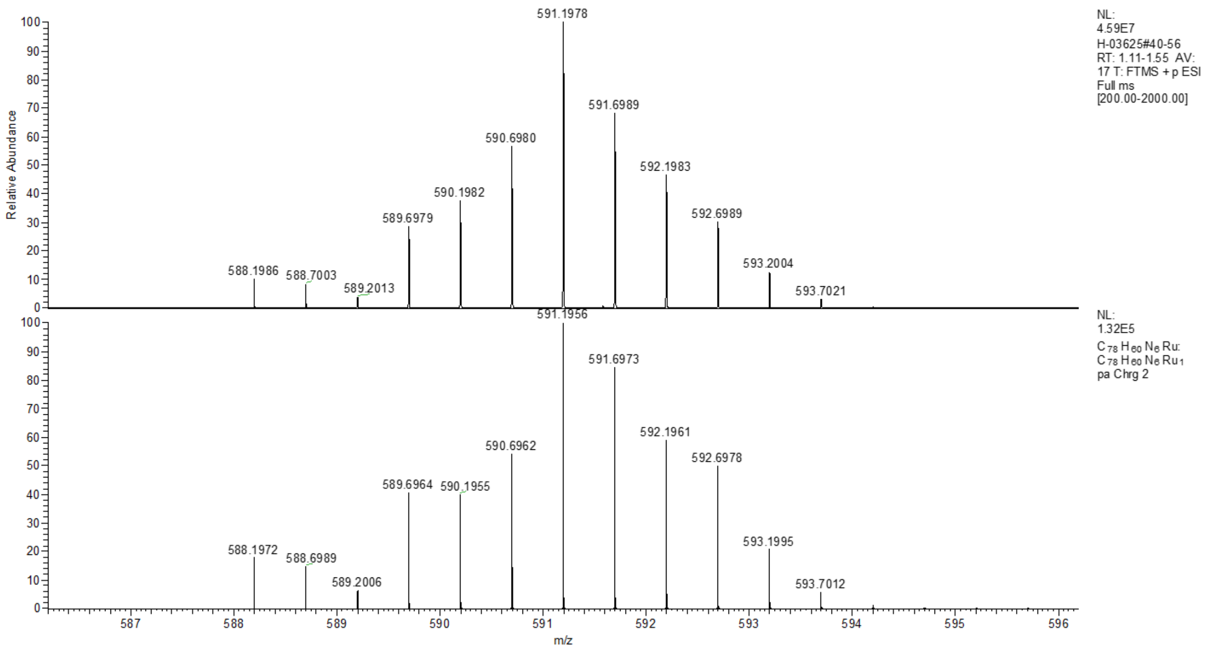

Figure 7. ESI-HRMS spectrum of **1** (positive detection mode).

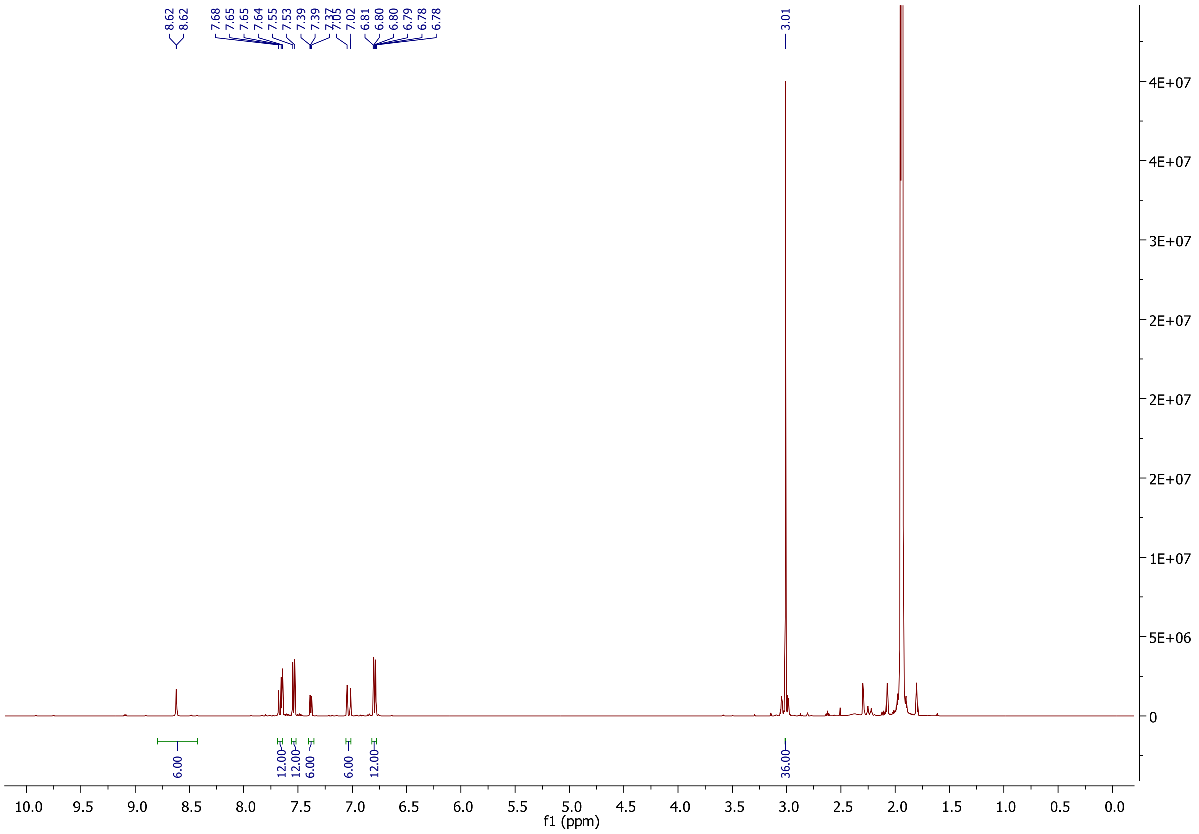

Figure 8. ^{1}H NMR spectrum of **2** in CD_3CN, 500 MHz.

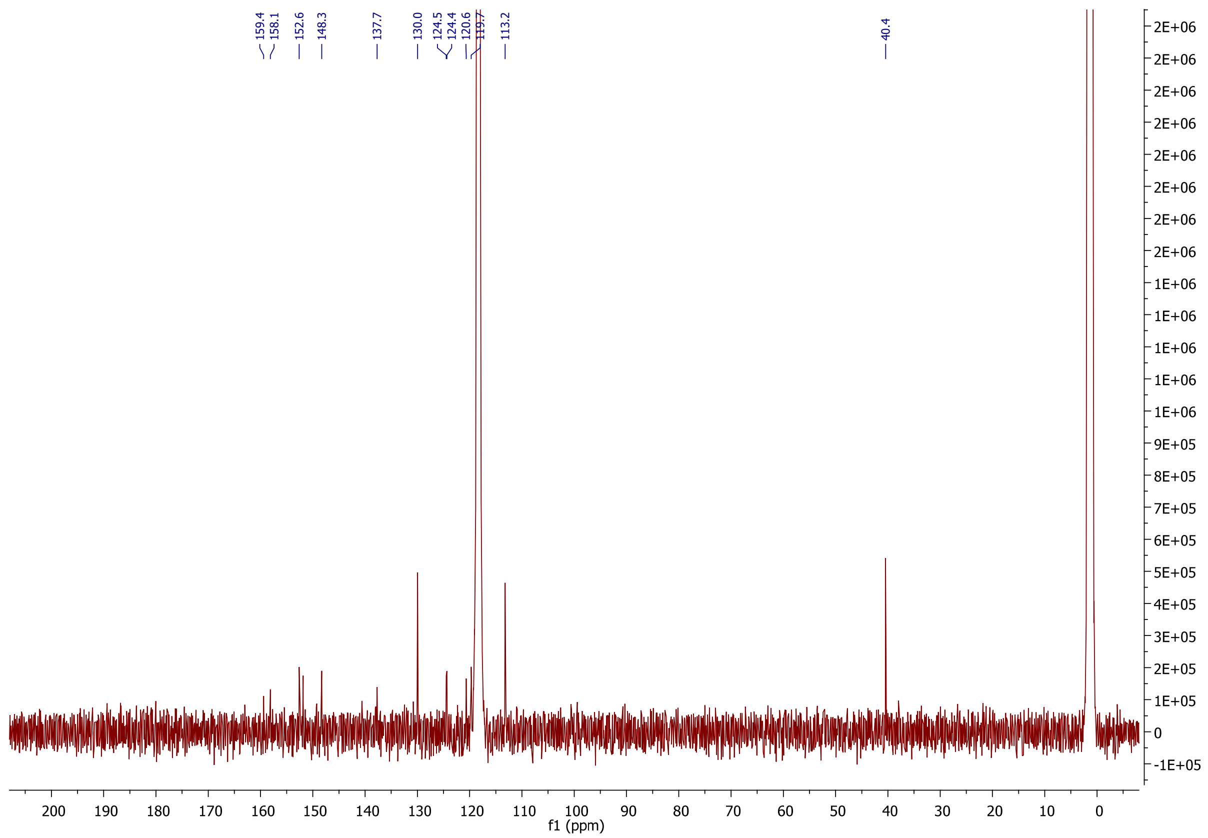

Figure 9. ^{13}C NMR spectrum of **2** in CD_3CN, 125 MHz.

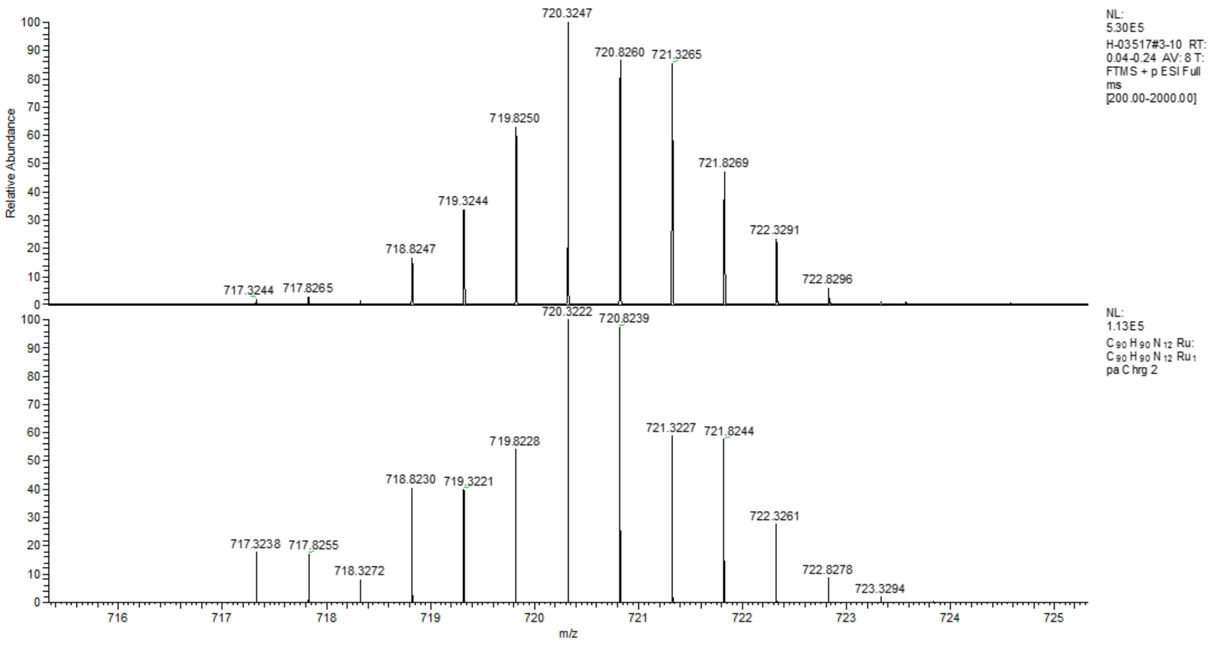

Figure 10. ESI-HRMS spectrum of **2** (positive detection mode).

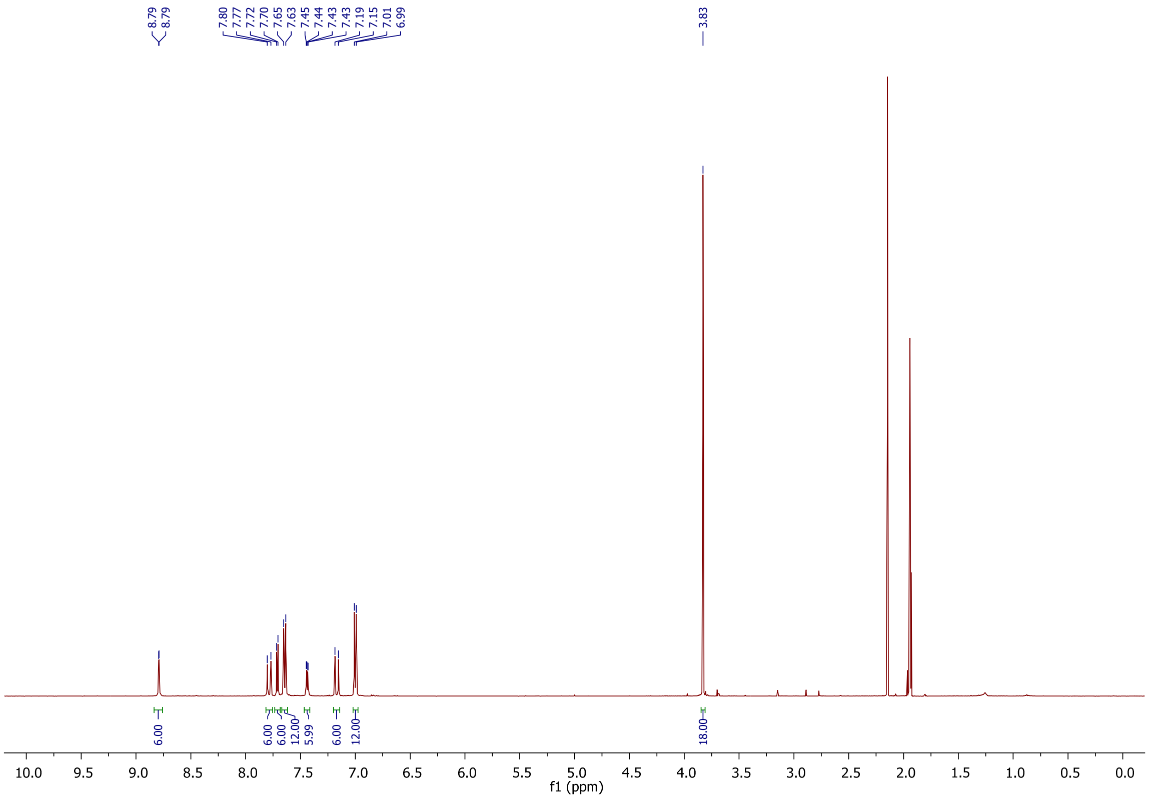

Figure 11. ^{1}H NMR spectrum of **3** in CD_3CN, 500 MHz.

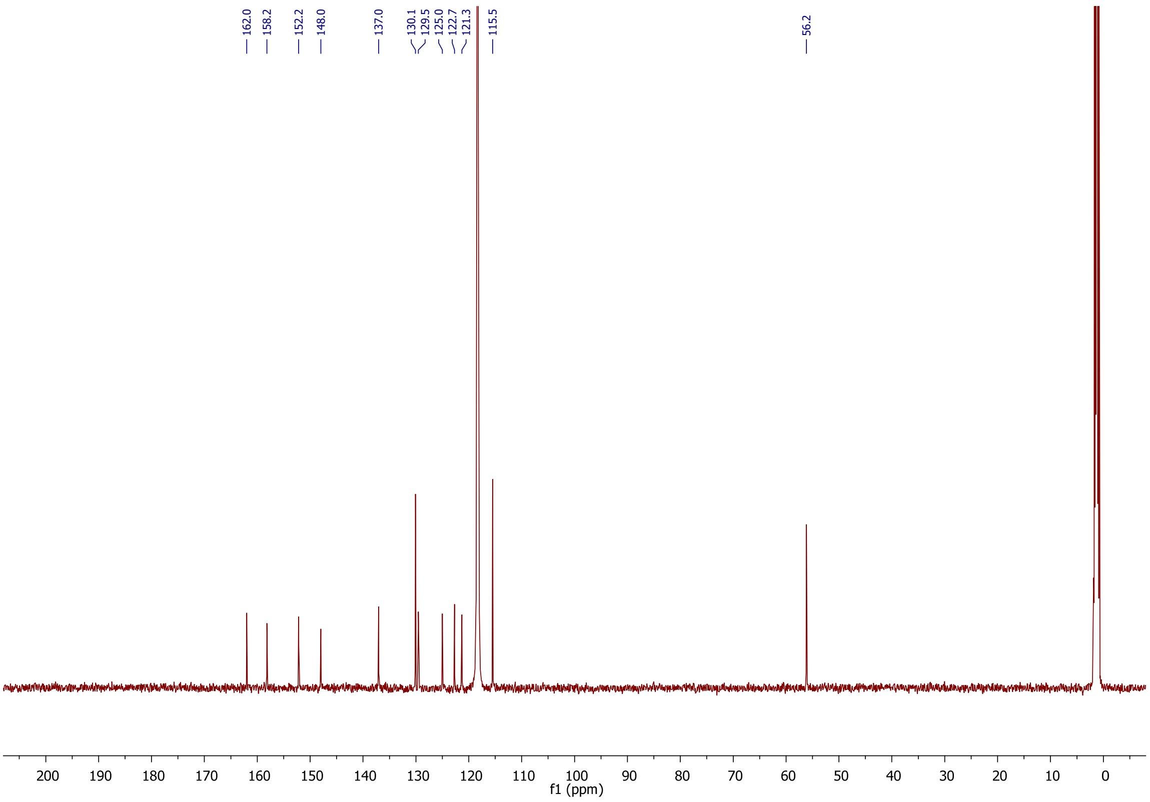

Figure 12. ^{13}C NMR spectrum of **3** in CD_3CN, 125 MHz.

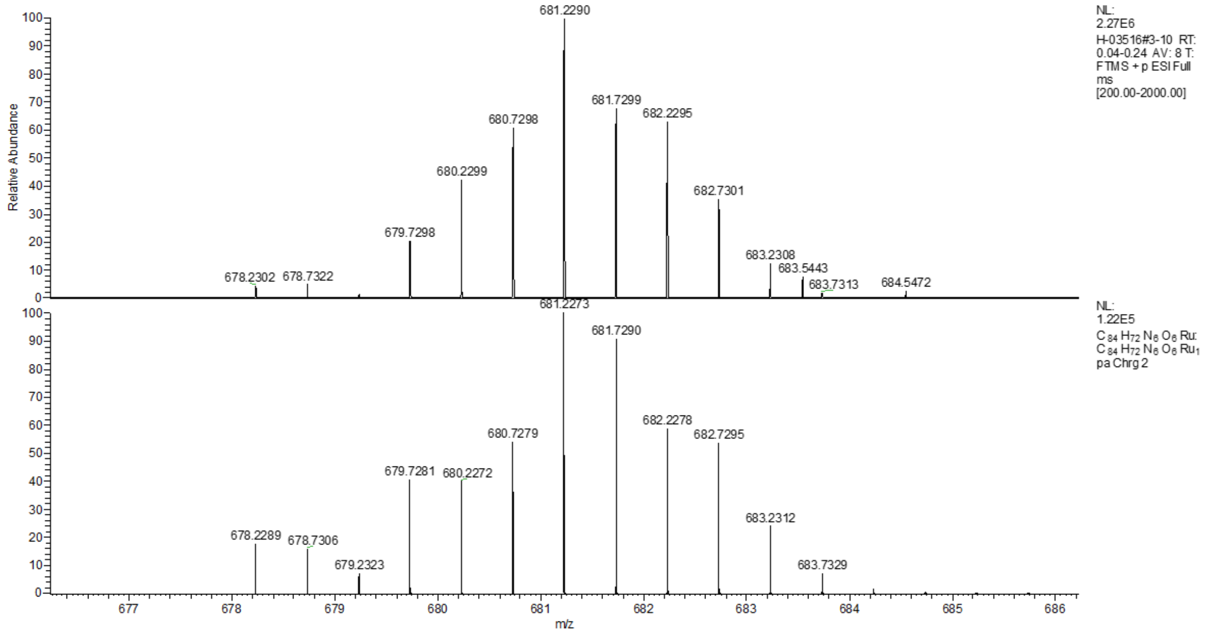

Figure 13. ESI-HRMS spectrum of **3** (positive detection mode).

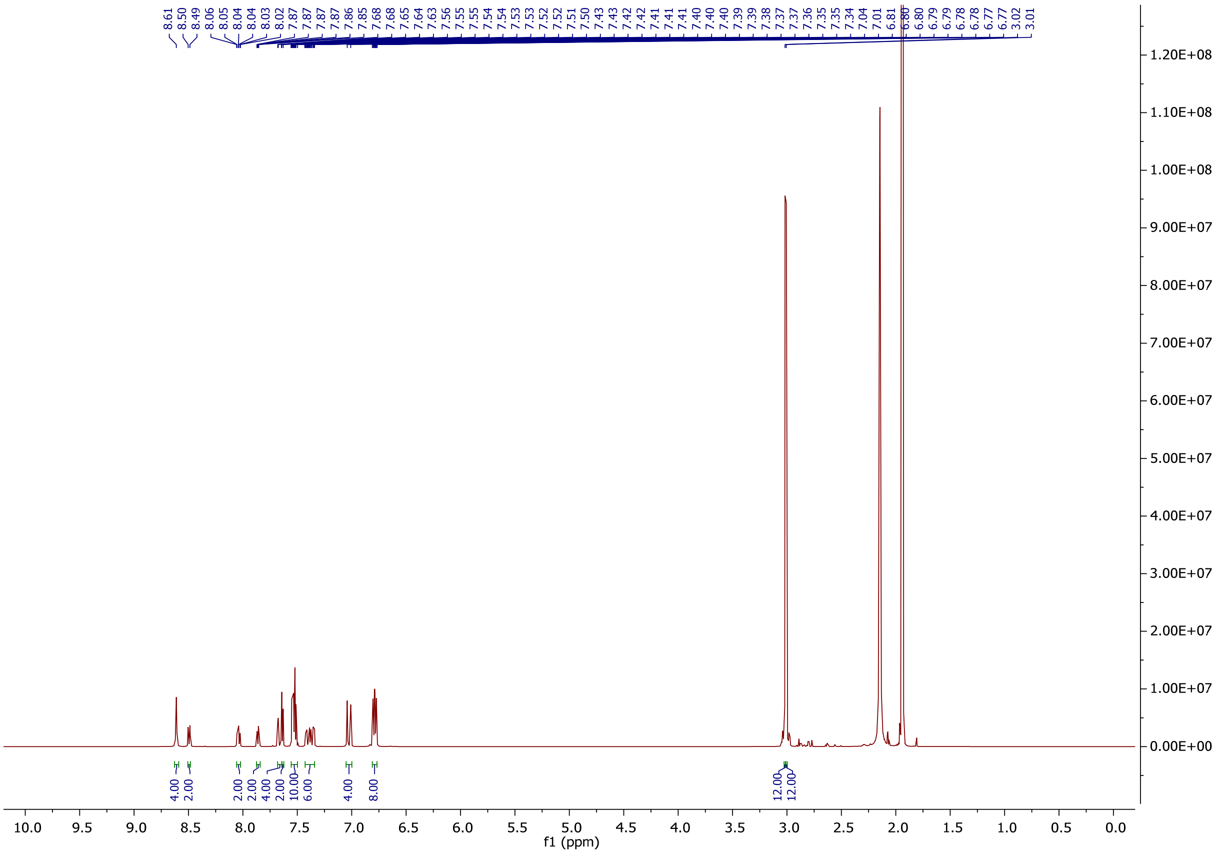

Figure 14. ^{1}H NMR spectrum of **4** in CD_3CN, 400 MHz.

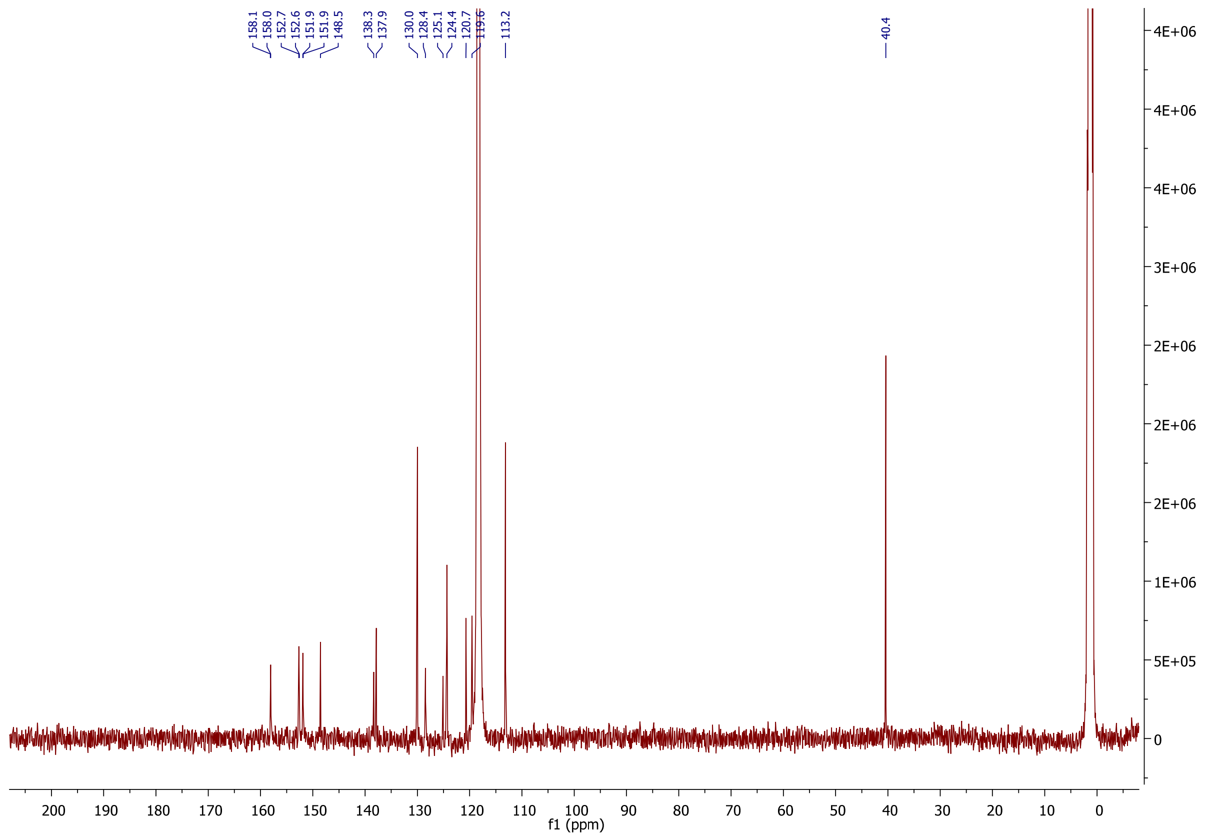

Figure 15. ^{13}C NMR spectrum of **4** in CD_3CN, 100 MHz.

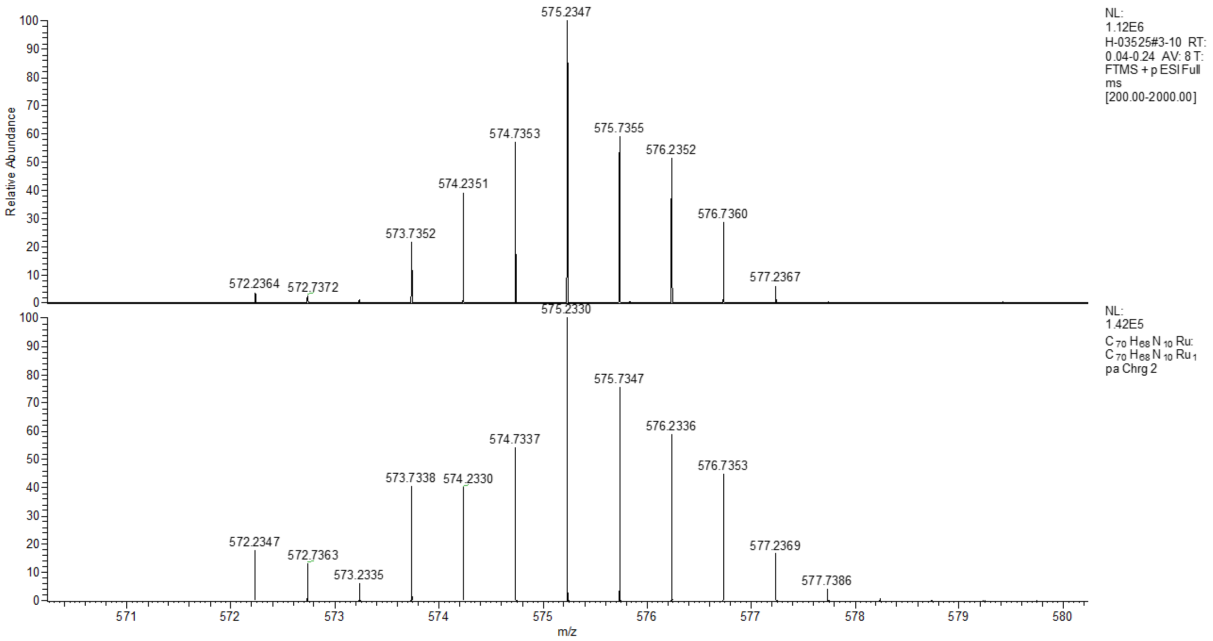

Figure 16. ESI-HRMS spectrum of **4** (positive detection mode).

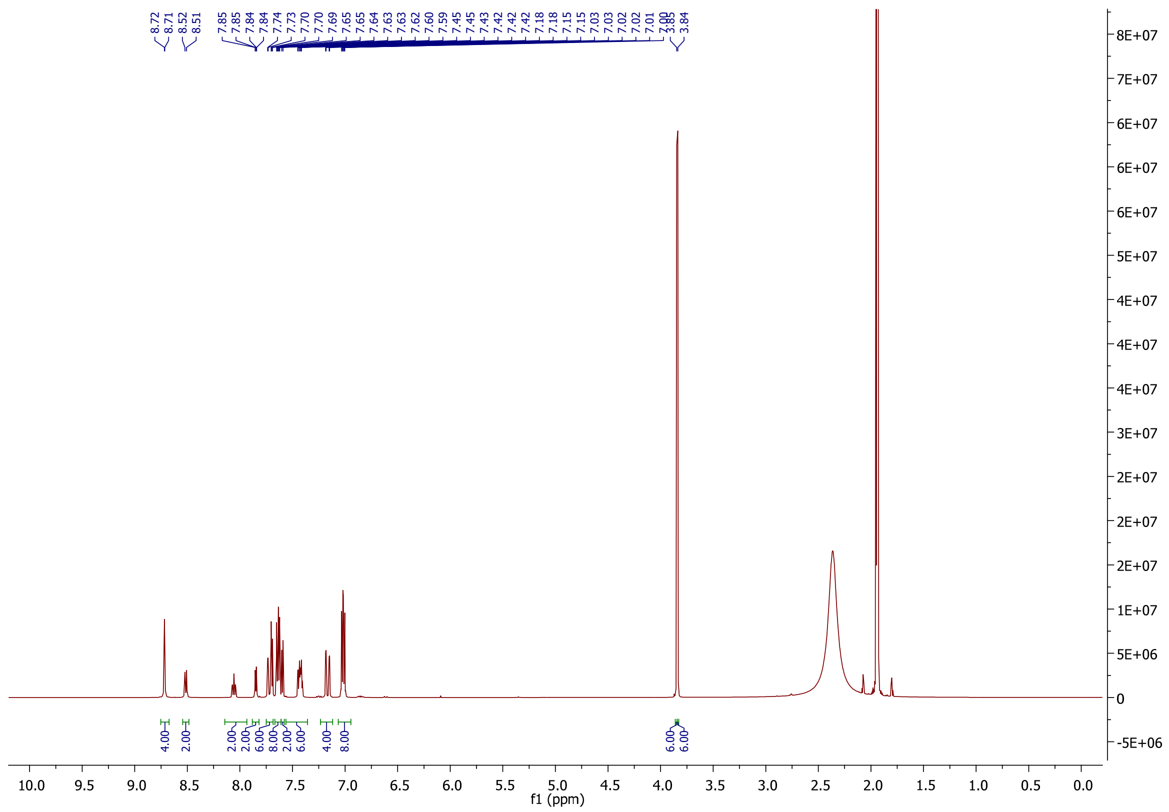

Figure 17. ^{1}H NMR spectrum of **5** in CD_3CN, 400 MHz.

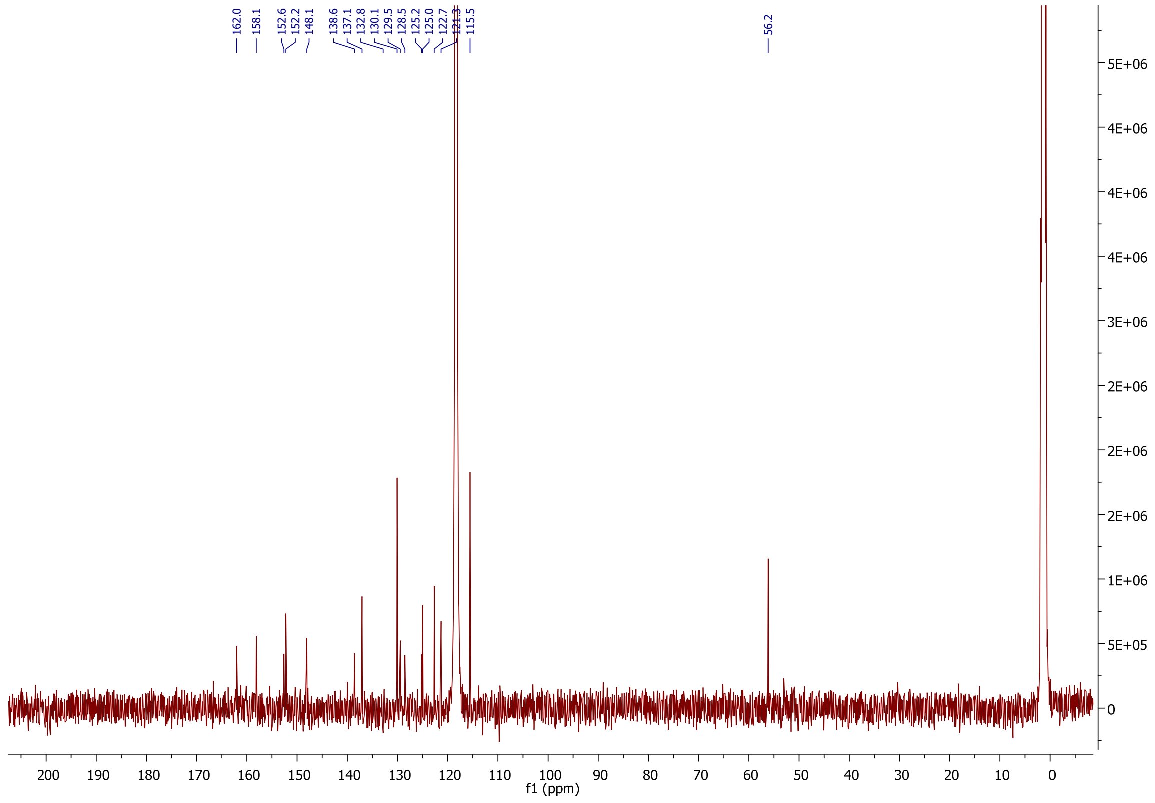

Figure 18. ^{13}C NMR spectrum of **5** in CD_3CN, 100 MHz.

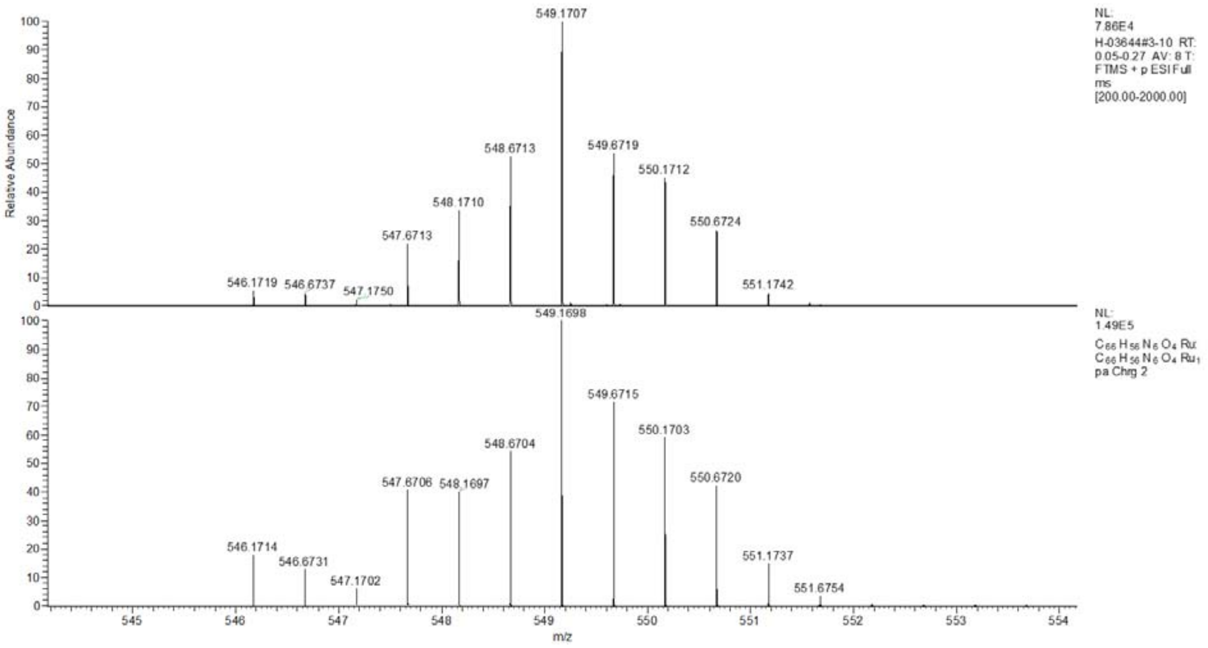

Figure 19. ESI-HRMS spectrum of **5** (positive detection mode).

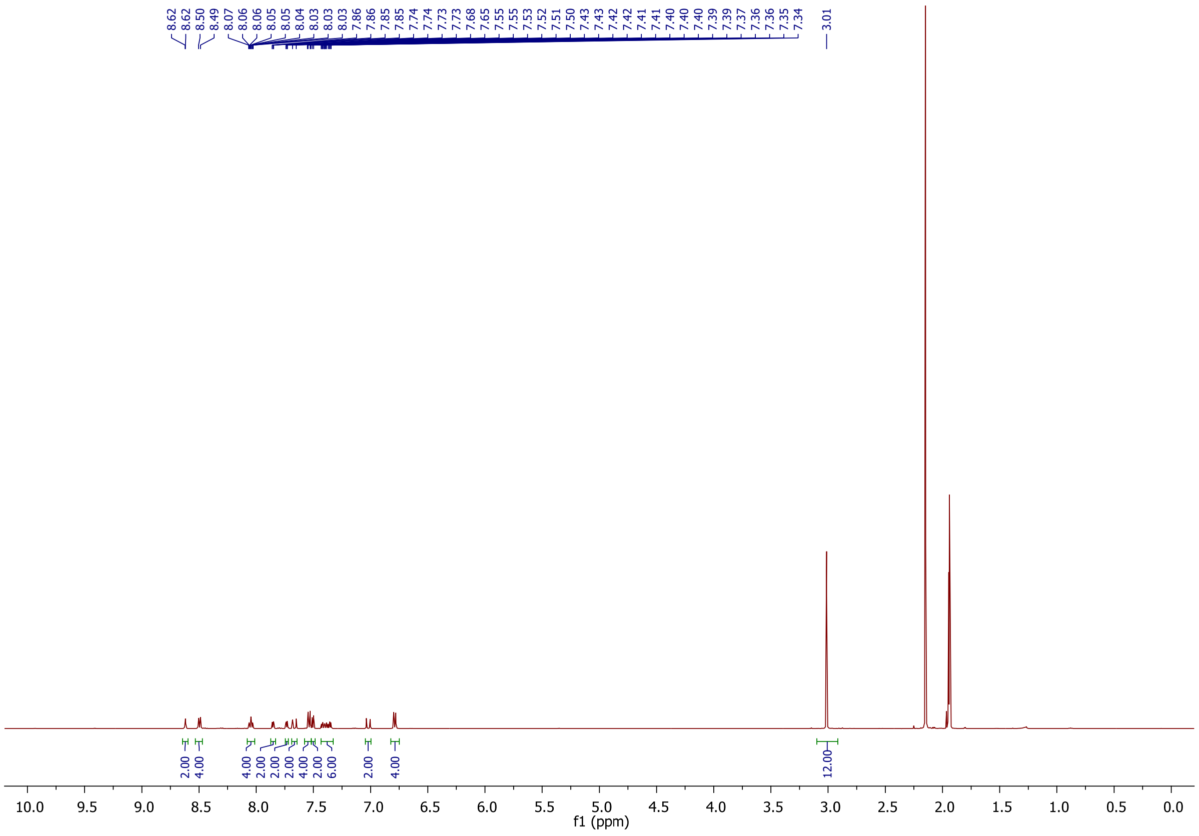

Figure 20. ^{1}H NMR spectrum of **6** in CD_3CN, 500 MHz.

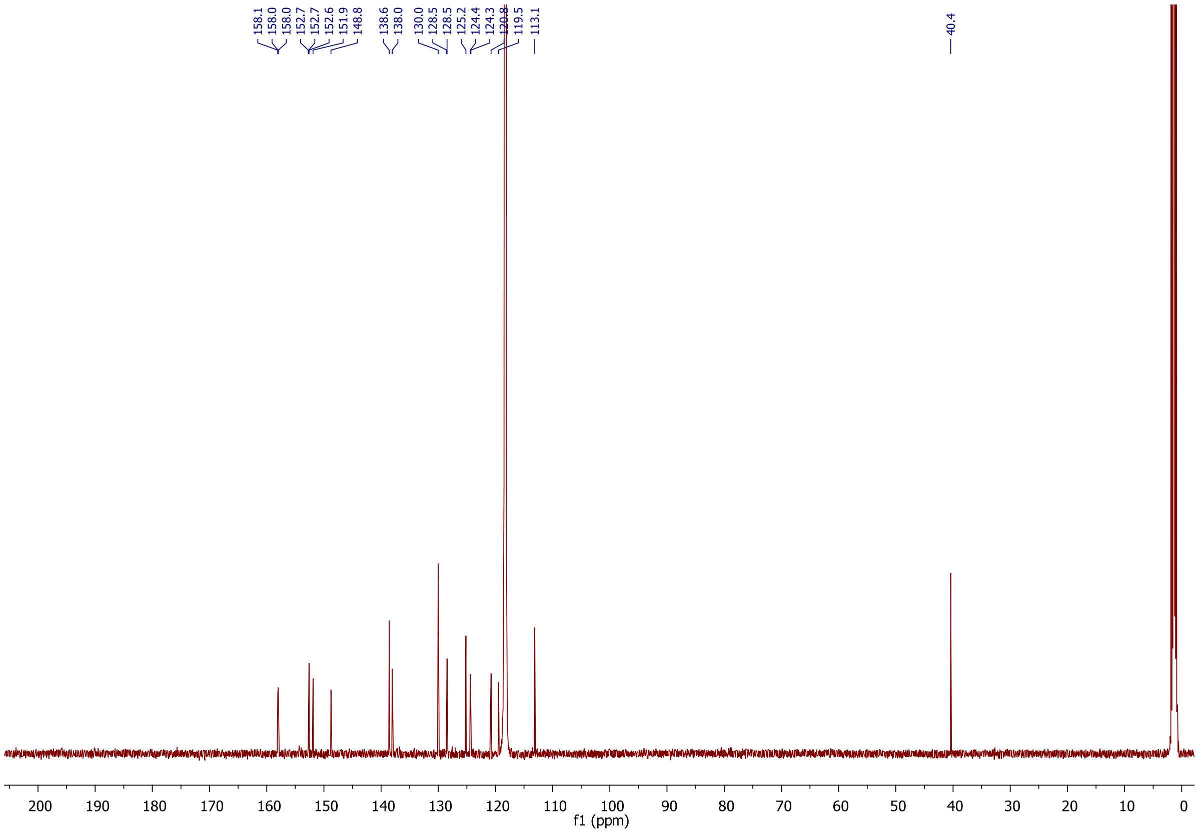

Figure 21. ^{13}C NMR spectrum of **6** in CD_3CN, 125 MHz.

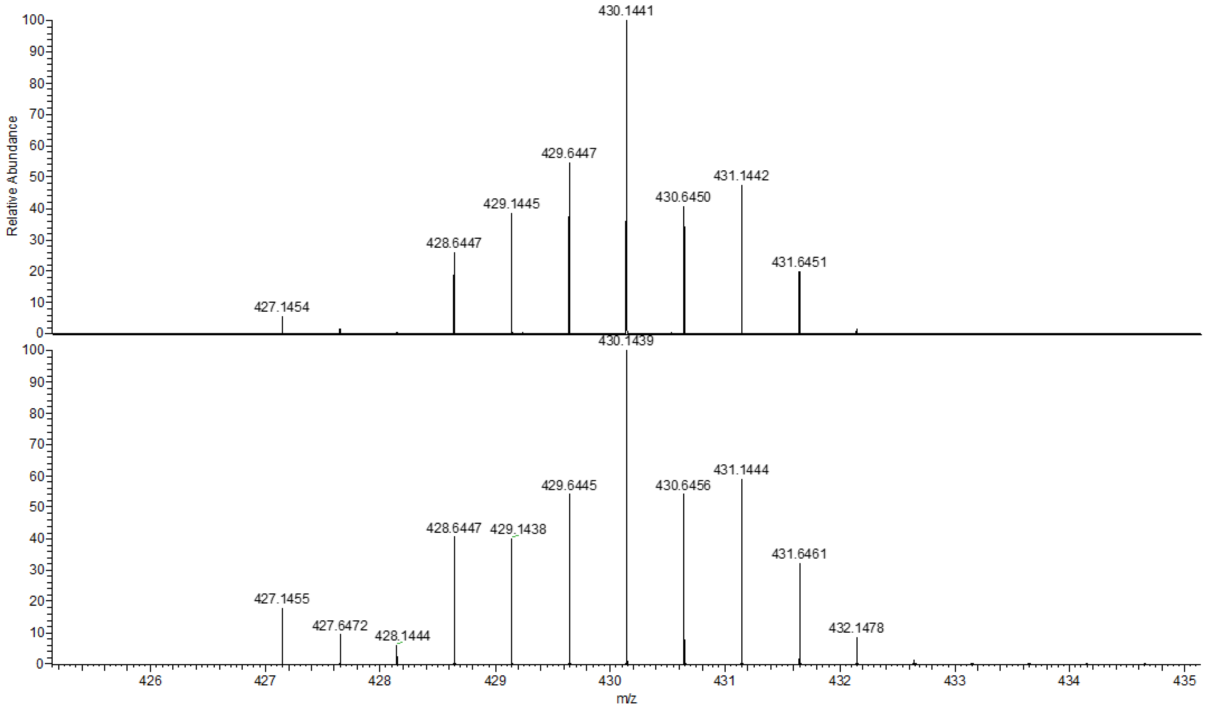

Figure 22. ESI-HRMS spectrum of **6** (positive detection mode).

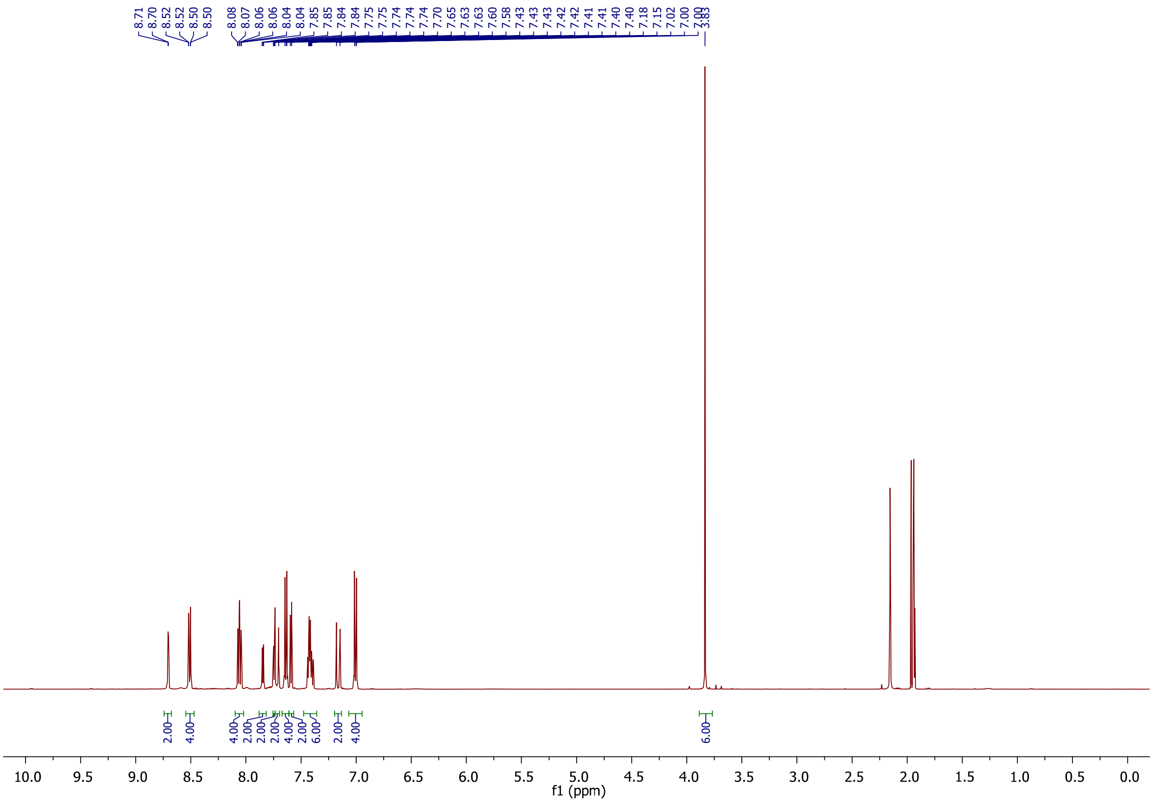

Figure 23. ^{1}H NMR spectrum of **7** in CD_3CN, 500 MHz.

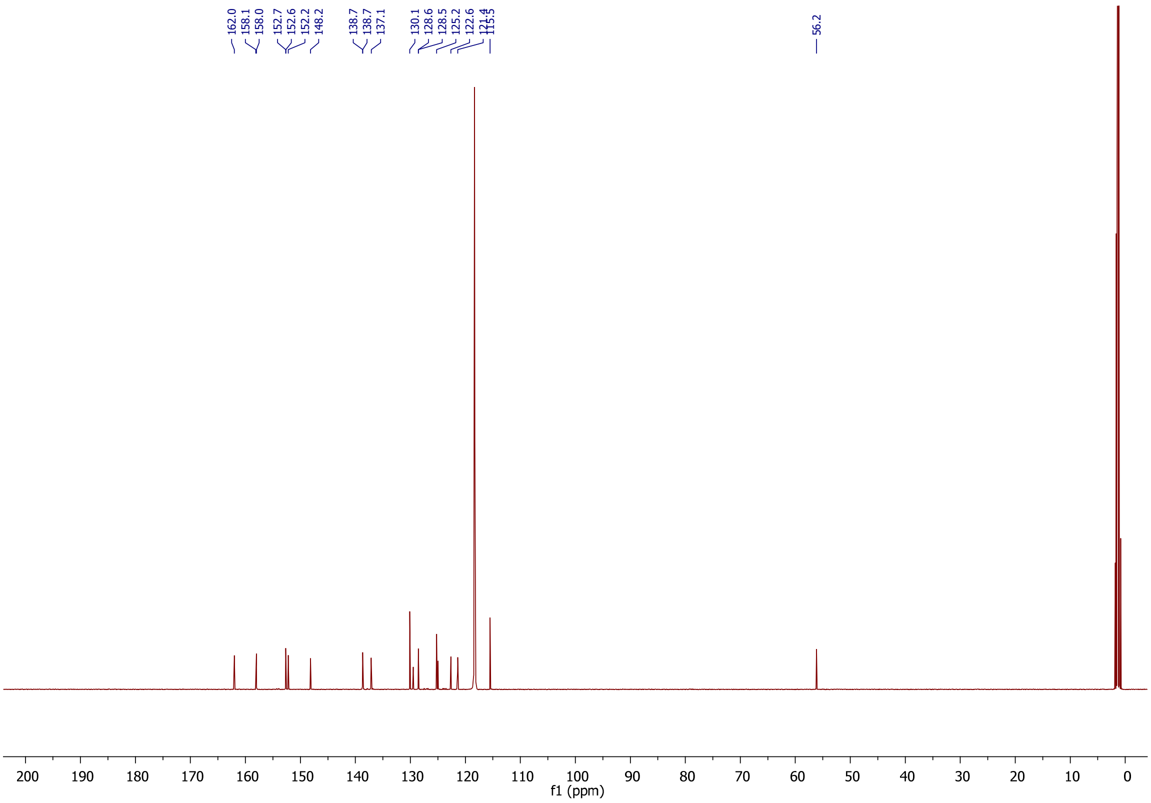

Figure 24. ^{13}C NMR spectrum of **7** in CD_3CN, 125 MHz.

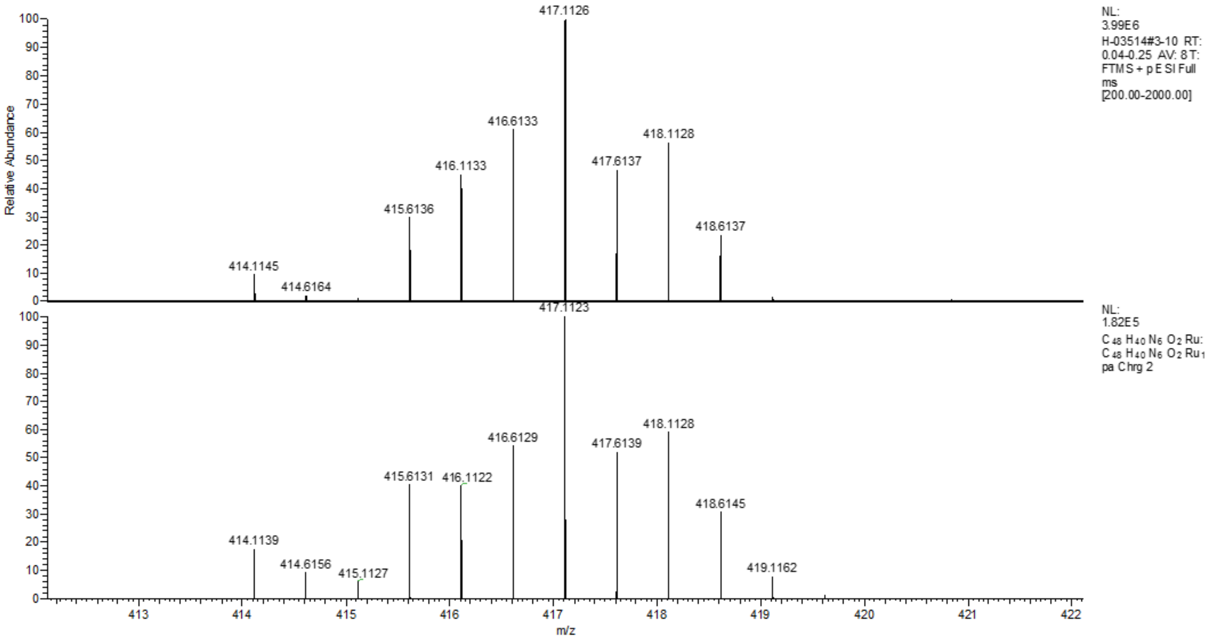

Figure 25. ESI-HRMS spectrum of **7** (positive detection mode).

Table 5. Crystal data and structure refinement parameters for L-H and L-NMe_2.

	**L-H**	**L-NMe_2**
CCDC number	1951466	1951468
Empirical formula	$C_{30}H_{28}N_2O_4$	$C_{30}H_{30}N_4$
Formula weight	480.54	446.58
Temperature/K	183(1)	183(1)
Crystal system	monoclinic	monoclinic
Space group	$P2_1/n$	C2/c
a/Å	8.55700(10)	17.3536(3)
b/Å	5.40730(10)	10.8262(2)
c/Å	26.8535(4)	25.5100(4)
α/°	90	90
β/°	96.8030(10)	92.7392(16)
γ/°	90	90
Volume/Å^3	1233.77(3)	4787.17(14)
Z	2	8
ρ_{calc}g/cm^3	1.294	1.239
μ/mm^{-1}	0.693	0.570
F(000)	508.0	1904.0
Crystal size/mm^3	0.15 × 0.14 × 0.05	0.25 × 0.08 × 0.03
Radiation	CuKα (λ = 1.54184)	CuKα (λ = 1.54184)
2Θ range for data collection/°	6.63 to 158.844	6.938 to 130.394
Index ranges	-10 ≤ h ≤ 10, -6 ≤ k ≤ 6, -34 ≤ l ≤ 33	-20 ≤ h ≤ 20, -12 ≤ k ≤ 12, -30 ≤ l ≤ 26
Reflections collected	11730	17913
Independent reflections	2660 [R_{int} = 0.0208, R_{sigma} = 0.0177]	4089 [R_{int} = 0.0402, R_{sigma} = 0.0288]
Data/restraints/parameters	2660/1/167	4089/0/311
Goodness-of-fit on F^2	1.047	1.070
Final R indexes [I>=2σ (I)]	R_1 = 0.0386, wR_2 = 0.1072	R_1 = 0.0837, wR_2 = 0.2575
Final R indexes [all data]	R_1 = 0.0421, wR_2 = 0.1102	R_1 = 0.0965, wR_2 = 0.2760
Largest diff. peak/hole / e Å^{-3}	0.18/-0.18	0.95/-0.18

Table 6. Crystal data and structure refinement parameters for L-OMe and **3**.

	**L-OMe**	**3**
CCDC number	1951469	1951467
Empirical formula	$C_{32}H_{32}N_2O_6$	$C_{173}H_{154}F_{24}N_{12}O_{13}P_4Ru_2$
Formula weight	540.59	3391.09
Temperature/K	183(1)	183(1)
Crystal system	monoclinic	monoclinic
Space group	$P2_1/c$	$P2_1/c$
a/Å	10.0268(2)	18.7906(2)
b/Å	40.2465(13)	22.1394(3)
c/Å	13.4342(4)	41.1669(5)
α/°	90	90
β/°	93.176(2)	91.7327(12)
γ/°	90	90
Volume/Å^3	5413.0(3)	17118.2(4)
Z	8	4
ρ_{calc}g/cm^3	1.327	1.316
μ/mm^{-1}	0.749	2.527
F(000)	2288.0	6976.0
Crystal size/mm^3	0.1 × 0.06 × 0.01	0.15 × 0.07 × 0.02
Radiation	CuKα (λ = 1.54184)	CuKα (λ = 1.54184)
2Θ range for data collection/°	6.946 to 136.478	6.172 to 136.496
Index ranges	$-12 \leq h \leq 11, -48 \leq k \leq 44, -15 \leq l \leq 13$	$-22 \leq h \leq 19, -25 \leq k \leq 23, -49 \leq l \leq 49$
Reflections collected	44916	148971
Independent reflections	9857 [R_{int} = 0.0587, R_{sigma} = 0.0423]	30784 [R_{int} = 0.0496, R_{sigma} = 0.0407]
Data/restraints/parameters	9857/0/733	30784/2365/1874
Goodness-of-fit on F^2	1.114	1.450
Final R indexes [I>=2σ (I)]	R_1 = 0.1003, wR_2 = 0.2765	R_1 = 0.1220, wR_2 = 0.3653
Final R indexes [all data]	R_1 = 0.1241, wR_2 = 0.2947	R_1 = 0.1597, wR_2 = 0.3936
Largest diff. peak/hole / e Å^{-3}	0.51/-0.37	1.73/-0.86

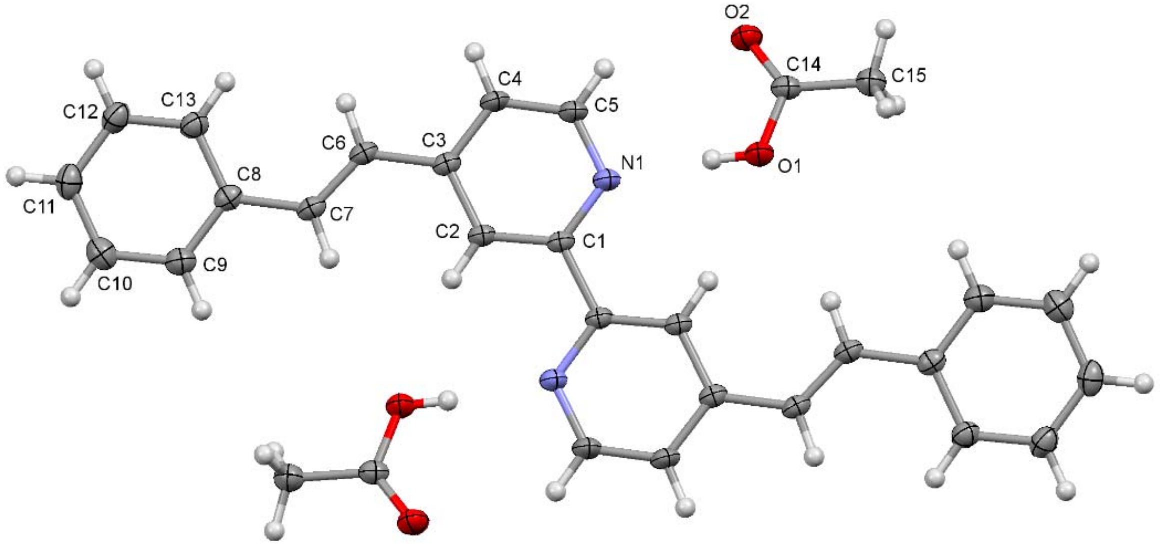

Figure 26. Molecular structure of L-H. The thermal ellipsoids are drawn at the 30 % probability level.

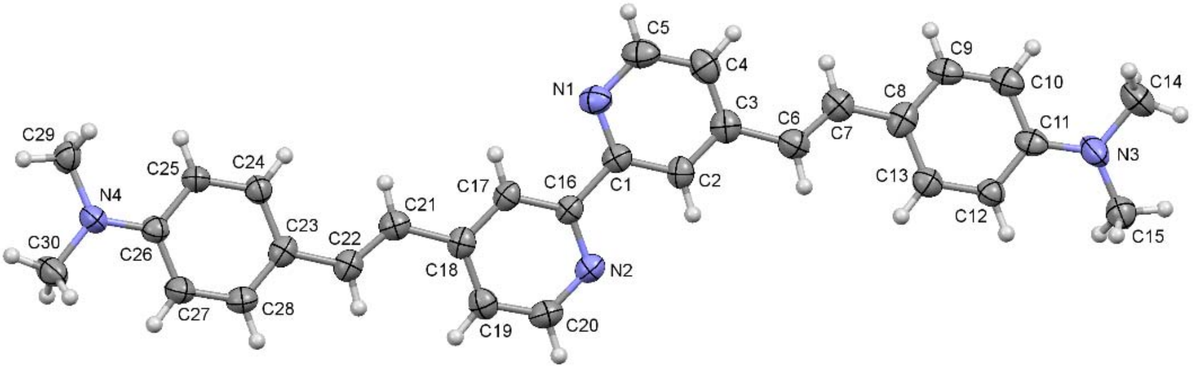

Figure 27. Molecular structure of L-NMe_2. The thermal ellipsoids are drawn at the 50 % probability level.

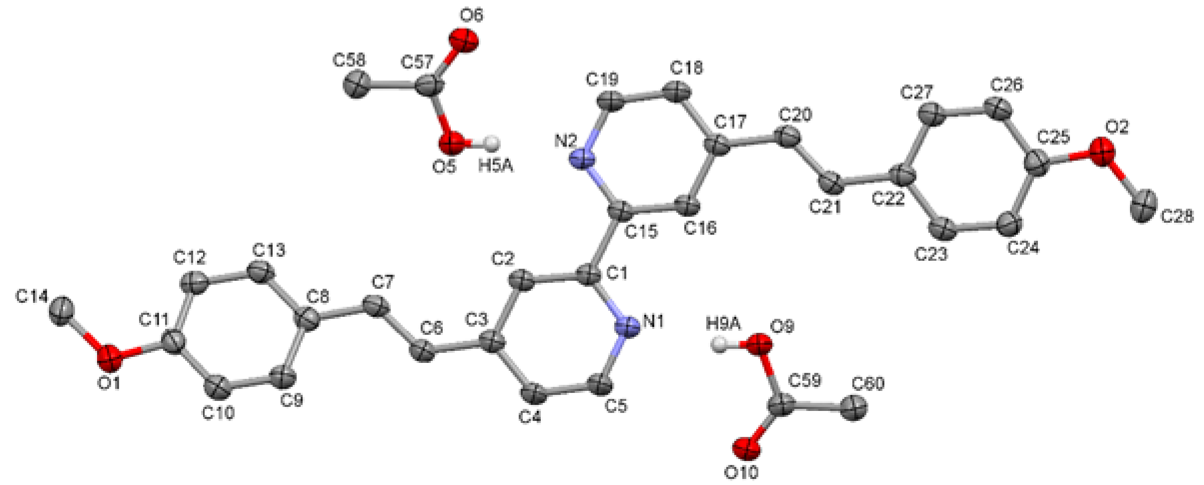

Figure 28. Molecular structure of L-OMe. The thermal ellipsoids are drawn at the 30 % probability level and selected H atoms are omitted for clarity.

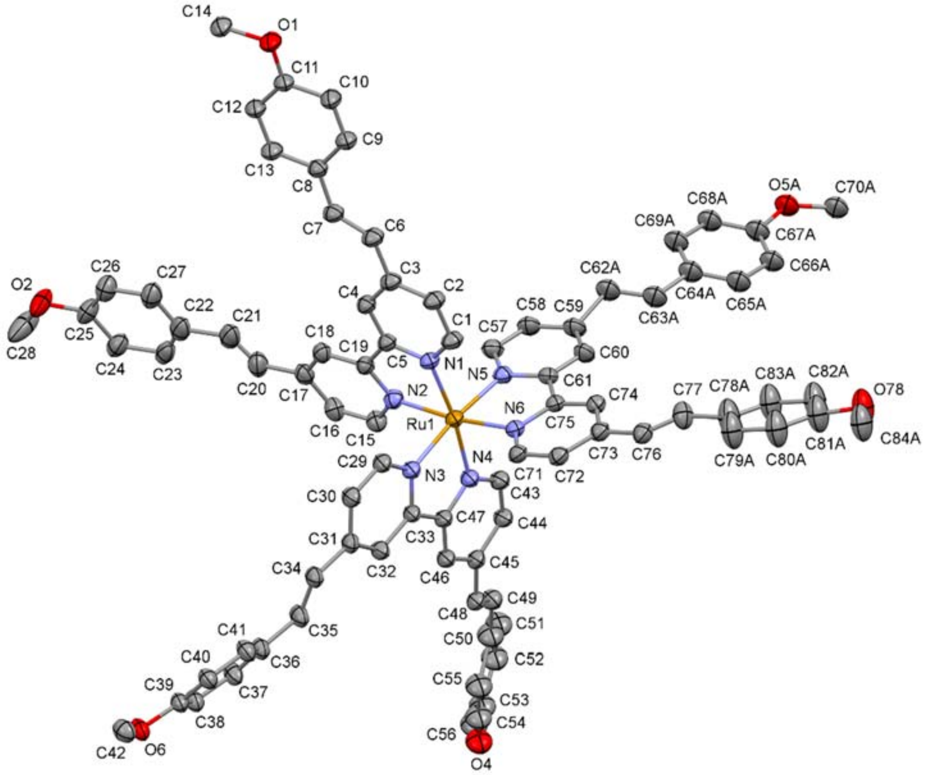

Figure 29. Molecular structure of **3**. One crystallographically independent molecule is presented in the Figure with thermal ellipsoids drawn at the 20 % probability level: counterions, disorders and all H atoms are omitted for clarity.

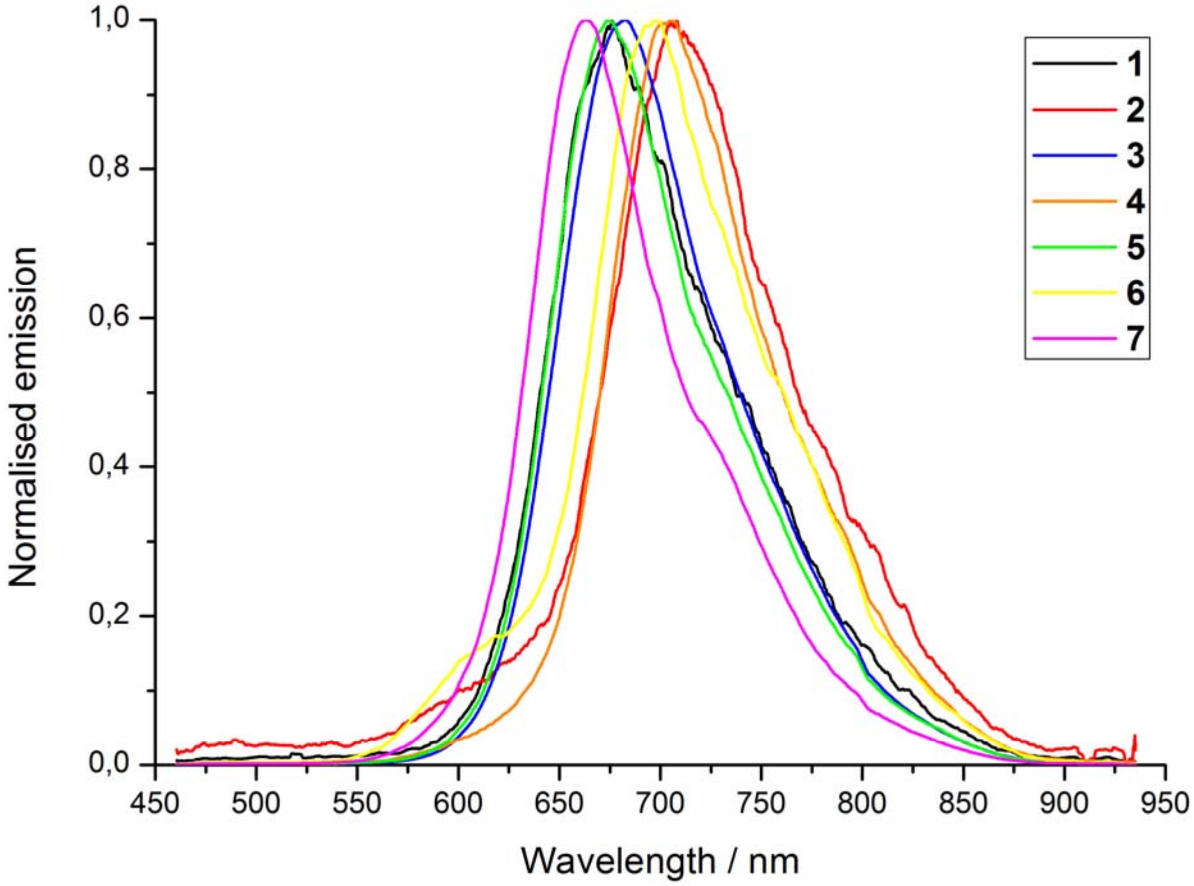

Figure 30. Normalised emission spectra of **1**-**7** in CH_3CN.

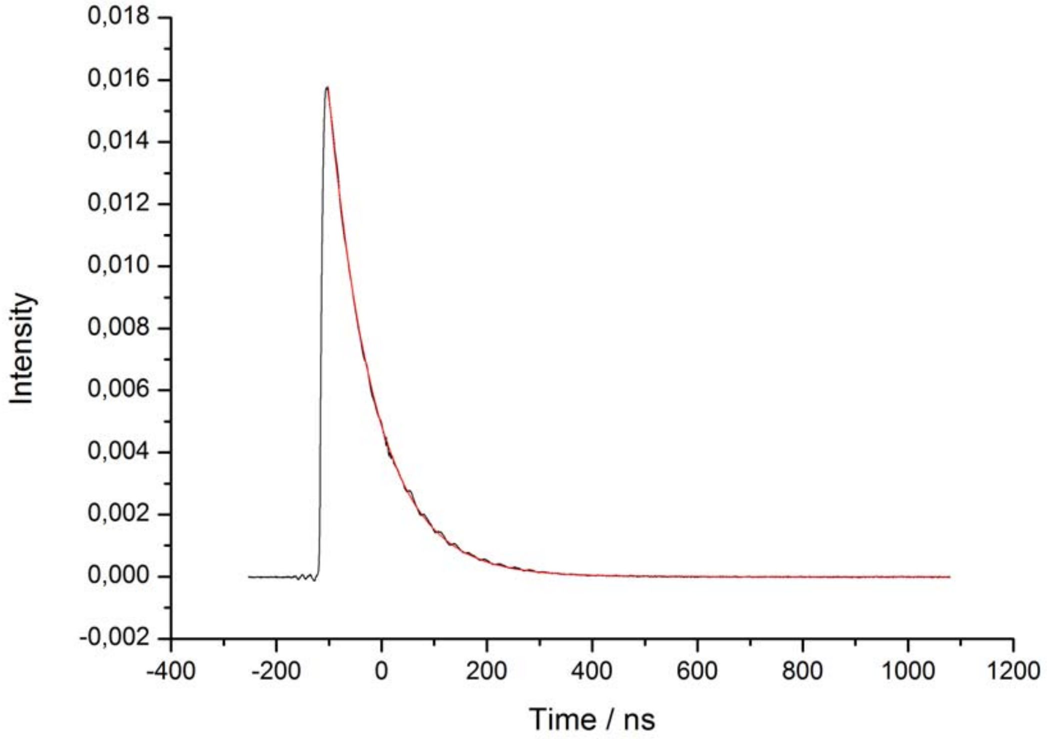

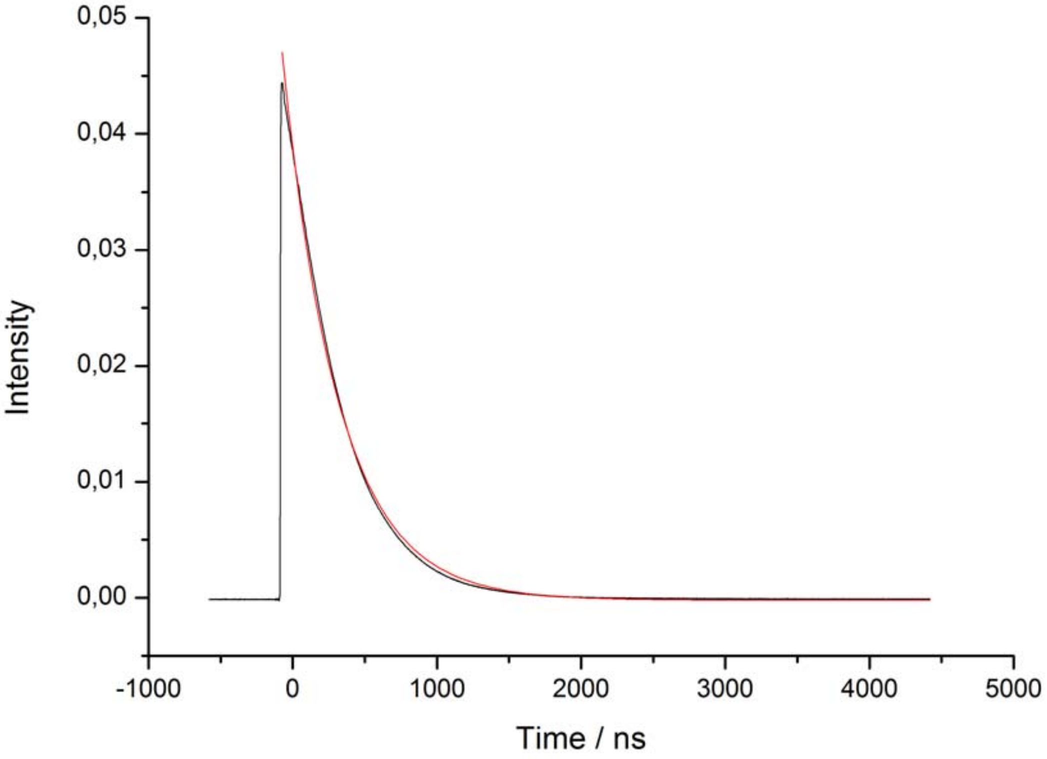

Figure 31. Lifetime spectra of the complex **1** in aerated (above) and degassed (below) CH_3CN.

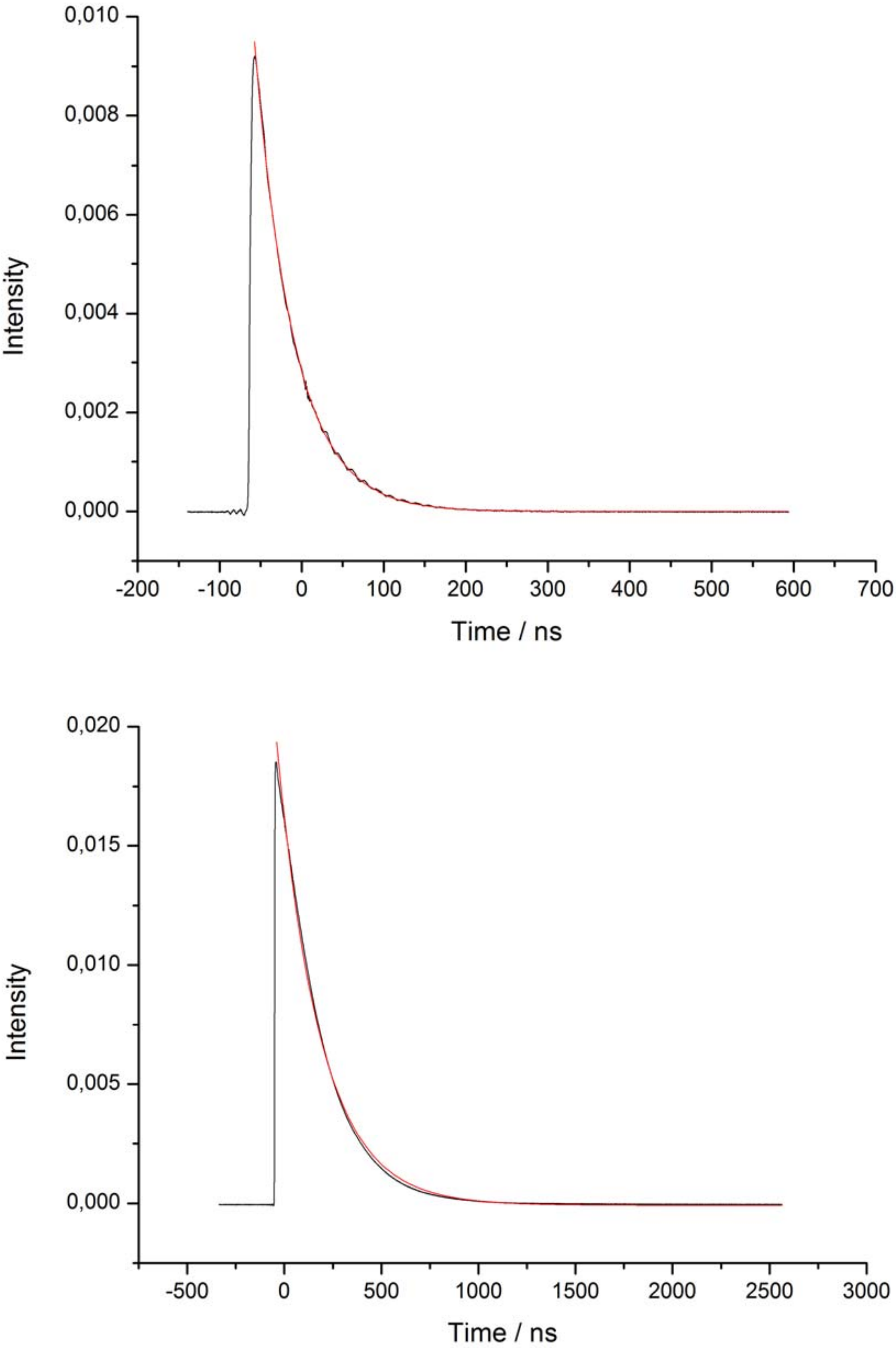

Figure 32. Lifetime spectra of the complex **2** in aerated (above) and degassed (below) CH_3CN.

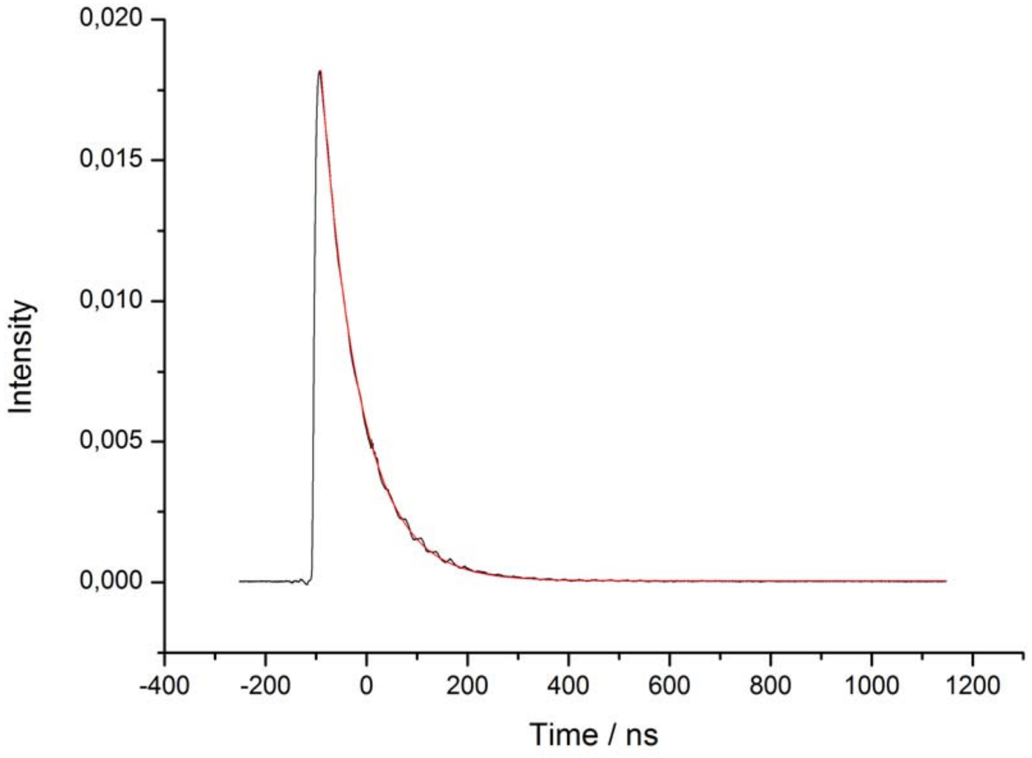

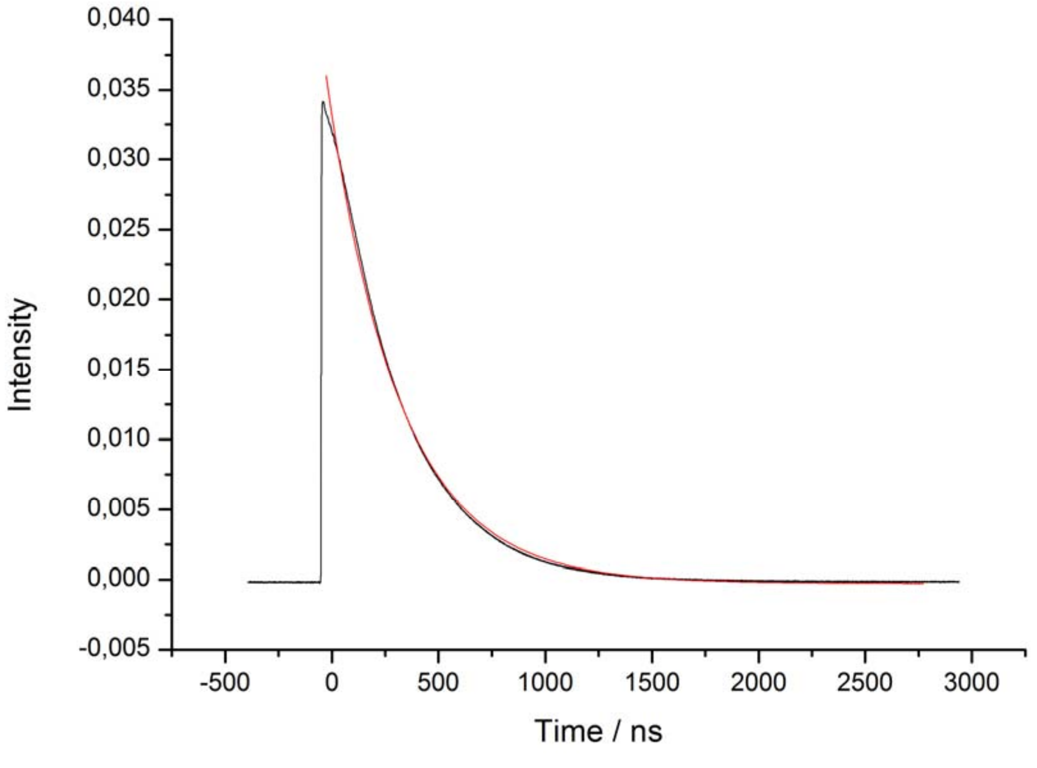

Figure 33. Lifetime spectra of the complex **3** in aerated (above) and degassed (below) CH_3CN.

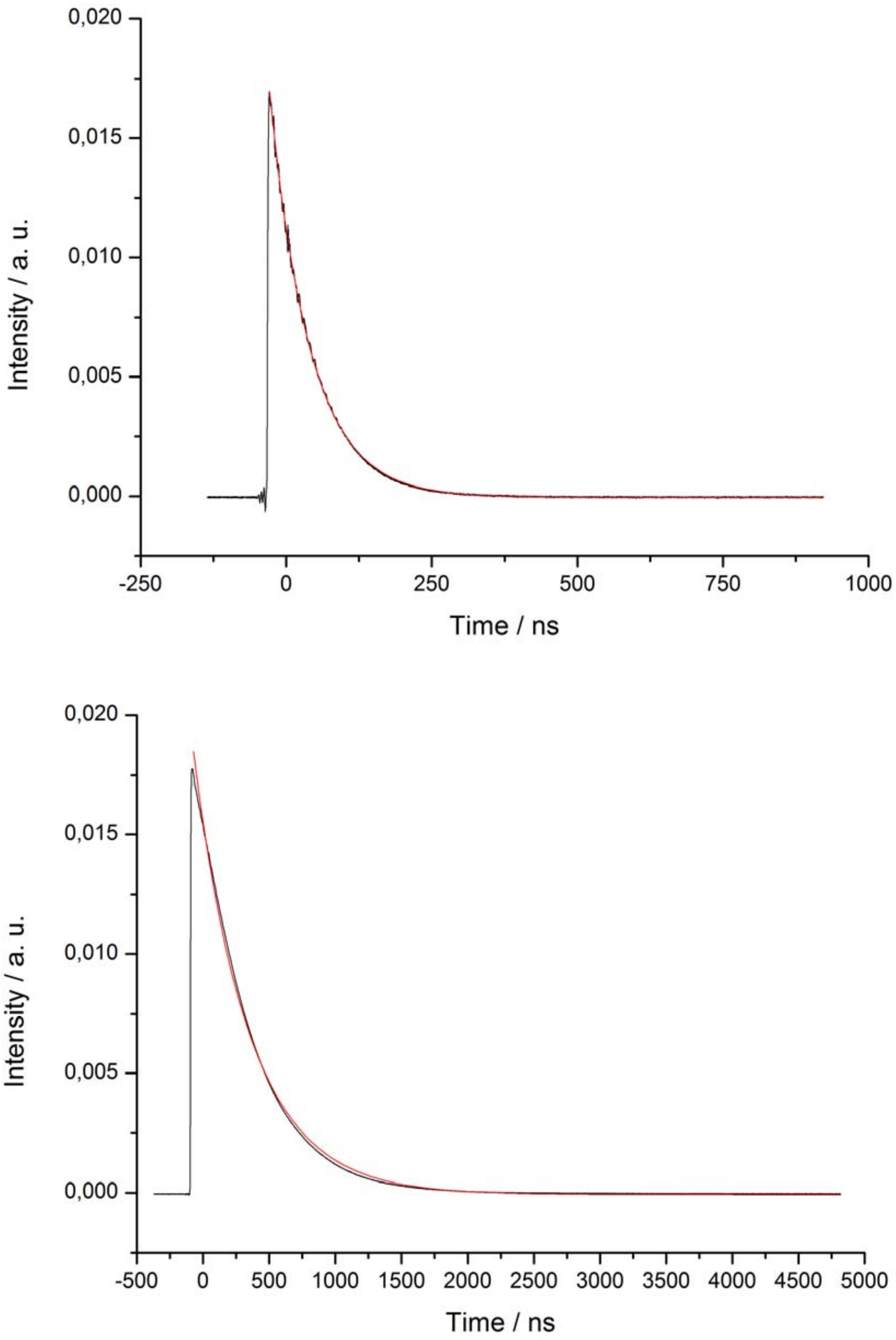

Figure 34. Lifetime spectra of the complex **4** in aerated (above) and degassed (below) CH_3CN.

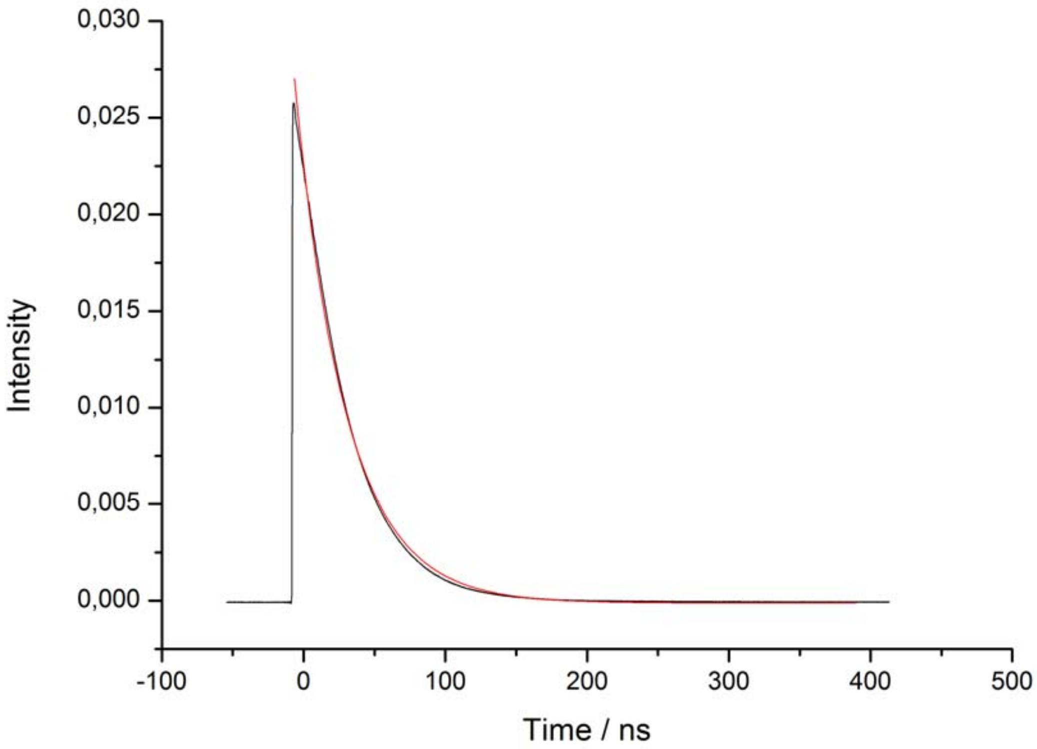

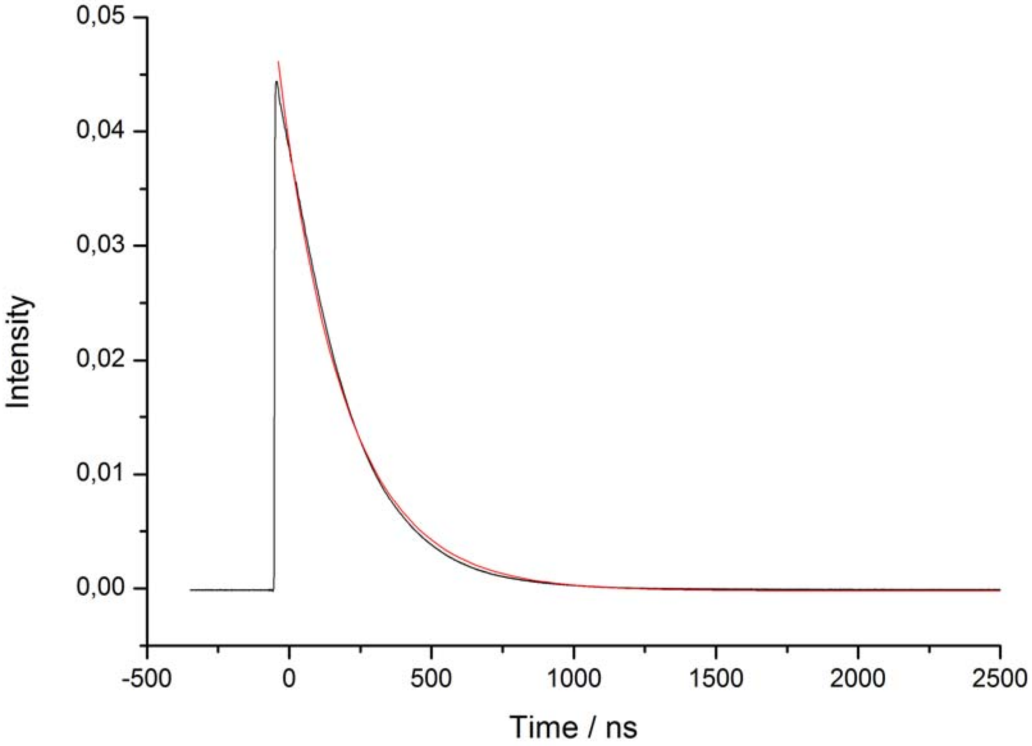

Figure 35. Lifetime spectra of the complex **5** in aerated (above) and degassed (below) CH_3CN.

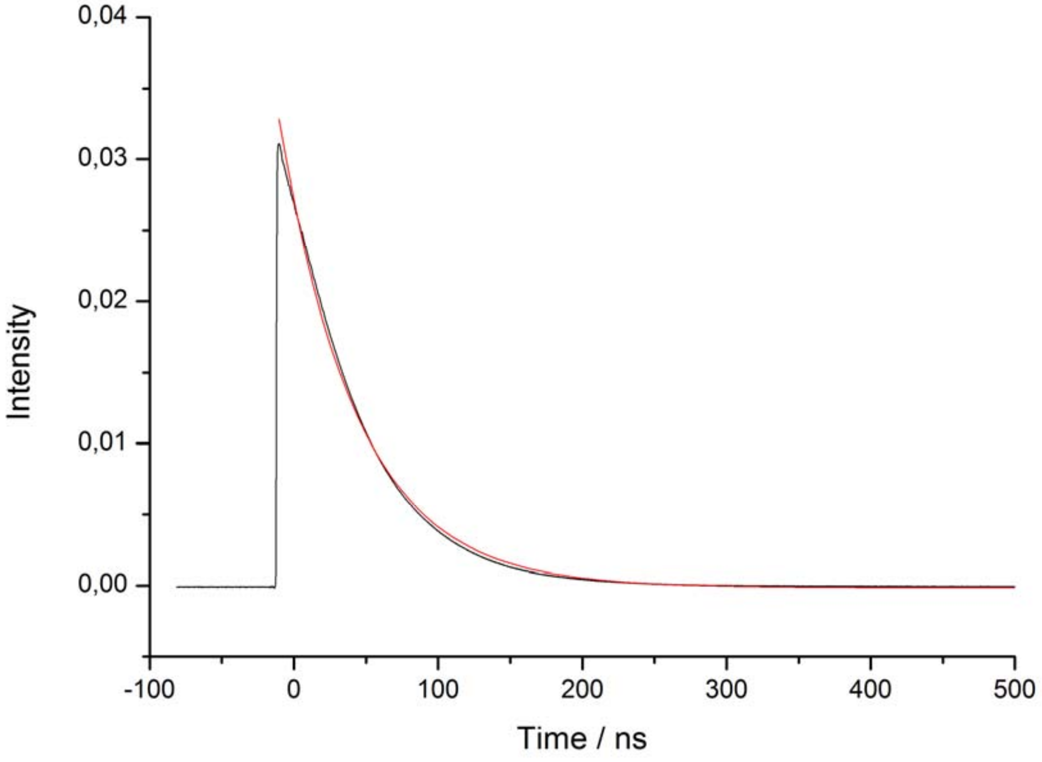

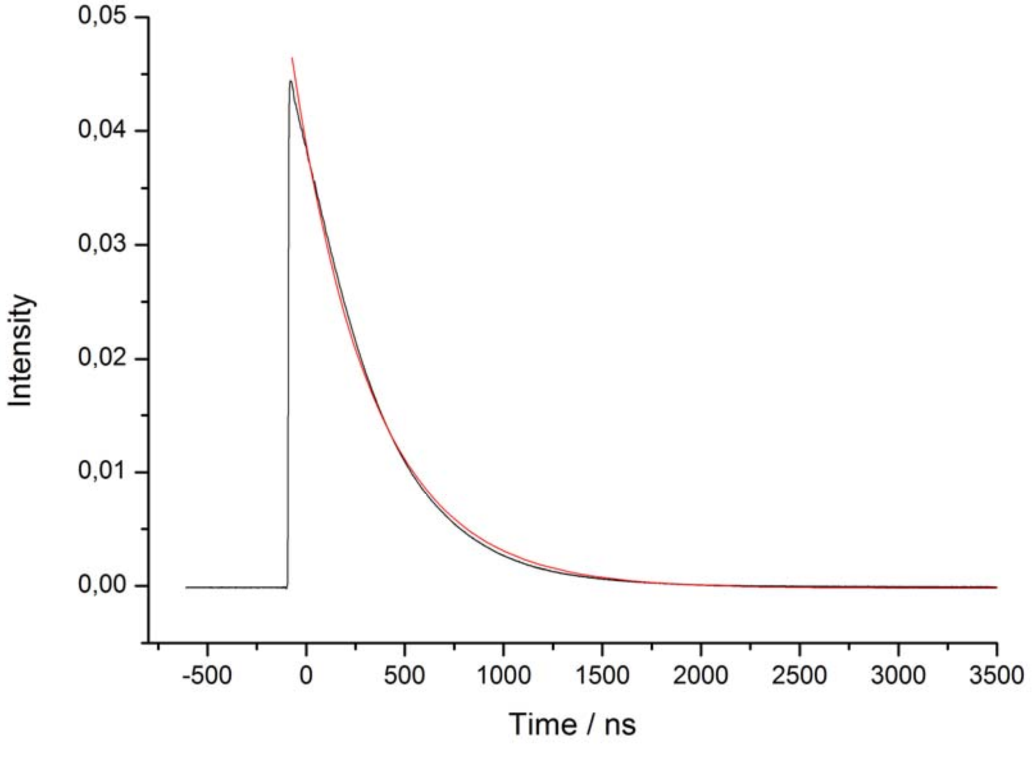

Figure 36. Lifetime spectra of the complex **6** in aerated (above) and degassed (below) CH_3CN.

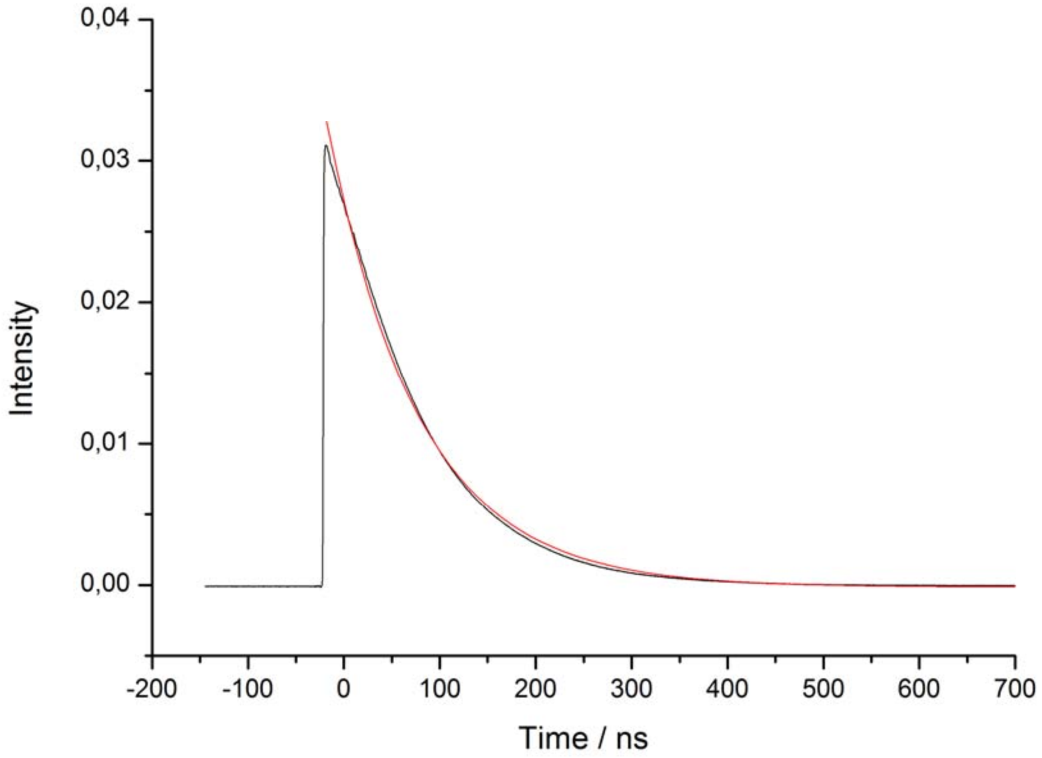

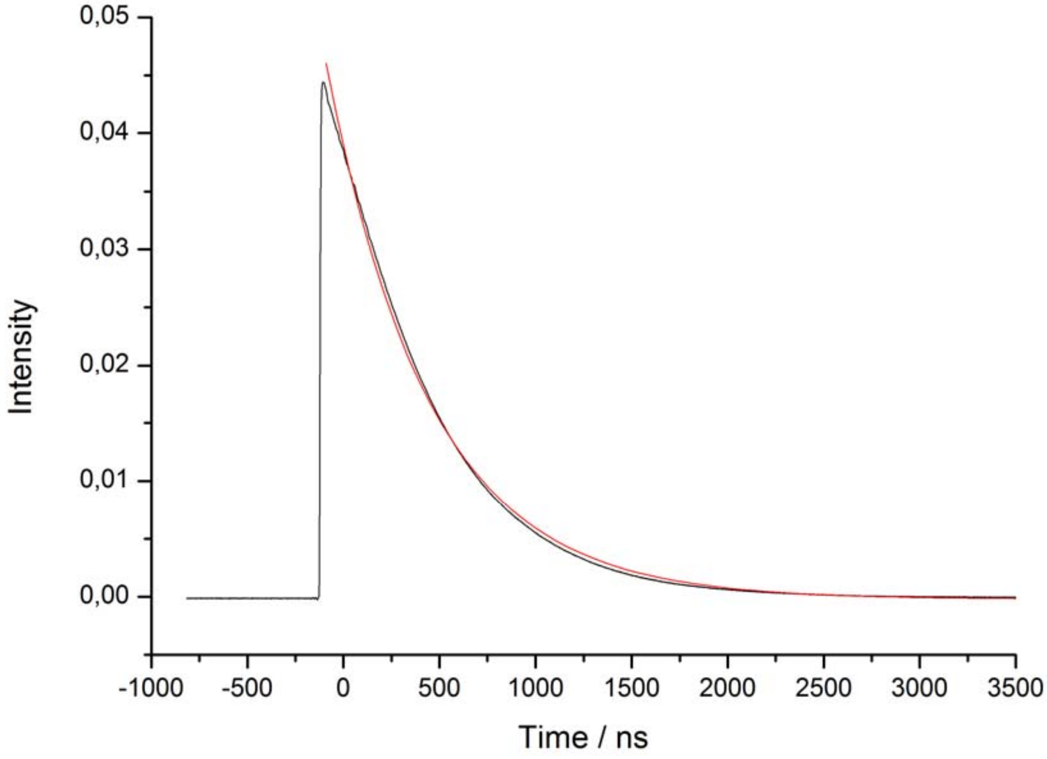

Figure 37. Lifetime spectra of the complex **7** in aerated (above) and degassed (below) CH_3CN.

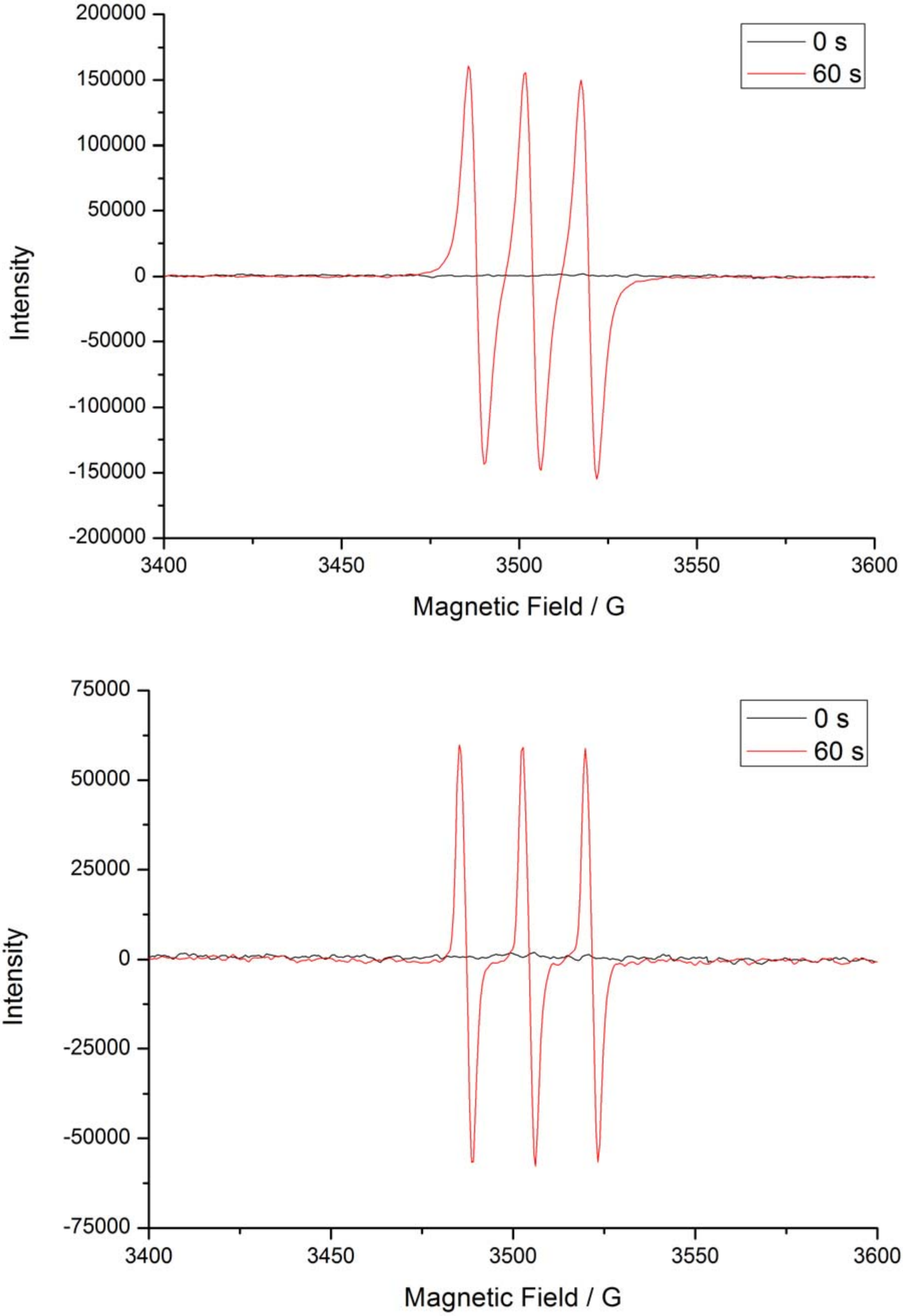

Figure 38. ESR spectra of the complex **1** trapped by TEMP in CH_3CN (above) or PBS (below).

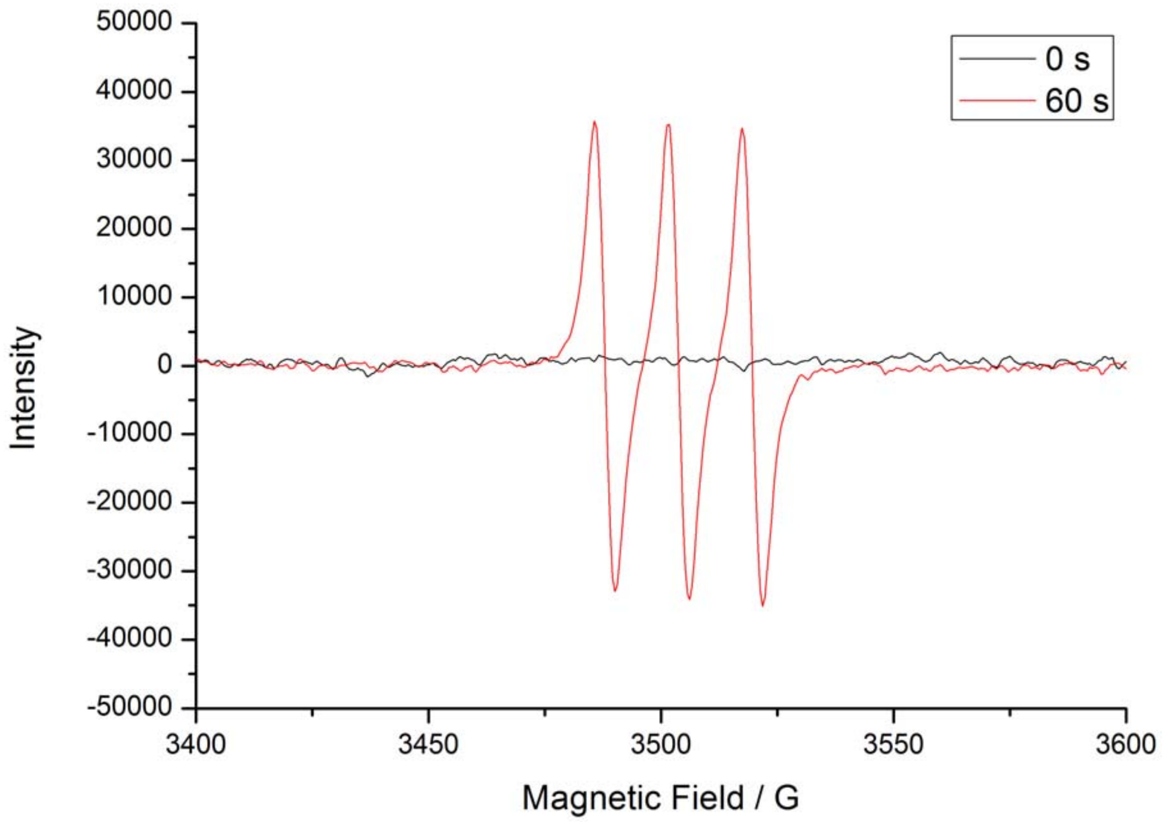

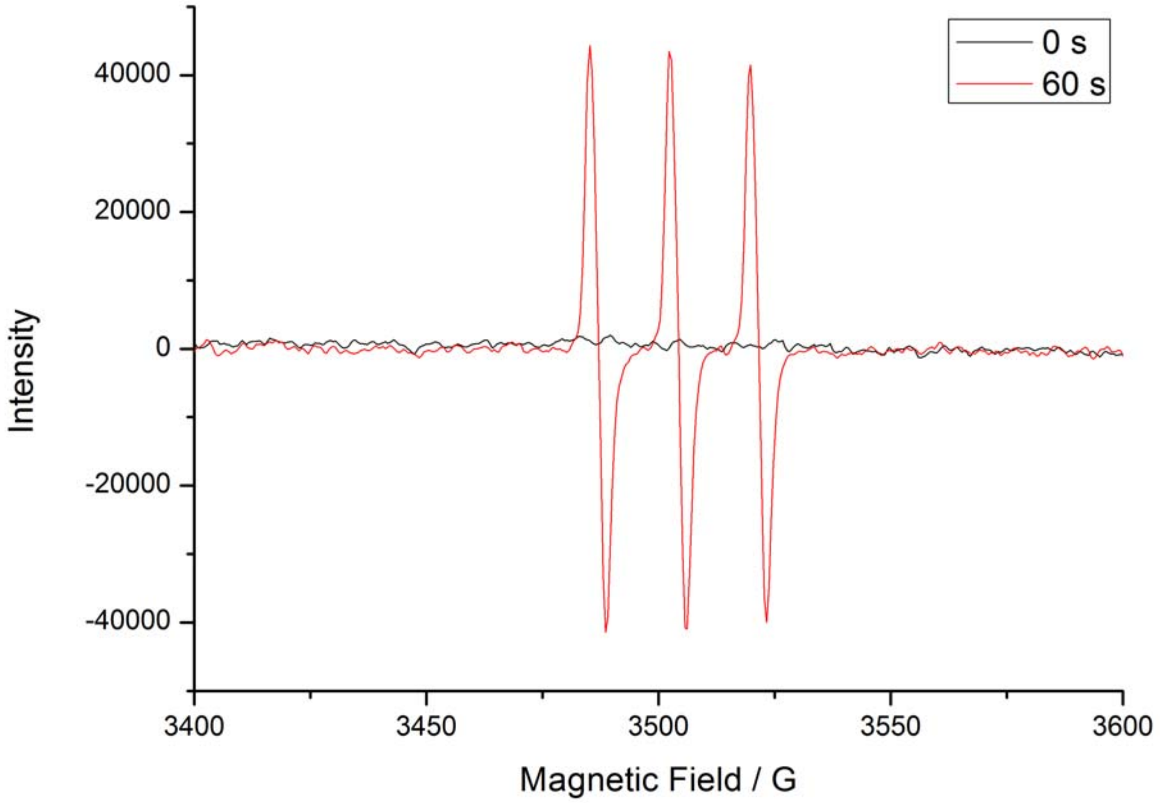

Figure 39. ESR spectra of the complex **2** trapped by TEMP in CH_3CN (above) or PBS (below).

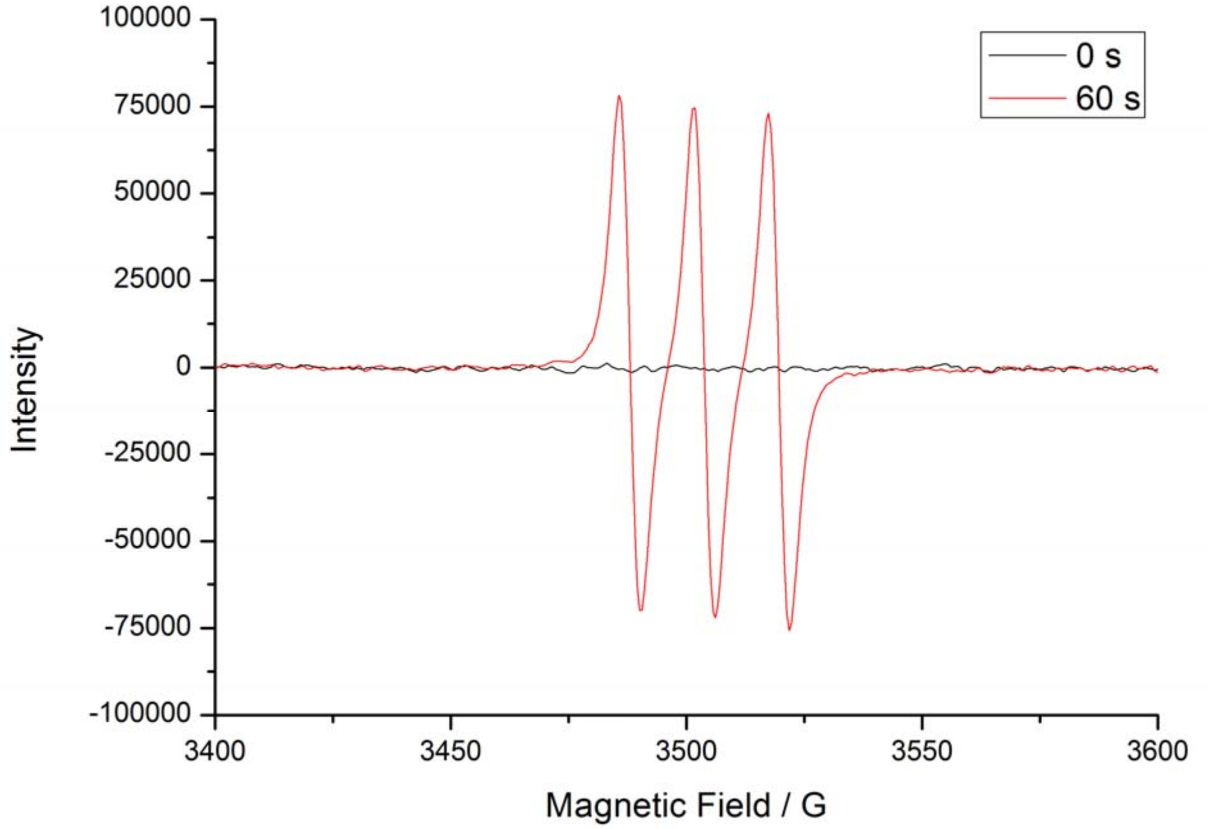

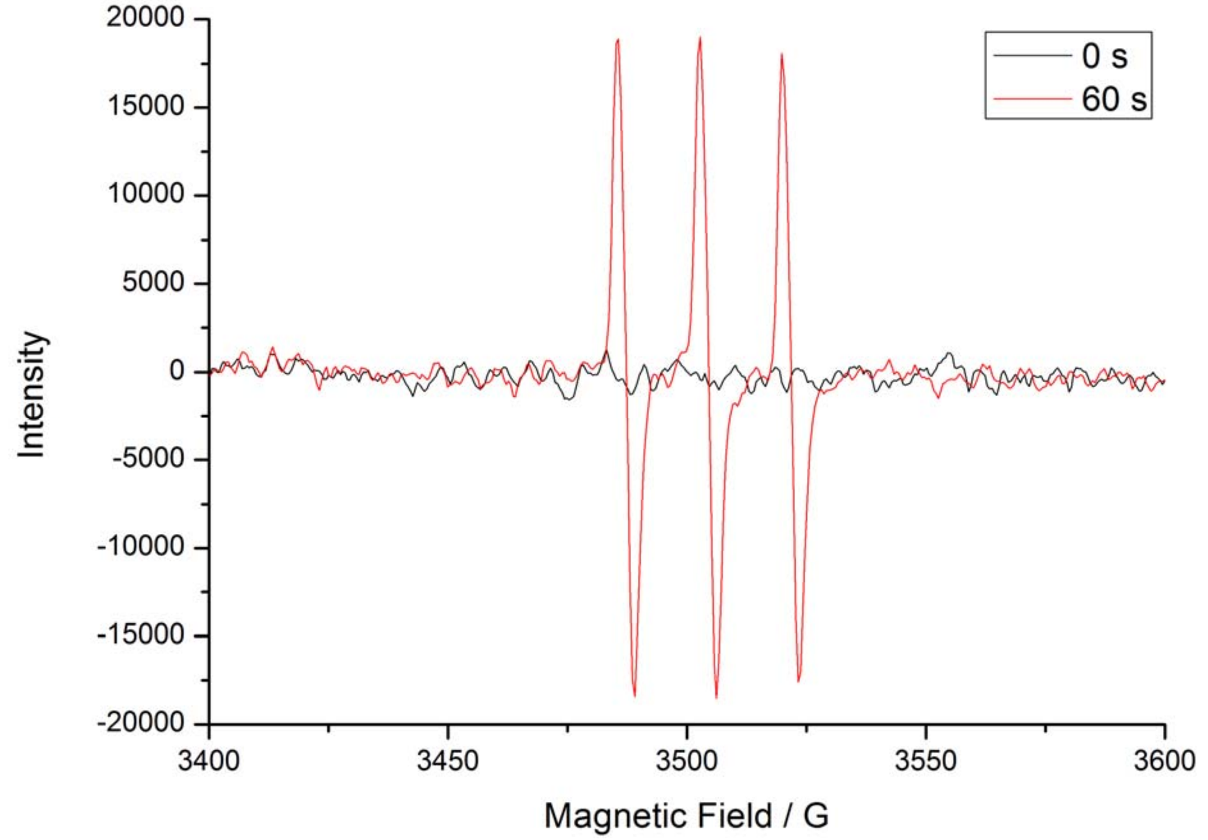

Figure 40. ESR spectra of the complex **3** trapped by TEMP in CH_3CN (above) or PBS (below).

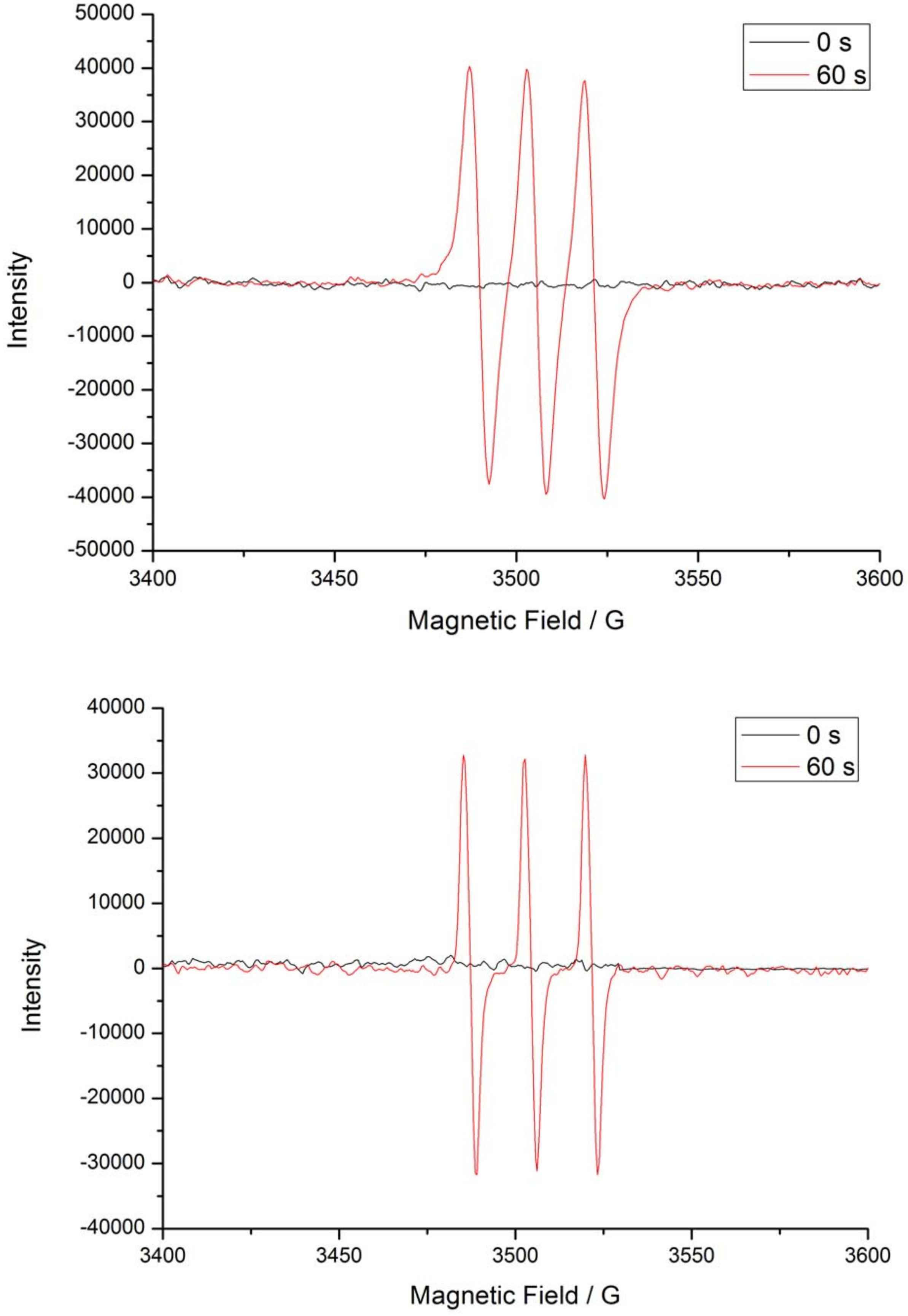

Figure 41. ESR spectra of the complex **4** trapped by TEMP in CH_3CN (above) or PBS (below).

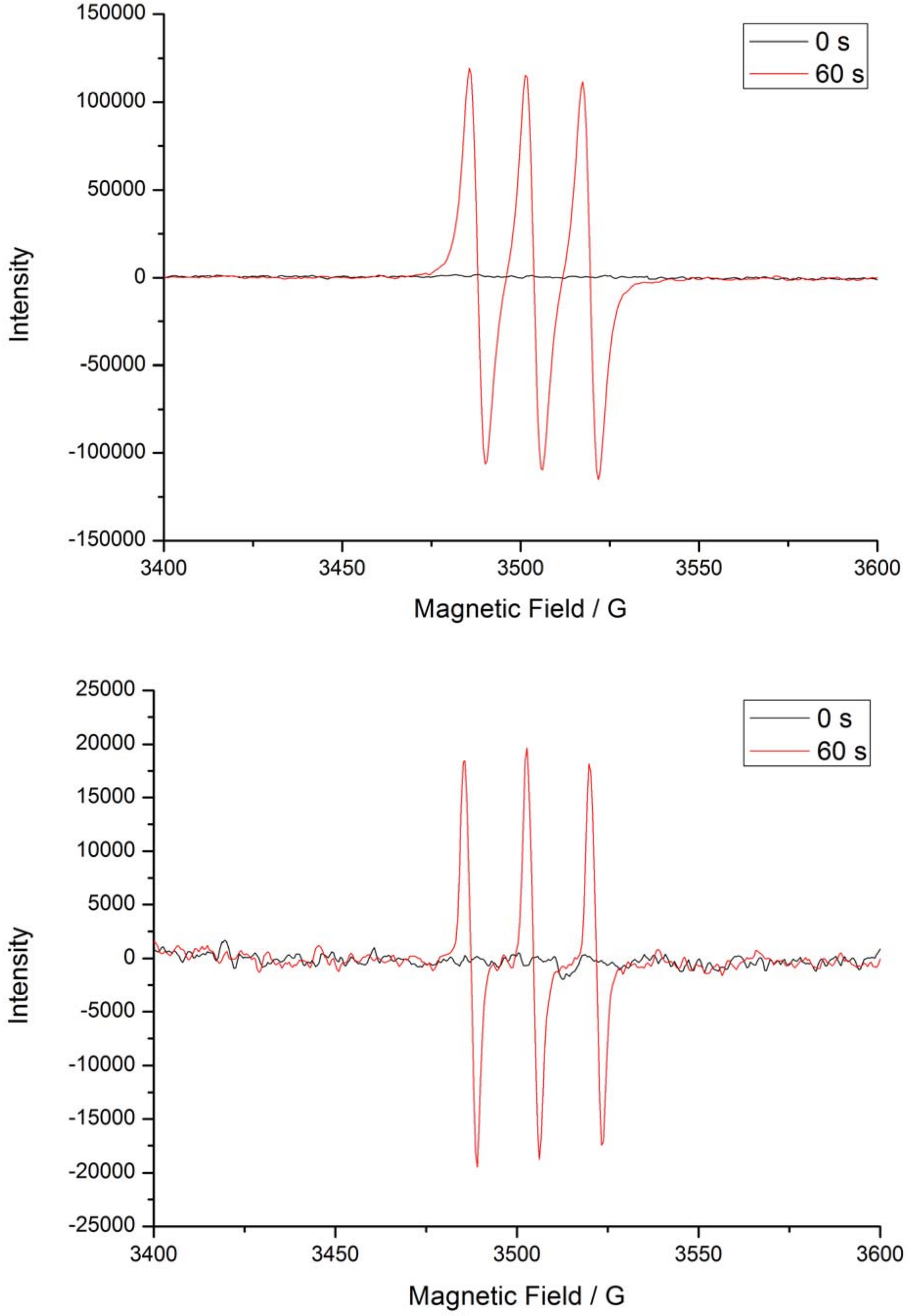

Figure 42. ESR spectra of the complex **5** trapped by TEMP in CH_3CN (above) or PBS (below).

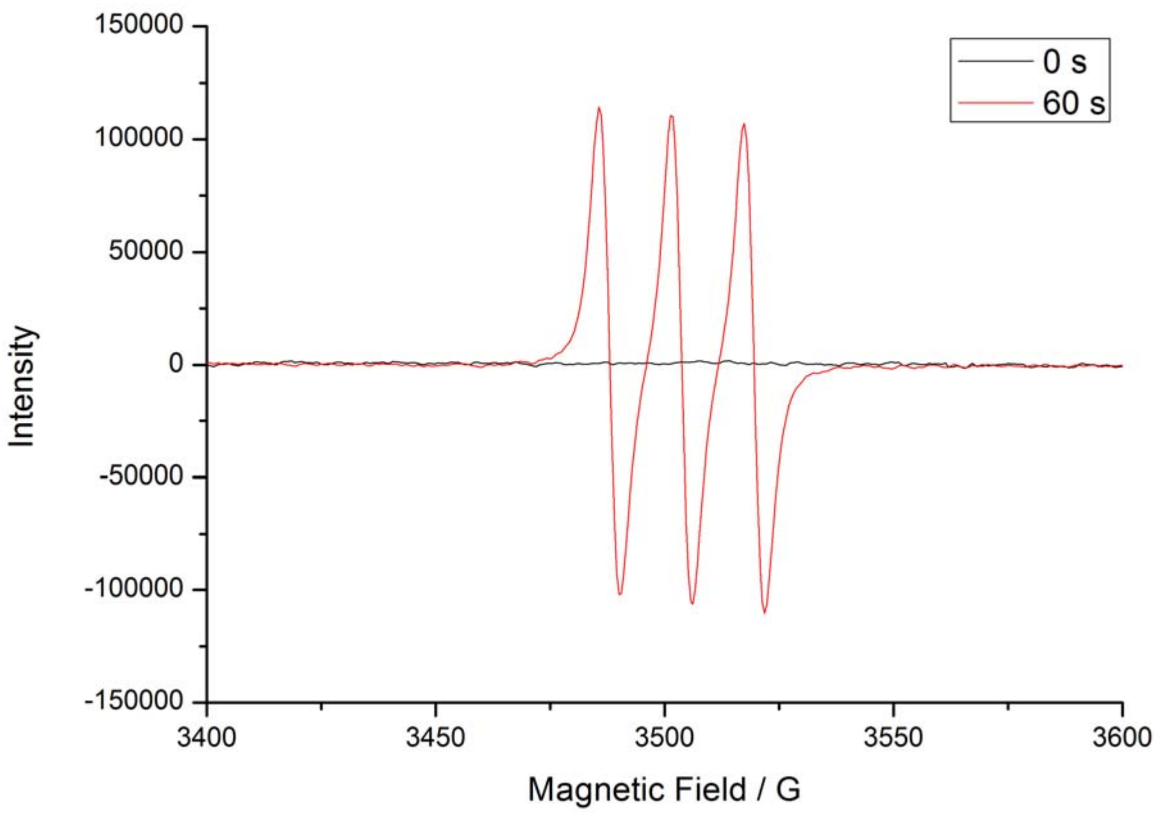

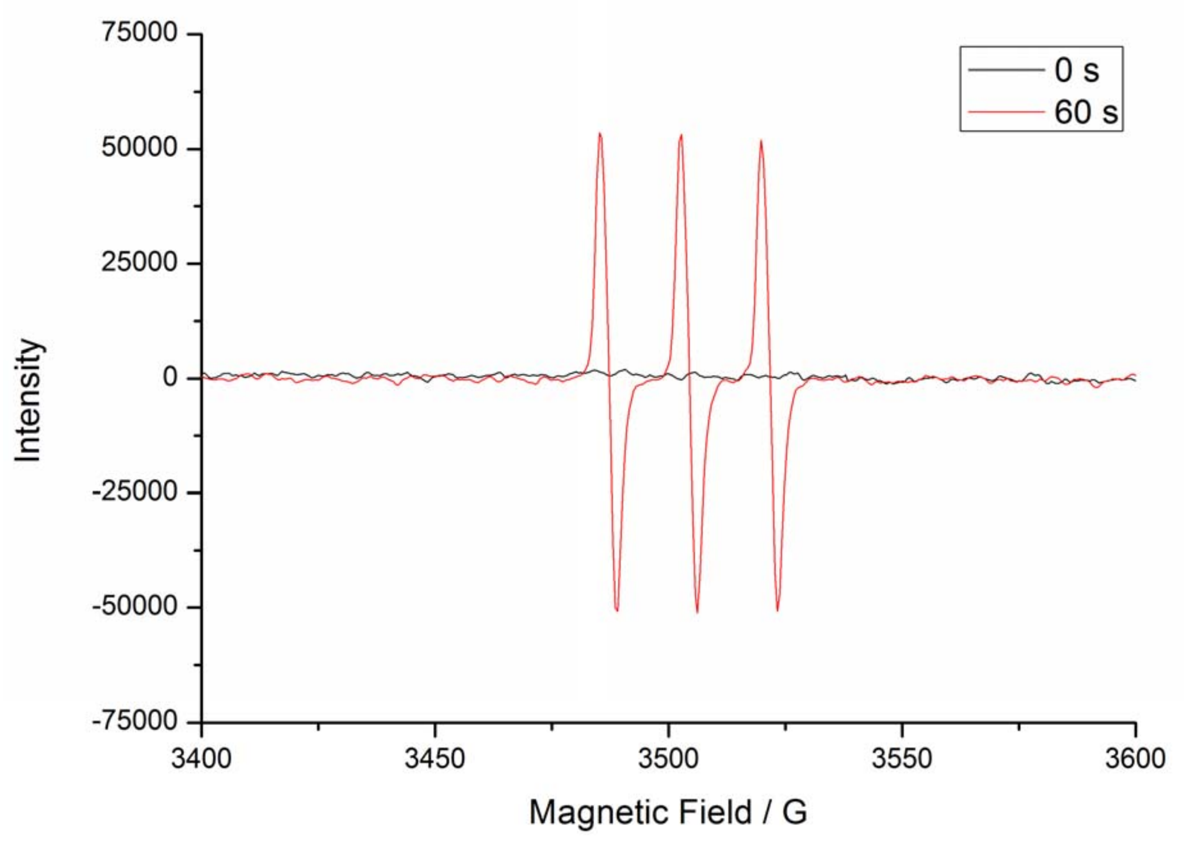

Figure 43. ESR spectra of the complex **6** trapped by TEMP in CH_3CN (above) or PBS (below).

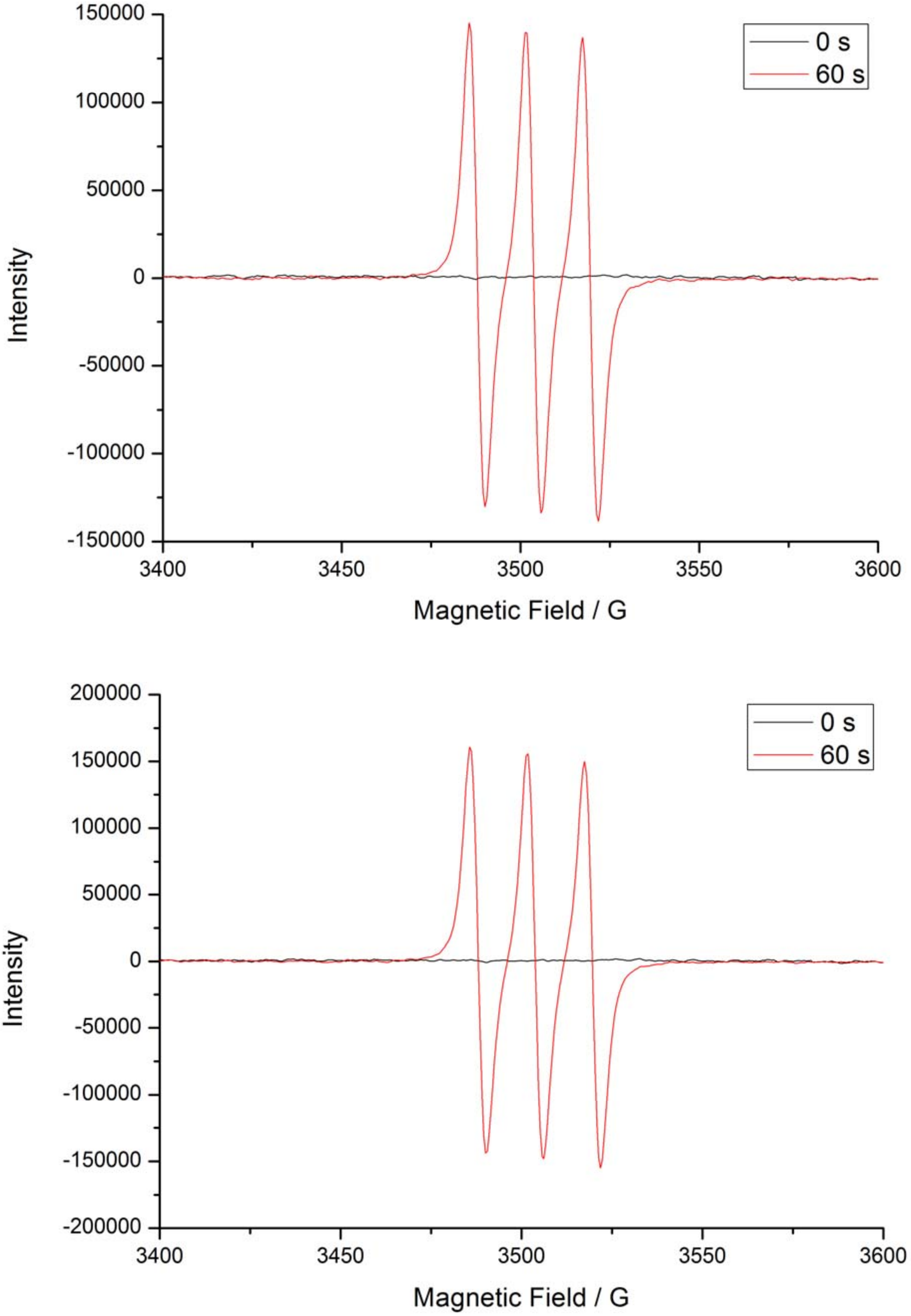

Figure 44. ESR spectra of the complex **7** trapped by TEMP in CH_3CN (above) or PBS (below).

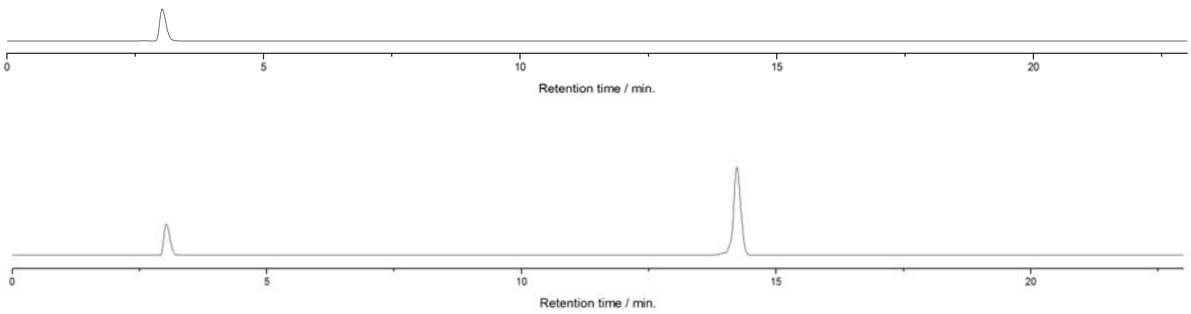

Figure 45. HPLC chromatogram (Method: M1) of caffeine (internal standard, above) and **1** after 48 h (below) incubation in human plasma.

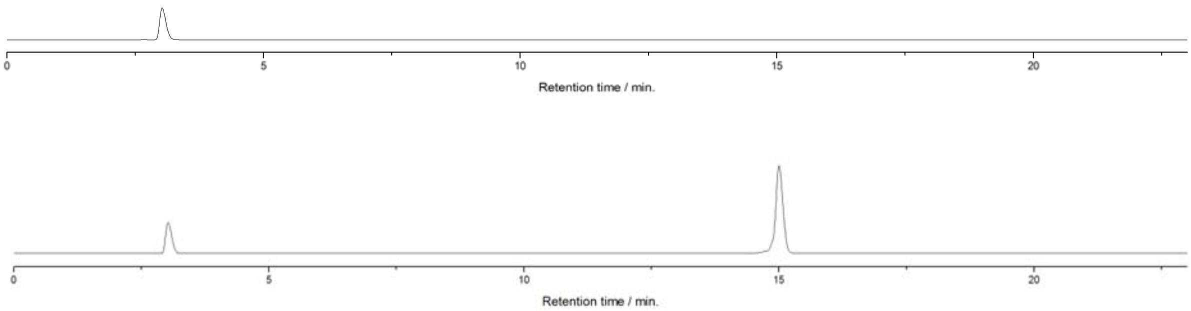

Figure 46. HPLC chromatogram (Method: M1) of caffeine (internal standard, above) and **2** after 48 h (below) incubation in human plasma.

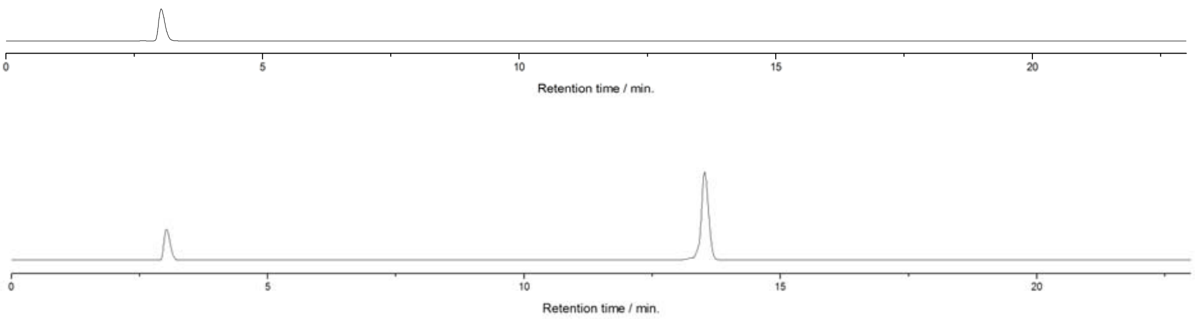

Figure 47. HPLC chromatogram of (Method: M1) caffeine (internal standard, above) and **3** after 48 h (below) incubation in human plasma.

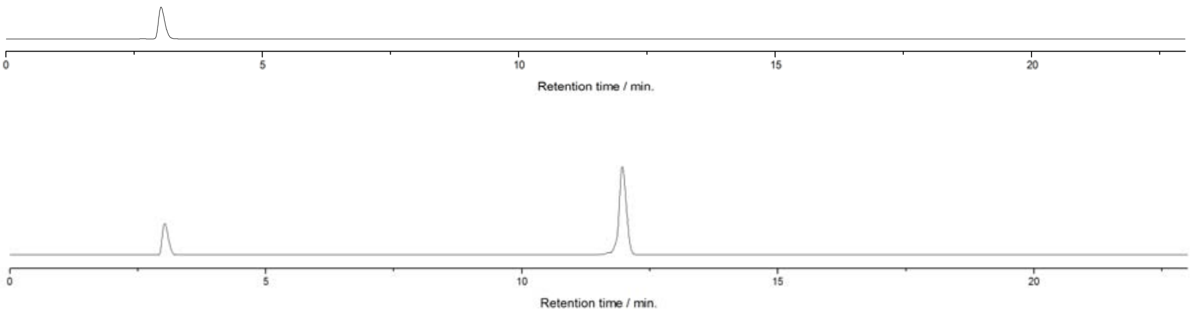

Figure 48. HPLC chromatogram (Method: M1) of caffeine (internal standard, above) and **4** after 48 h (below) incubation in human plasma.

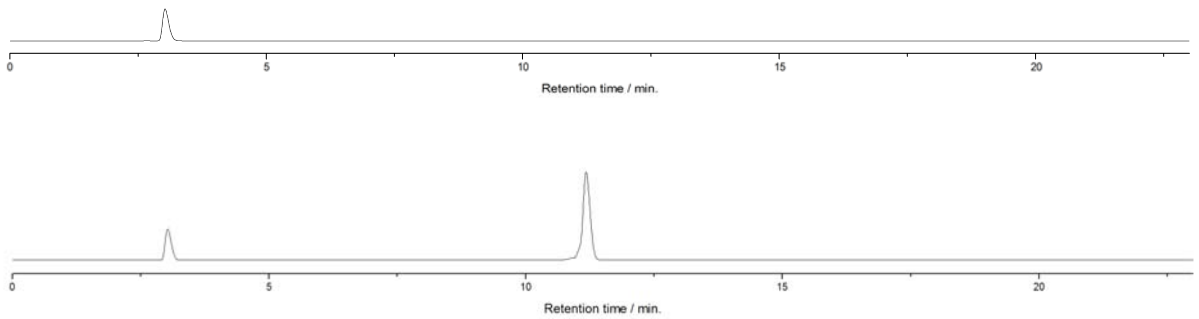

Figure 49. HPLC chromatogram (Method: M1) of caffeine (internal standard, above) and **5** after 48 h (below) incubation in human plasma.

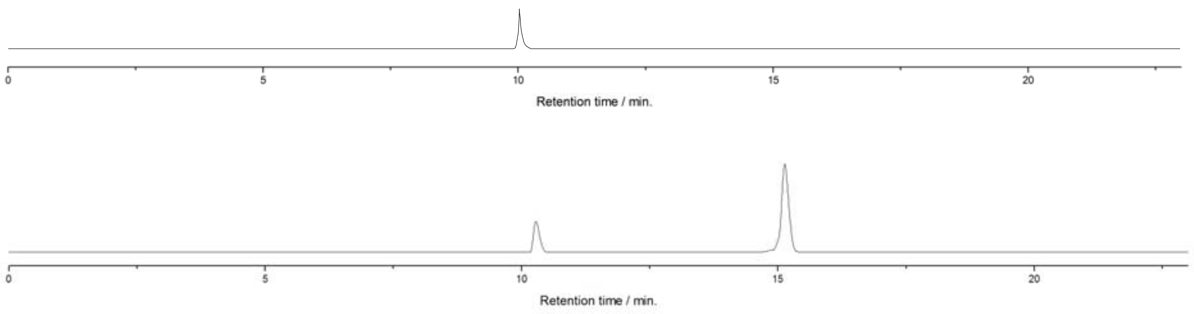

Figure 50. HPLC chromatogram (Method: M2) of caffeine (internal standard, above) and **6** after 48 h (below) incubation in human plasma.

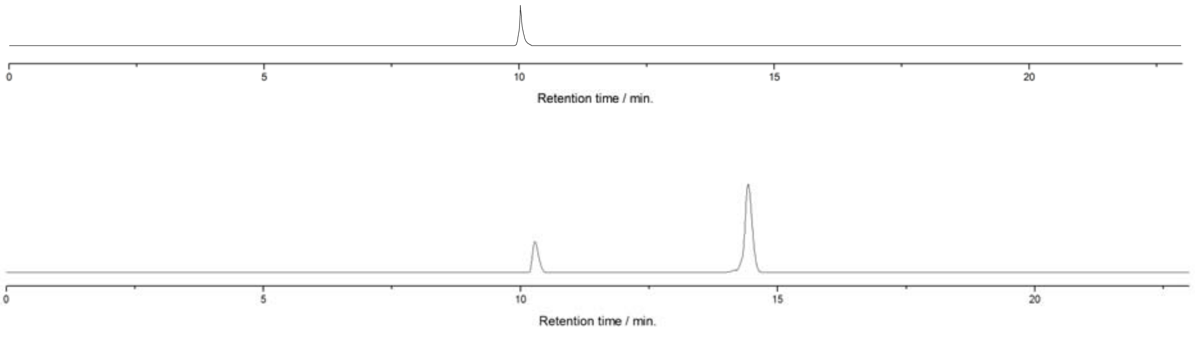

Figure 51. HPLC chromatogram (Method: M2) of Caffeine (internal standard, above) and **7** after 48 h (below) incubation in human plasma.

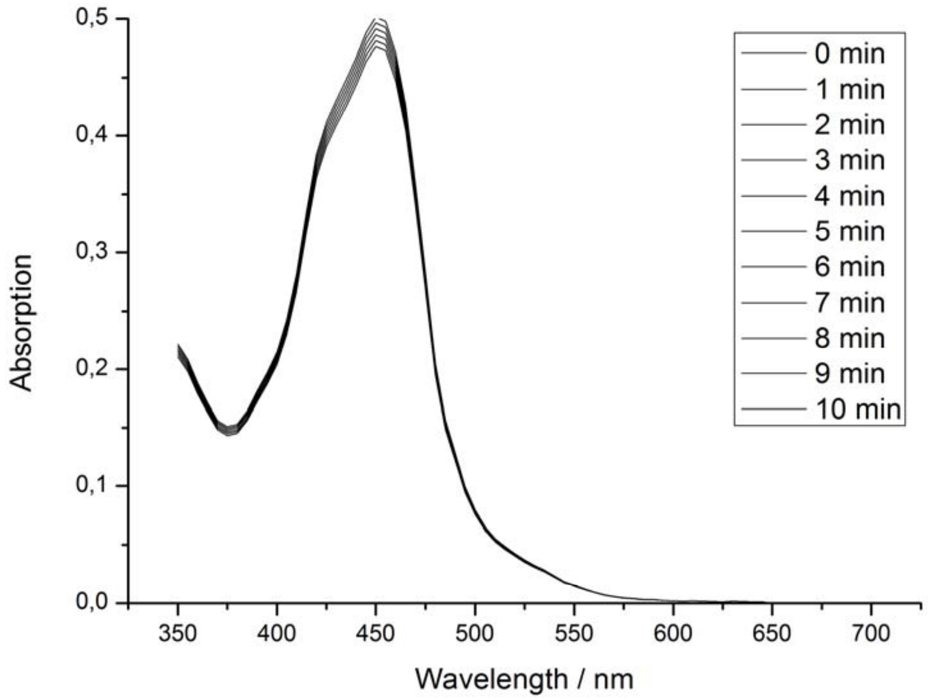

Figure 52. Temporal change of the UV/Vis spectra of $[Ru(bipy)_3]Cl_2$ by irradiation at 450 nm in CH_3CN.

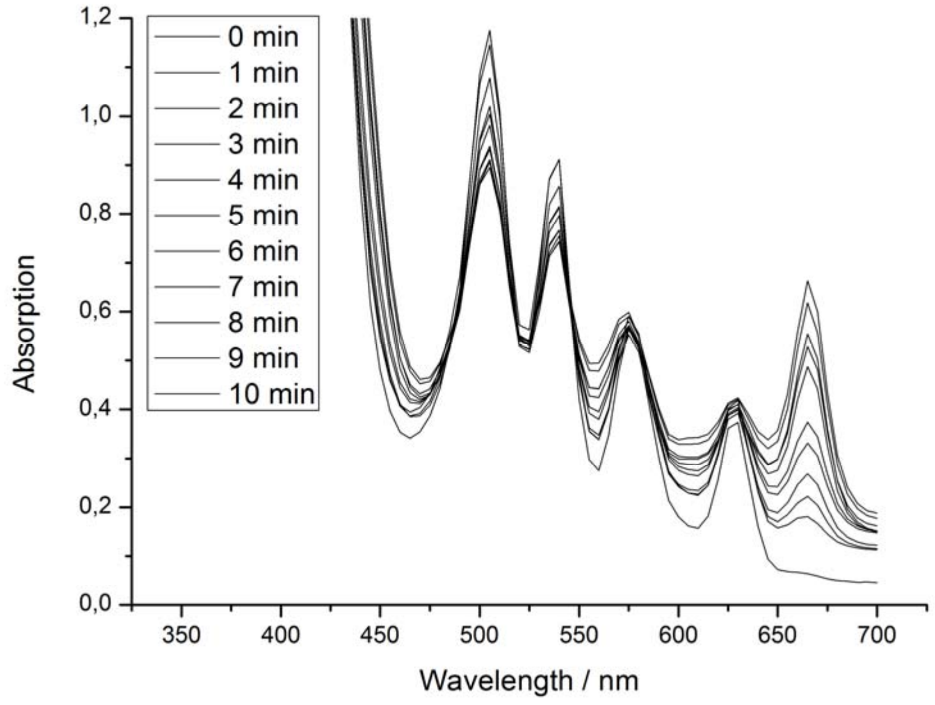

Figure 53. Temporal change of the UV/Vis spectra of Protoporphyrin IX by irradiation at 450 nm in CH_3CN.

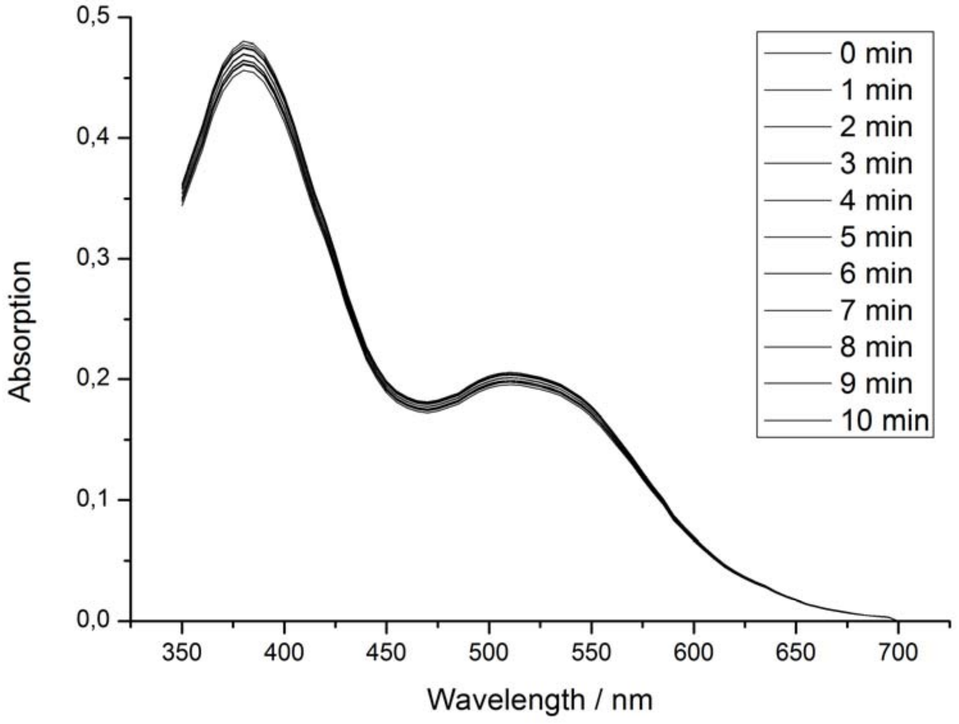

Figure 54. Temporal change of the UV/Vis spectra of **1** by irradiation at 450 nm in CH_3CN.

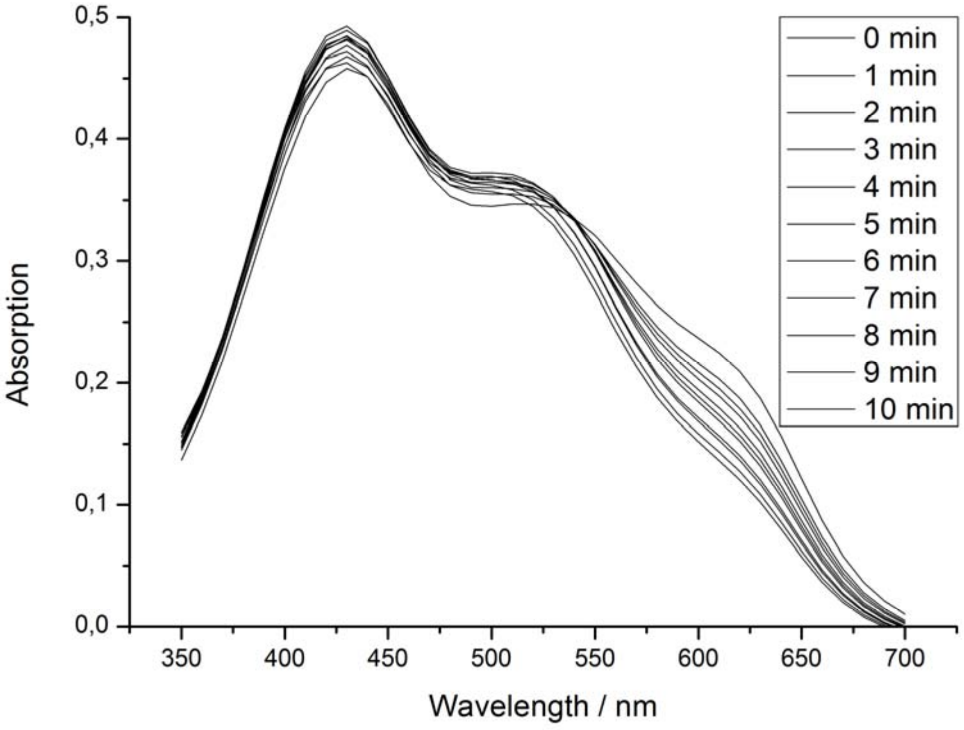

Figure 55. Temporal change of the UV/Vis spectra of **2** by irradiation at 450 nm in CH_3CN.

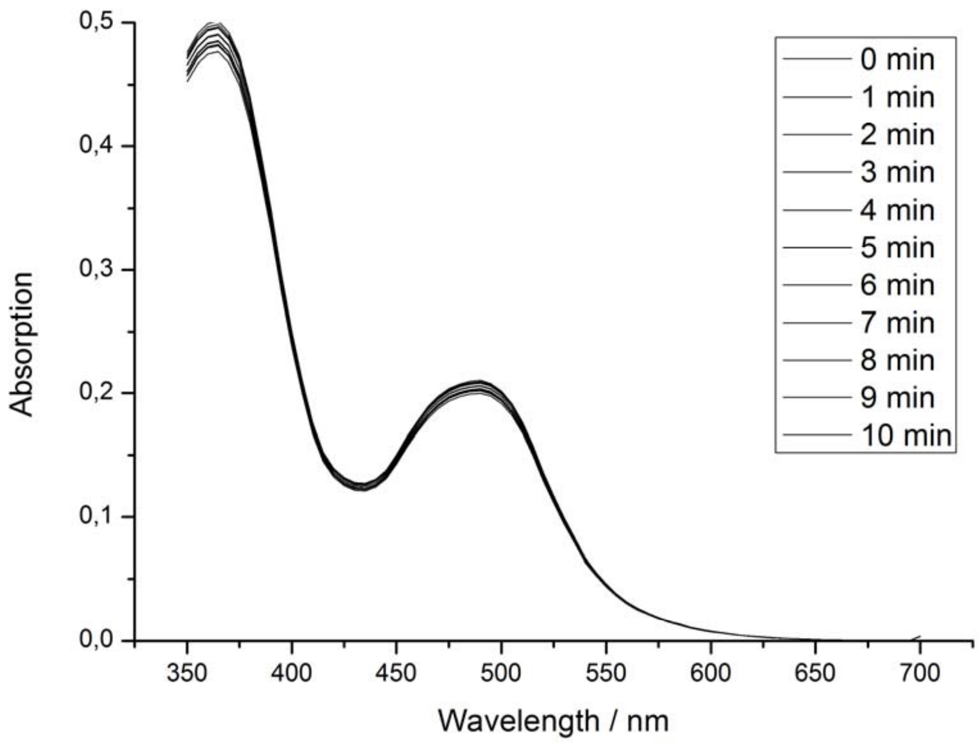

Figure 56. Temporal change of the UV/Vis spectra of **3** by irradiation at 450 nm in CH_3CN.

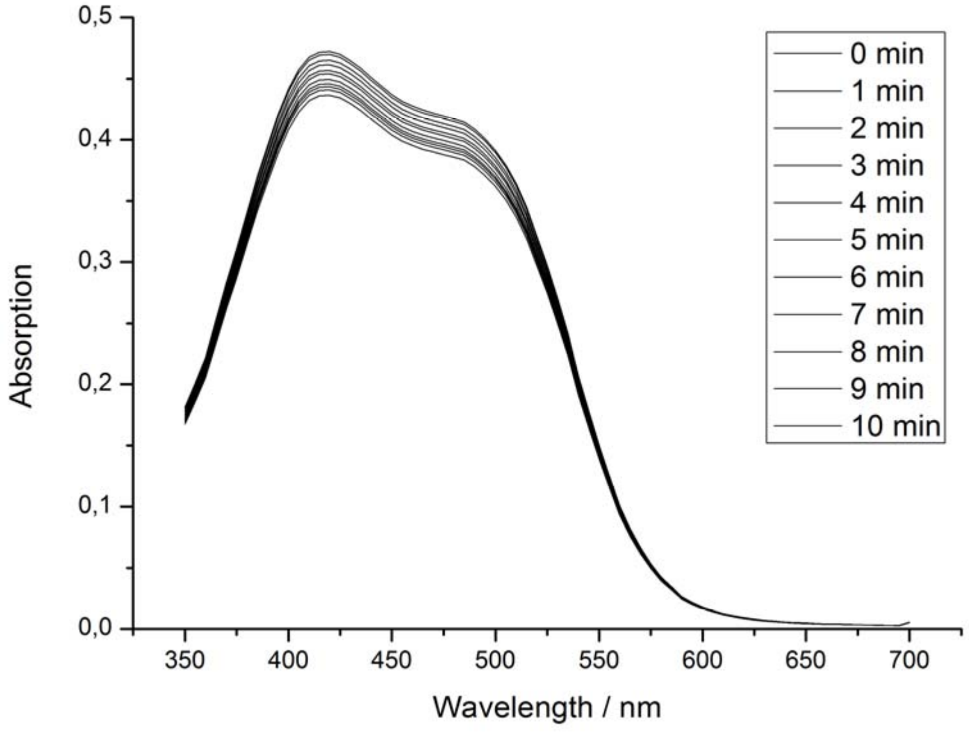

Figure 57. Temporal change of the UV/Vis spectra of **4** by irradiation at 450 nm in CH_3CN.

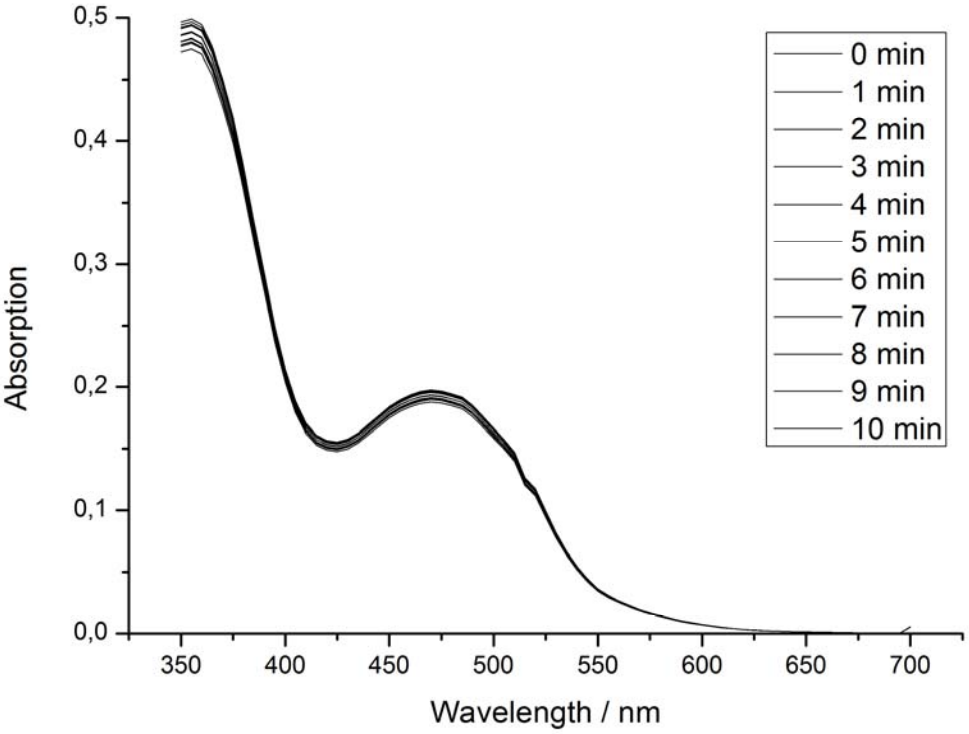

Figure 58. Temporal change of the UV/Vis spectra of **5** by irradiation at 450 nm in CH_3CN.

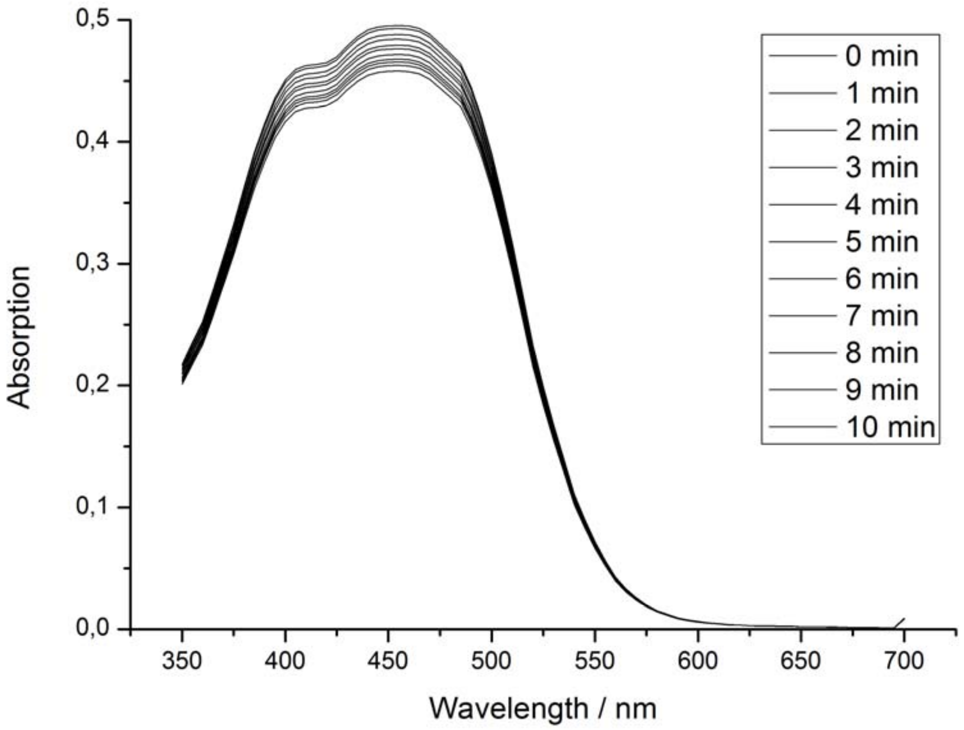

Figure 59. Temporal change of the UV/Vis spectra of **6** by irradiation at 450 nm in CH_3CN.

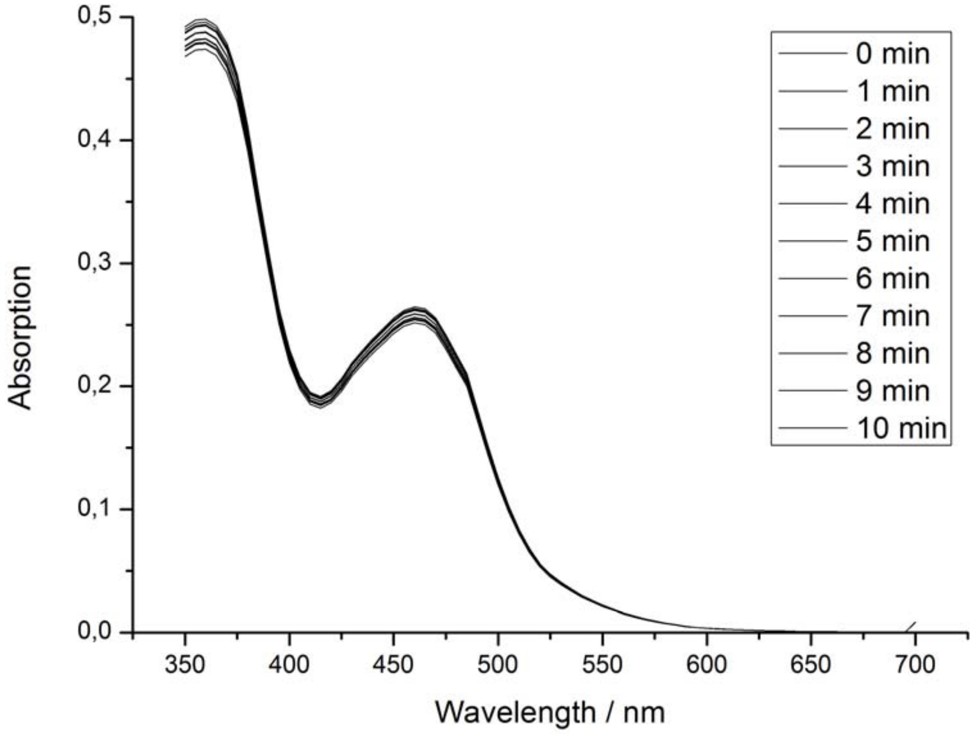

Figure 60. Temporal change of the UV/Vis spectra of **7** by irradiation at 450 nm in CH_3CN.

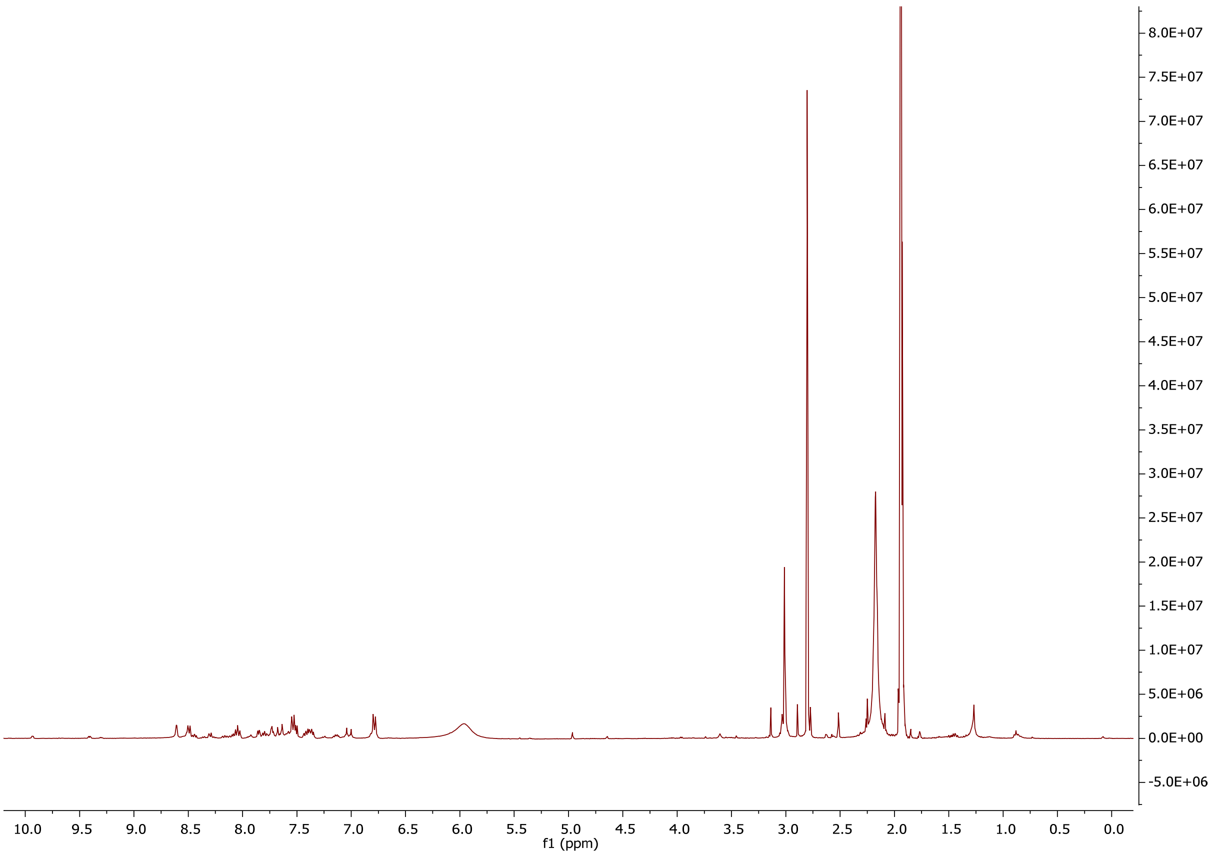

Figure 61. Change of the NMR spectrum of **2** upon irradiation at 450 nm in CH_3CN.

Table 7. Distribution coefficient for compounds **1**-**7**. Average of three independent measurements.

	**log*P***
**1**	+2.6 ± 0.4
**2**	+2.7 ± 0.4
**3**	+2.3 ± 0.3
**4**	+1.7 ± 0.3
**5**	+1.4 ± 0.2
**6**	+0.7 ± 0.2
**7**	+0.5 ± 0.2

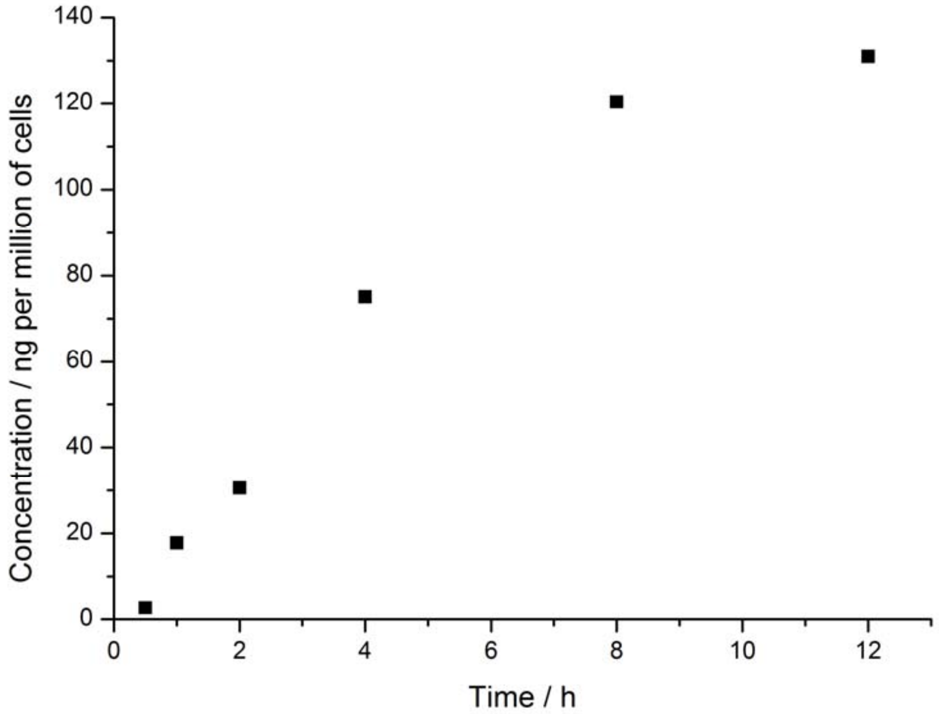

Figure 62. Time dependent cellular uptake of **1** in HeLa cells.

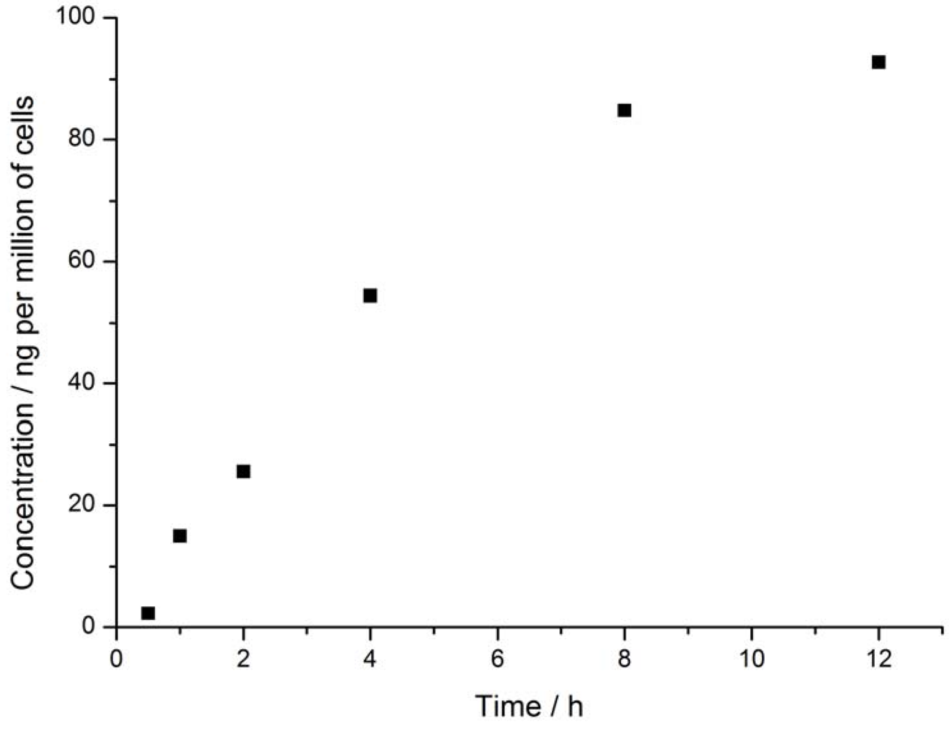

Figure 63. Time dependent cellular uptake of **2** in HeLa cells.

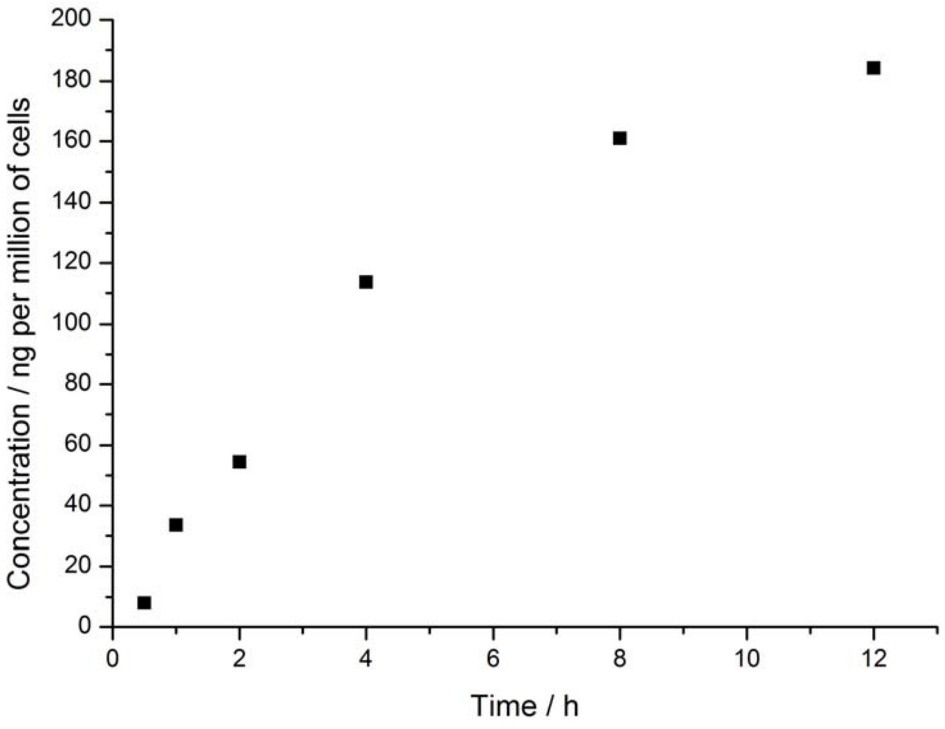

Figure 64. Time dependent cellular uptake of **3** in HeLa cells.

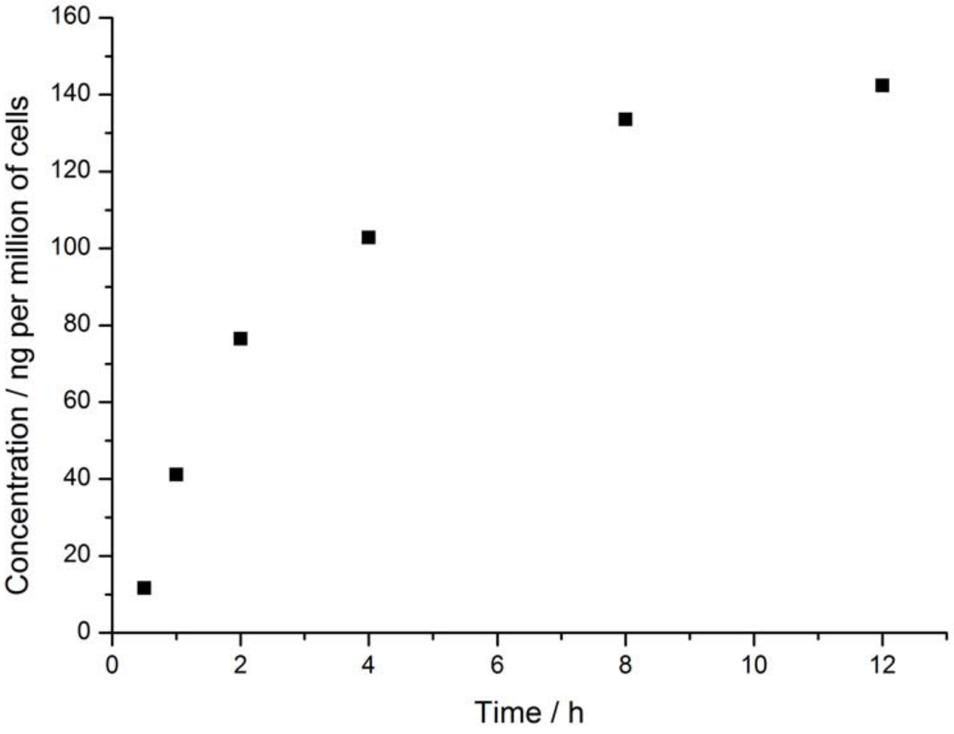

Figure 65. Time dependent cellular uptake of **4** in HeLa cells.

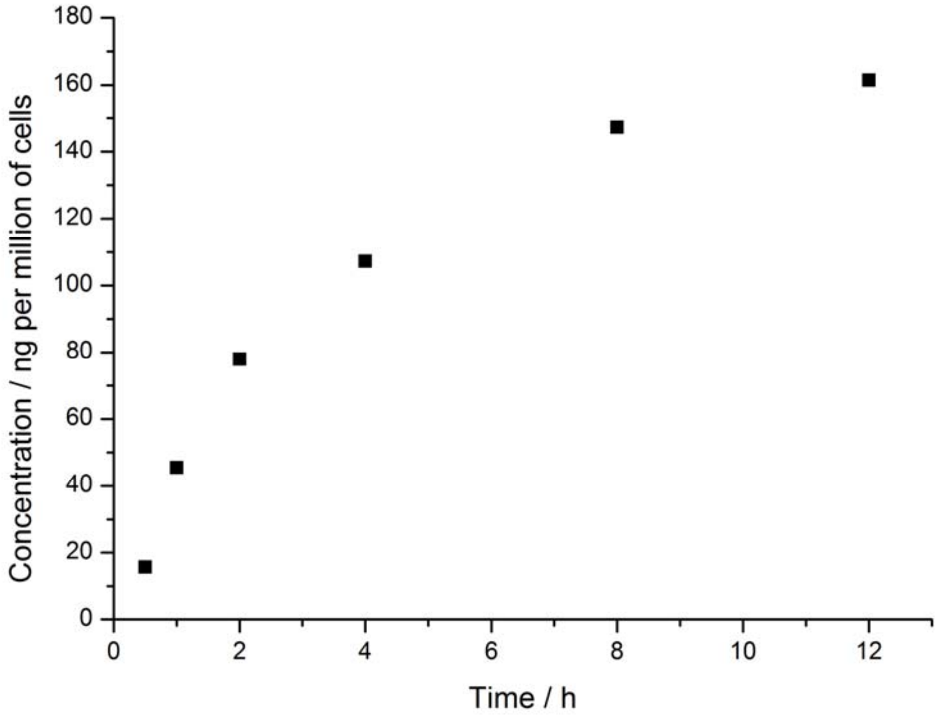

Figure 66. Time dependent cellular uptake of **5** in HeLa cells.

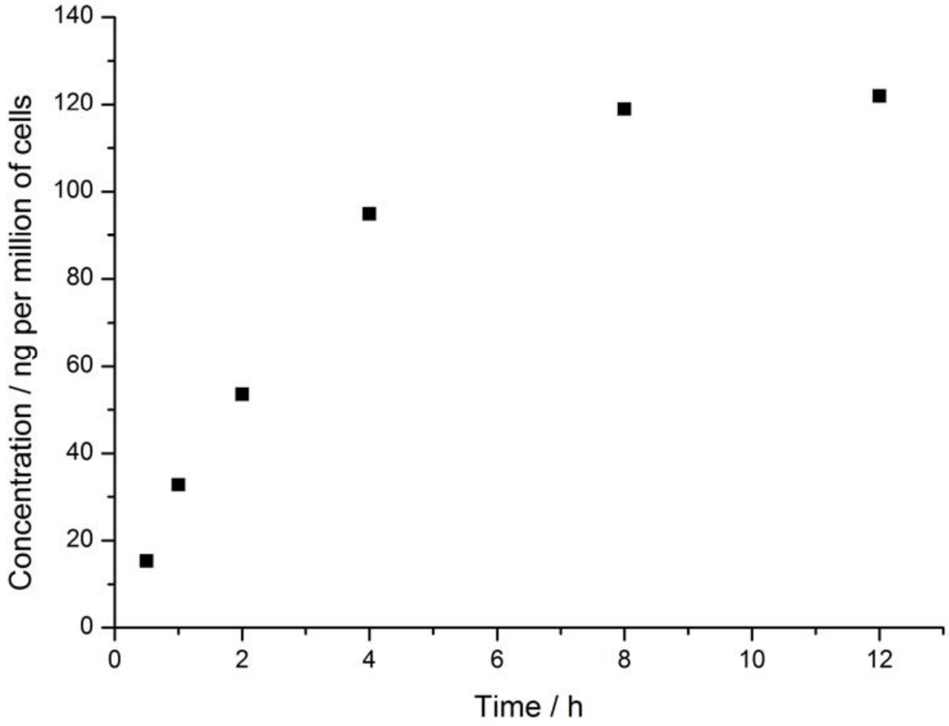

Figure 67. Time dependent cellular uptake of **6** in HeLa cells.

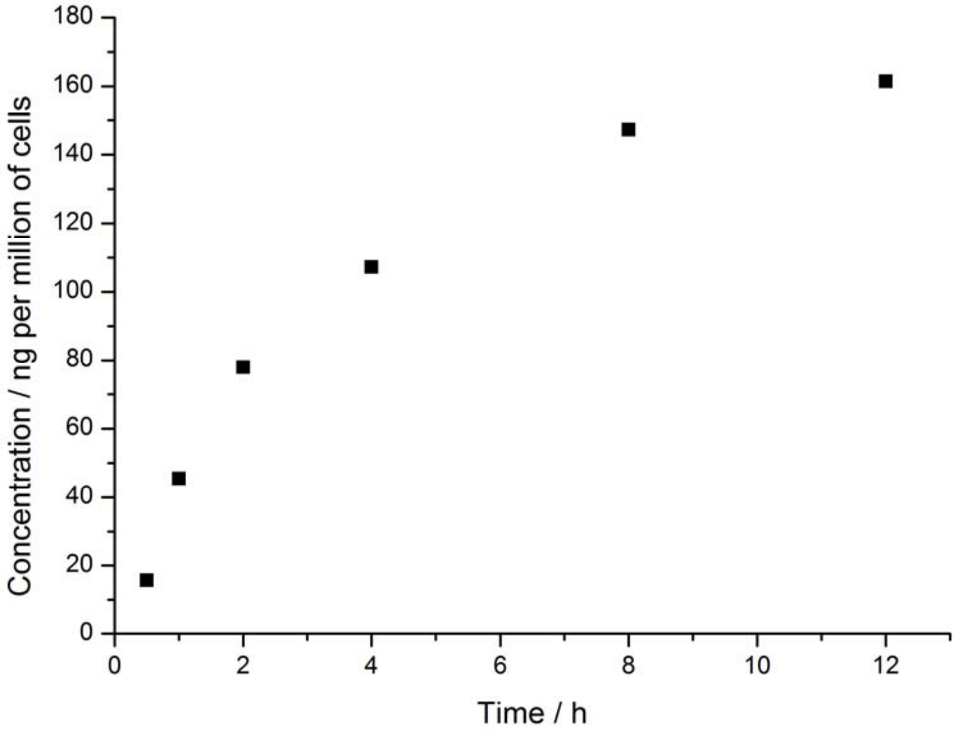

Figure 68. Time dependent cellular uptake of **7** in HeLa cells.

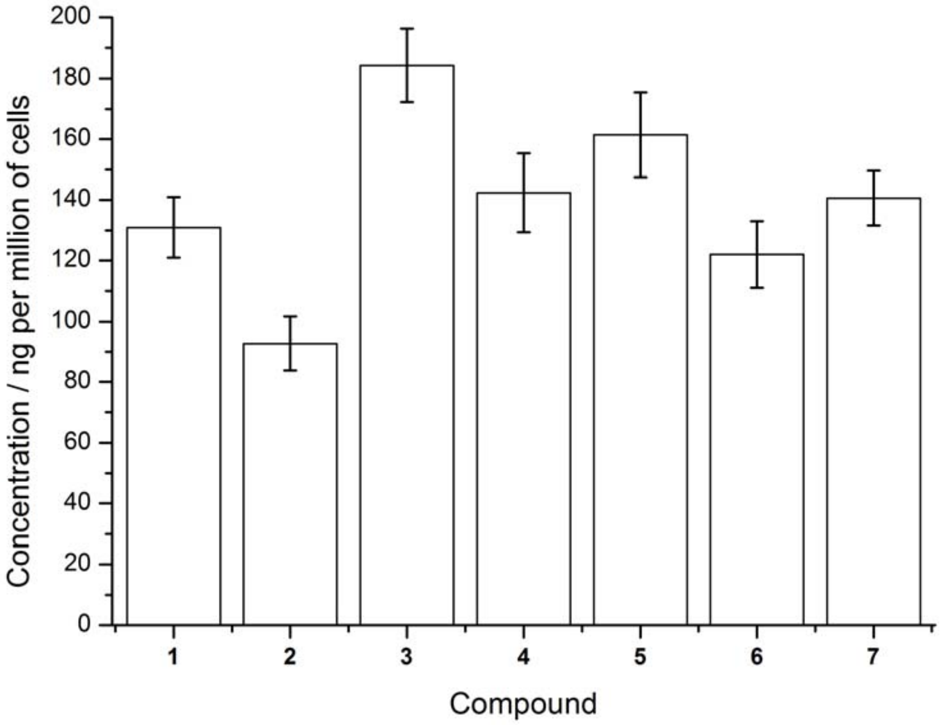

Figure 69. Comparison of uptake of **1**-**7** in HeLa cells. The error bars correspond to the standard deviation of the three replicates.

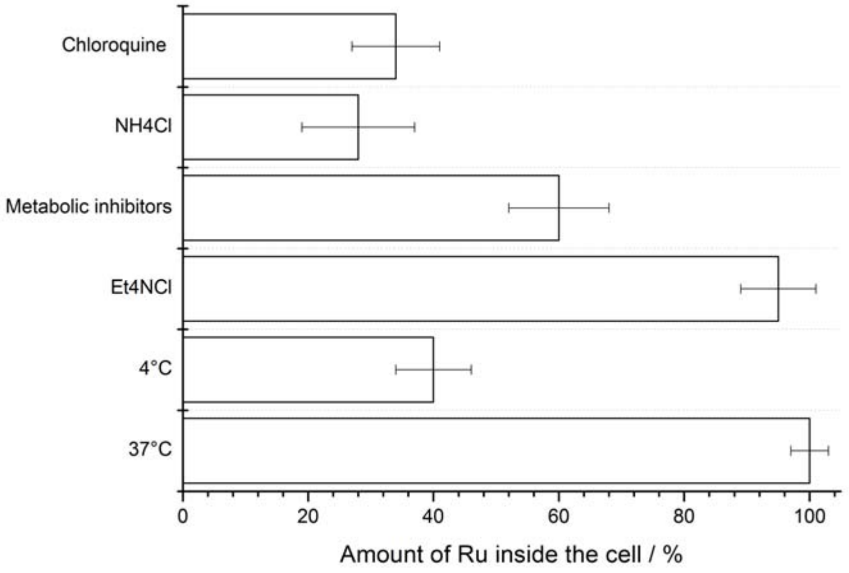

Figure 70. Cell uptake mechanism study of **1** (10 μM, 2% DMSO, *v*%) in the presence of different inhibitors/conditions. Endocytic inhibition: NH_4Cl (50 mM) or chloroquine (100 μM), metabolic inhibition: 2-Deoxy-*D*-glucose (50 mM) and oligomycin (5 μM), cation transporter inhibition: Et_4NCl, low temperature: incubation at 4°C, control: incubation at 37°C. The error bars correspond to the standard deviation of the three replicates.

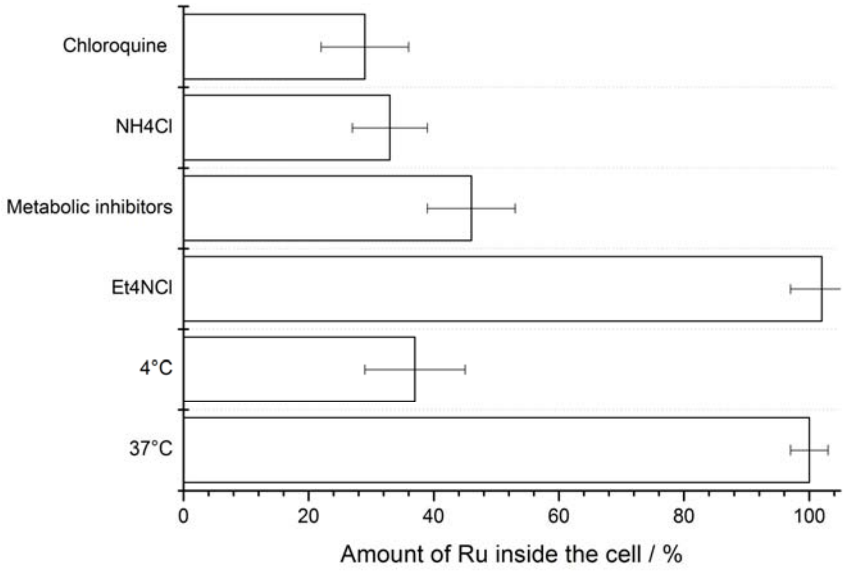

Figure 71. Cell uptake mechanism study of **2** (10 μM, 2% DMSO, *v*%) in the presence of different inhibitors/conditions. Endocytic inhibition: NH_4Cl (50 mM) or chloroquine (100 μM), metabolic inhibition: 2-Deoxy-*D*-glucose (50 mM) and oligomycin (5 μM), cation transporter inhibition: Et_4NCl, low temperature: incubation at 4°C, control: incubation at 37°C. The error bars correspond to the standard deviation of the three replicates.

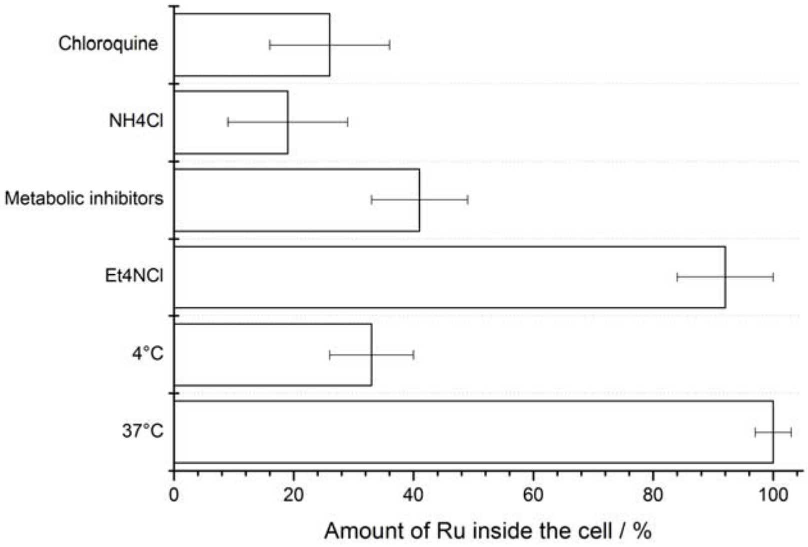

Figure 72. Cell uptake mechanism study of **3** (10 μM, 2% DMSO, *v*%) in the presence of different inhibitors/conditions. Endocytic inhibition: NH_4Cl (50 mM) or chloroquine (100 μM), metabolic inhibition: 2-Deoxy-*D*-glucose (50 mM) and oligomycin (5 μM), cation transporter inhibition: Et_4NCl, low temperature: incubation at 4°C, control: incubation at 37°C. The error bars correspond to the standard deviation of the three replicates.

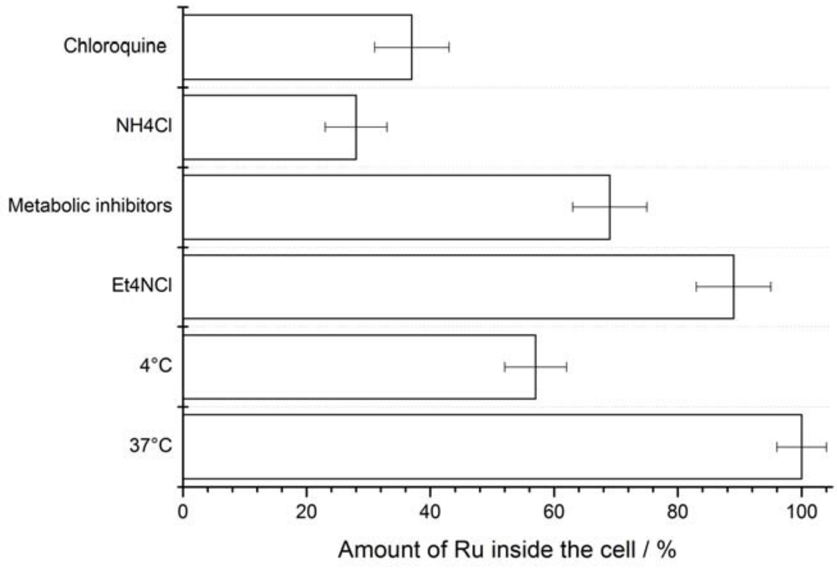

Figure 73. Cell uptake mechanism study of **4** (10 µM, 2% DMSO, *v*%) in the presence of different inhibitors/conditions. Endocytic inhibition: NH_4Cl (50 mM) or chloroquine (100 µM), metabolic inhibition: 2-Deoxy-*D*-glucose (50 mM) and oligomycin (5 µM), cation transporter inhibition: Et_4NCl, low temperature: incubation at 4°C, control: incubation at 37°C. The error bars correspond to the standard deviation of the three replicates.

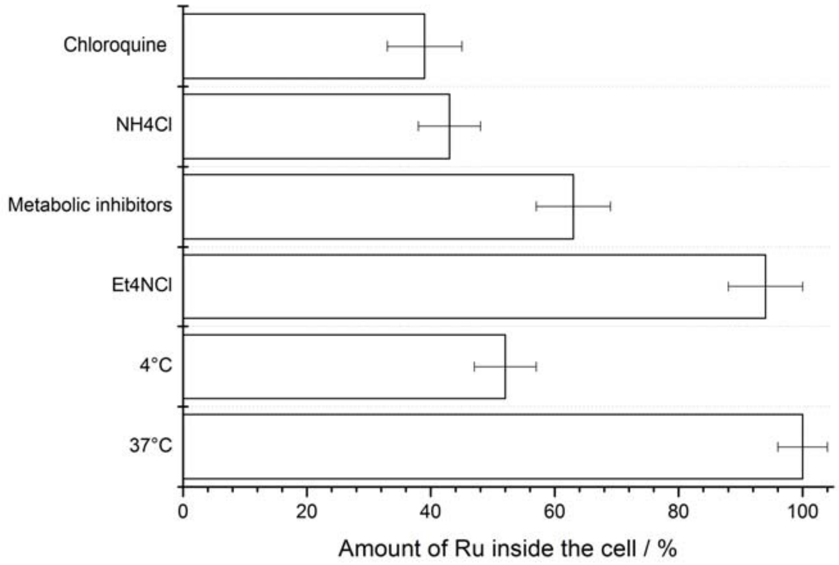

Figure 74. Cell uptake mechanism study of **5** (10 µM, 2% DMSO, *v*%) in the presence of different inhibitors/conditions. Endocytic inhibition: NH_4Cl (50 mM) or chloroquine (100 µM), metabolic inhibition: 2-Deoxy-*D*-glucose (50 mM) and oligomycin (5 µM), cation transporter inhibition: Et_4NCl, low temperature: incubation at 4°C, control: incubation at 37°C. The error bars correspond to the standard deviation of the three replicates.

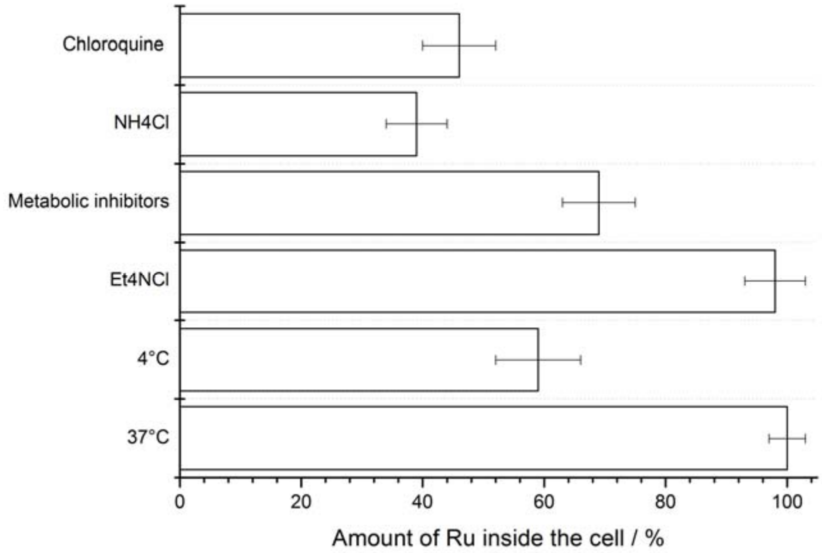

Figure 75. Cell uptake mechanism study of **6** (10 µM, 2% DMSO, *v*%) in the presence of different inhibitors/conditions. Endocytic inhibition: NH_4Cl (50 mM) or chloroquine (100 µM), metabolic inhibition: 2-Deoxy-*D*-glucose (50 mM) and oligomycin (5 µM), cation transporter inhibition: Et_4NCl, low temperature: incubation at 4°C, control: incubation at 37°C. The error bars correspond to the standard deviation of the three replicates.

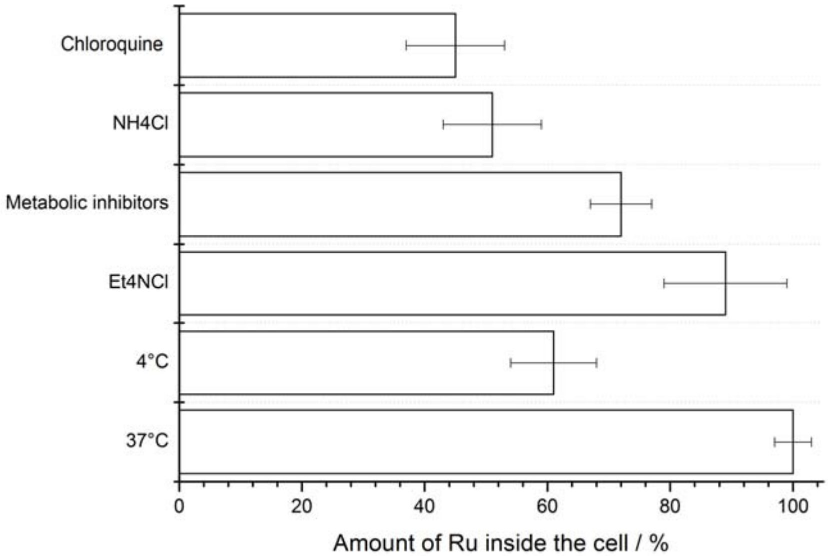

Figure 76. Cell uptake mechanism study of **7** (10 µM, 2% DMSO, *v*%) in the presence of different inhibitors/conditions. Endocytic inhibition: NH_4Cl (50 mM) or chloroquine (100 µM), metabolic inhibition: 2-Deoxy-*D*-glucose (50 mM) and oligomycin (5 µM), cation transporter inhibition: Et_4NCl, low temperature: incubation at 4°C, control: incubation at 37°C. The error bars correspond to the standard deviation of the three replicates.

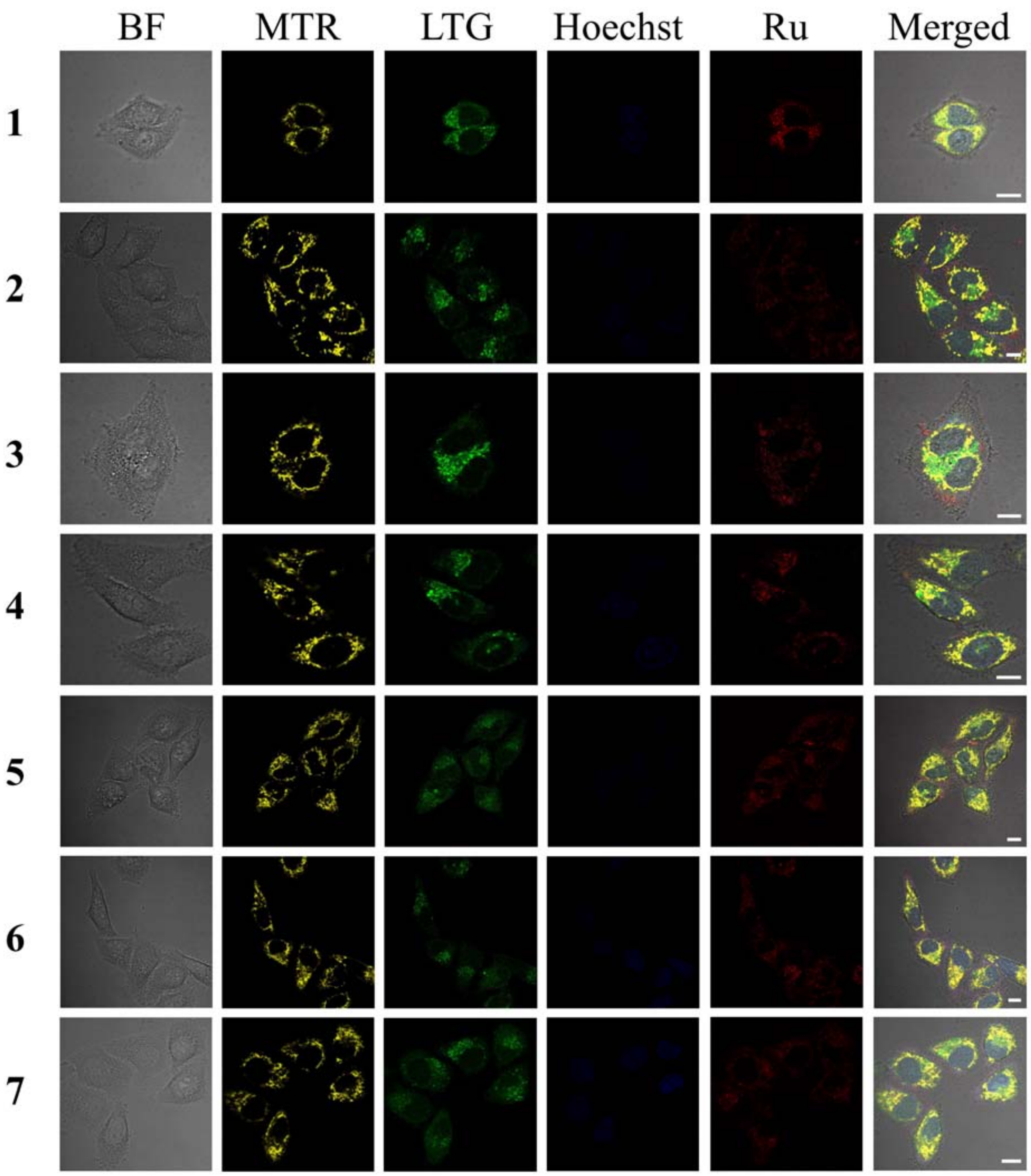

Figure 77. Confocal luminescence image of HeLa cells incubated with the compounds **1-7** (10 μM, 2% DMSO, *v*%) for 8 h at 37°C in the dark. Then, the cells were incubated with the organelle trackers MitoTracker Deep Red (MTR, 500 nM, λ_{ex} = 633 nm, λ_{em} = 650 - 720 nm), LysoTracker Green (LTG, 500 nM, λ_{ex} = 488 nm, λ_{em} = 490 - 550 nm) and Hoechst 33342 (Hoechst, 5 μg mL^{-1}, λ_{ex} = 405 nm, λ_{em} = 410 - 470 nm) for 30 min. The investigated Ru complexes were detected using their 1-Photon luminescence (Ru, λ_{ex} = 458 nm, λ_{em} = 600 - 750 nm) properties. The scale bar represents a length of 20 μm.

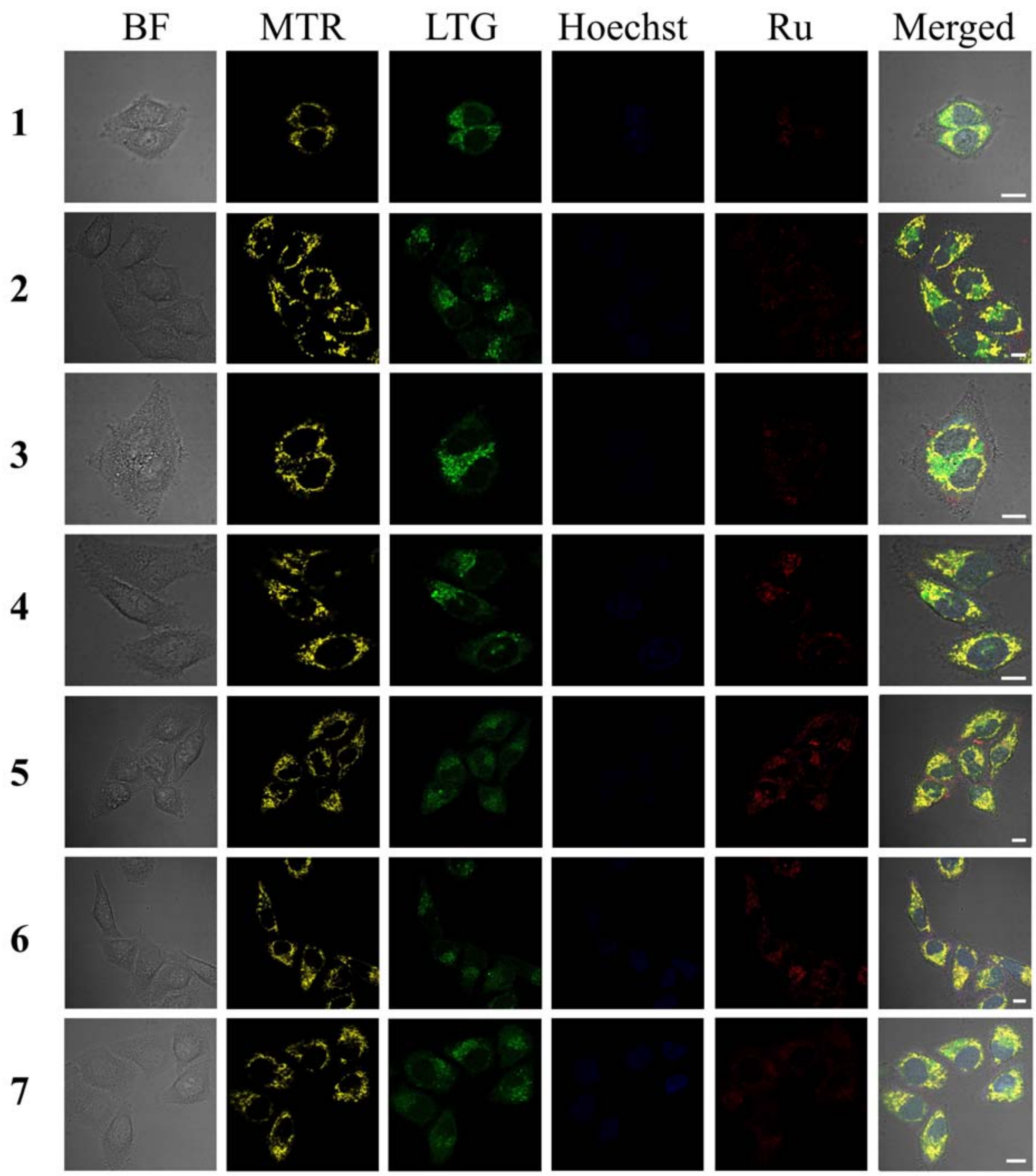

Figure 78. Confocal luminescence image of HeLa cells incubated with the compounds **1**-**7** (10 μM, 2% DMSO, *v*%) for 8 h at 37°C in the dark. Then, the cells were incubated with the organelle trackers MitoTracker Deep Red (MTR, 500 nM, λ_{ex} = 633 nm, λ_{em} = 650 - 720 nm), LysoTracker Green (LTG, 500 nM, λ_{ex} = 488 nm, λ_{em} = 490 - 550 nm) and Hoechst 33342 (Hoechst, 5 μg mL^{-1}, λ_{ex} = 405 nm, λ_{em} = 410 - 470 nm) for 30 min. The investigated Ru complexes were detected using their 2-Photon luminescence (Ru, λ_{ex} = 800 nm, λ_{em} = 600 - 750 nm) properties. The scale bar represents a length of 20 μm.

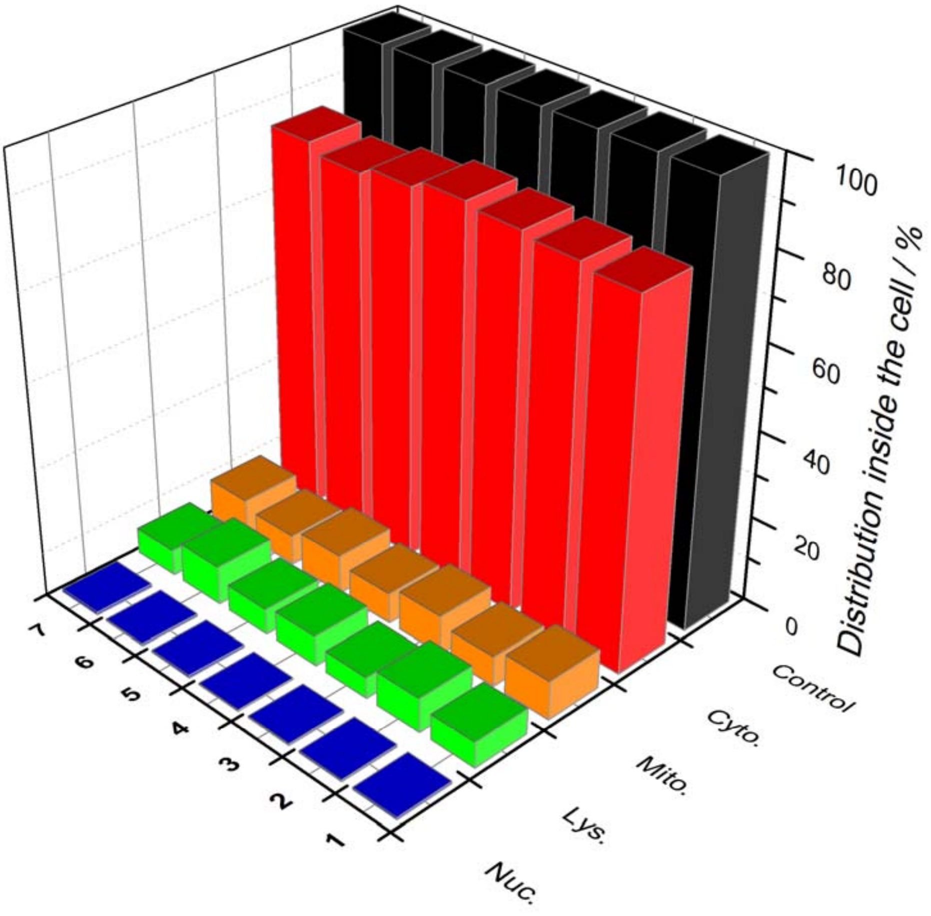

Figure 79. Cellular distribution (Cyto. = Cytoplasm, Mito. = Mitochondria, Lys. = Lysosome, Nuc. = Nucleus) of **1**-**7** (10 μM, 2% DMSO, *v*%) in HeLa cells after 8 h incubation in the dark determined via ICP-MS. The error bars correspond to the standard deviation of the three replicates: Nuc.: 0.3-0.5%, Lys.: 0.9-2.1%, Mito.: 3.2-5.2%, Cyto.: 5.5%-8.3%, Control: 4.6-7.9%.

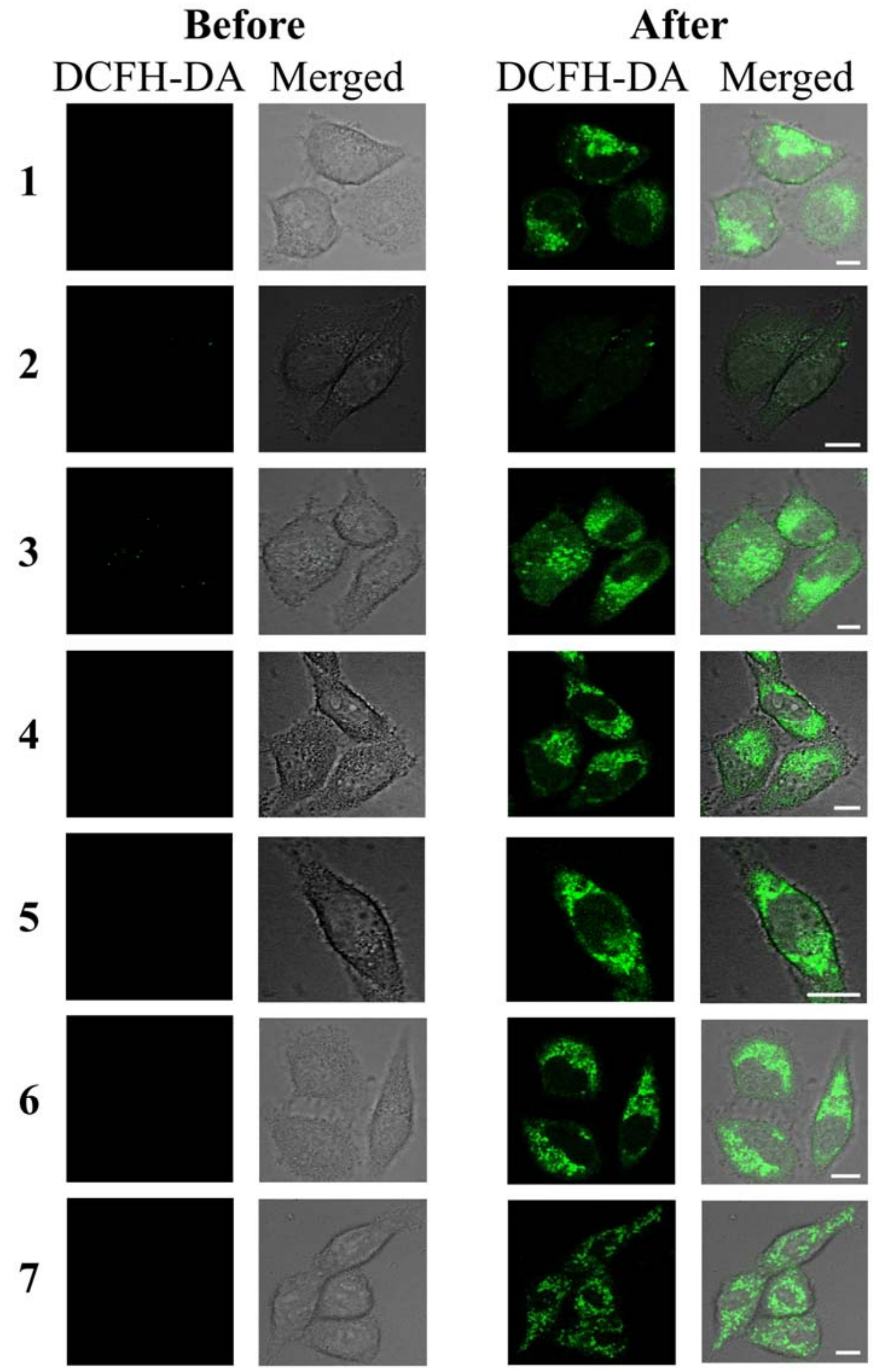

Figure 80. Confocal luminescence image of HeLa cells incubated with the compounds **1-7** (10 µM, 2% DMSO, *v*%) for 4 h at 37°C in the dark and after that with DCFH-DA (λ_{ex} = 488 nm, λ_{em} = 510 - 550 nm) for 30 min at 37°C in the dark before and after 1-Photon irradiation (λ_{ex} = 488 nm). The scale bar represents a length of 20 µm.

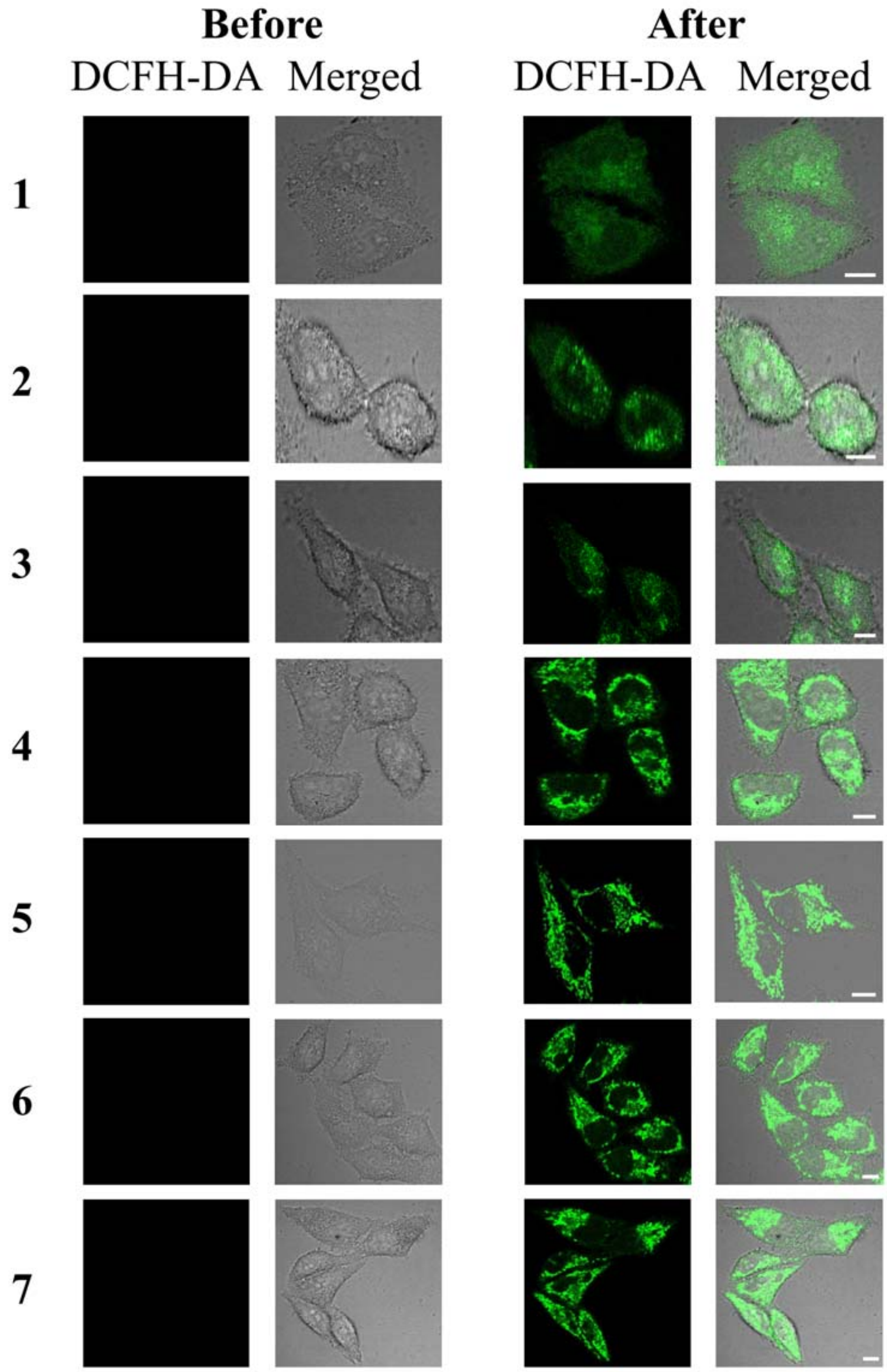

Figure 81. Confocal luminescence image of HeLa cells incubated with the compounds **1-7** (10 μM, 2% DMSO, *v*%) for 4 h at 37°C in the dark and after that with DCFH-DA (λ_{ex} = 488 nm, λ_{em} = 510 - 550 nm) for 30 min at 37°C in the dark before and after 2-Photon irradiation (λ_{ex} = 800 nm). The scale bar represents a length of 20 μm.

Table 8. IC_{50} values in the dark and upon irradiation at 480 and 540 nm for **1-7** in comparison to cisplatin and Protoporphyrin IX (PpIX) in non-cancerous retinal pigment epithelium (RPE-1) and human cervical carcinoma (HeLa) cells. Average of three independent measurements.

	**RPE-1**					**HeLa**				
	**dark**	**480 nm (10 min, 3.1 J cm^{-2})**	**PI**	**540 nm (40 min, 9.5 J cm^{-2})**	**PI**	**dark**	**480 nm (10 min, 3.1 J cm^{-2})**	**PI**	**540 nm (40 min, 9.5 J cm^{-2})**	**PI**
**1**[a)]	>100	30.9 ± 0.8	>3.2	31.7 ± 1.2	>3.2	>100	29.5 ± 1.2	>3.4	48.7 ± 4.5	>2.1
**2**[a)]	>100	38.4 ± 8.1	>2.6	49.0 ± 6.2	>2.0	>100	29.4 ± 8.3	>3.4	52.3 ± 6.1	>1.9
**3**[a)]	>100	53.6 ± 3.2	>1.9	44.9 ± 2.9	>2.2	>100	15.3 ± 1.4	>6.5	11.3 ± 2.2	>8.8
**4**	>100	10.9 ± 2.7	>9.2	14.7 ± 3.1	>6.8	>100	8.5 ± 1.2	>11.8	10.6 ± 2.2	>9.4
**5**	>100	10.2 ± 2.1	>9.8	12.7 ± 2.9	>7.9	>100	6.4 ± 1.7	>16.7	7.9 ± 1.3	>12.7
**6**	>100	7.3 ± 1.2	>13.7	8.1 ± 1.6	>12.3	>100	5.3 ± 0.6	>18.9	5.8 ± 0.9	>17.2
**7**	>100	3.1 ± 0.8	>32.3	8.4 ± 1.3	>11.9	>100	1.2 ± 0.4	>83.3	1.5 ± 0.5	>66.7
**PpIX**	>100	3.8 ± 0.1	>26.3	3.3 ± 0.1	>30.3	>100	2.5 ± 0.1	>40.0	2.1 ± 0.3	>47.6
**cisplatin**	29.3 ± 1.4	-	-	-	-	10.5 ± 0.8	-	-	-	-

[a)] due to solubility limitations the compounds were investigated as chloride salts.

Table 9. IC_{50} values in the dark and upon irradiation at 480 and 540 nm for **1-7** in comparison to cisplatin and Protoporphyrin IX (PpIX) in mouse colon carcinoma (CT-26) and human glioblastoma astrocytoma (U373) cells. Average of three independent measurements.

	CT-26					U373				
	**dark**	**480 nm (10 min, 3.1 J cm^{-2})**	**PI**	**540 nm (40 min, 9.5 J cm^{-2})**	**PI**	**dark**	**480 nm (10 min, 3.1 J cm^{-2})**	**PI**	**540 nm (40 min, 9.5 J cm^{-2})**	**PI**
**1**[a)]	>100	20.3 ± 1.8	>4.9	33.4 ± 3.5	>3.0	>100	51.7 ± 3.2	>1.9	83.1 ± 6.7	>1.2
**2**[a)]	>100	42.6 ± 3.8	>2.3	62.5 ± 8.2	>1.6	>100	49.1 ± 6.0	>2.0	61.4 ± 5.6	>1.6
**3**[a)]	>100	19.3 ± 2.1	>5.2	23.0 ± 2.6	>4.3	>100	41.0 ± 3.5	>2.4	51.3 ± 3.4	>1.9
**4**	>100	7.3 ± 1.4	>13.7	9.6 ± 1.9	>10.4	>100	14.3 ± 2.4	>7.0	19.0 ± 2.8	>5.3
**5**	>100	5.1 ± 1.1	>19.6	6.2 ± 1.0	>16.1	>100	15.3 ± 2.2	>6.5	17.8 ± 3.2	>5.6
**6**	>100	2.4 ± 0.5	>41.7	3.1 ± 0.3	>32.3	>100	8.3 ± 1.1	>12.0	10.7 ± 1.9	>9.3
**7**	>100	0.7 ± 0.4	>142.9	0.9 ± 0.3	>111.1	>100	10.5 ± 1.7	>9.5	13.5 ± 1.6	>7.4
**PpIX**	>100	3.5 ± 0.4	>28.6	4.1 ± 0.6	>24.4	>100	3.3 ± 0.5	>30.3	3.6 ± 0.4	>27.8
**cisplatin**	6.5 ± 1.1	-	-	-	-	17.6 ± 1.7	-	-	-	-

[a)] due to solubility limitations the compounds were investigated as chloride salts.

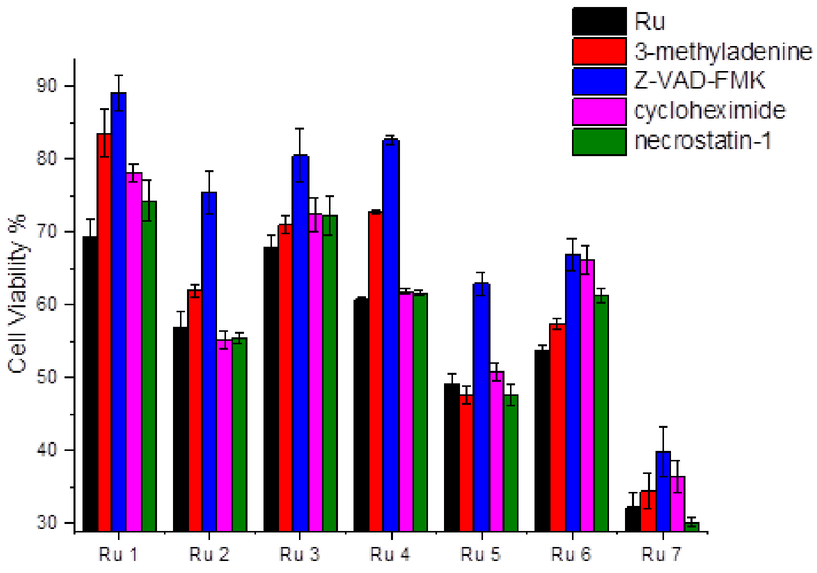

Figure 82. Cell death mechanism study. Z-VAD-fmk (10 µM), 3-Methyladenine (100 µM), Cycloheximide (0.1 µM), Leupeptin (100 µM), Necrostatin-1 (60 µM) were pre-incubated for 40 min. The compounds (5 μM) were then incubated for 8h before the irradiation at 500 nm. The error bars correspond to the standard deviation of the three replicates.

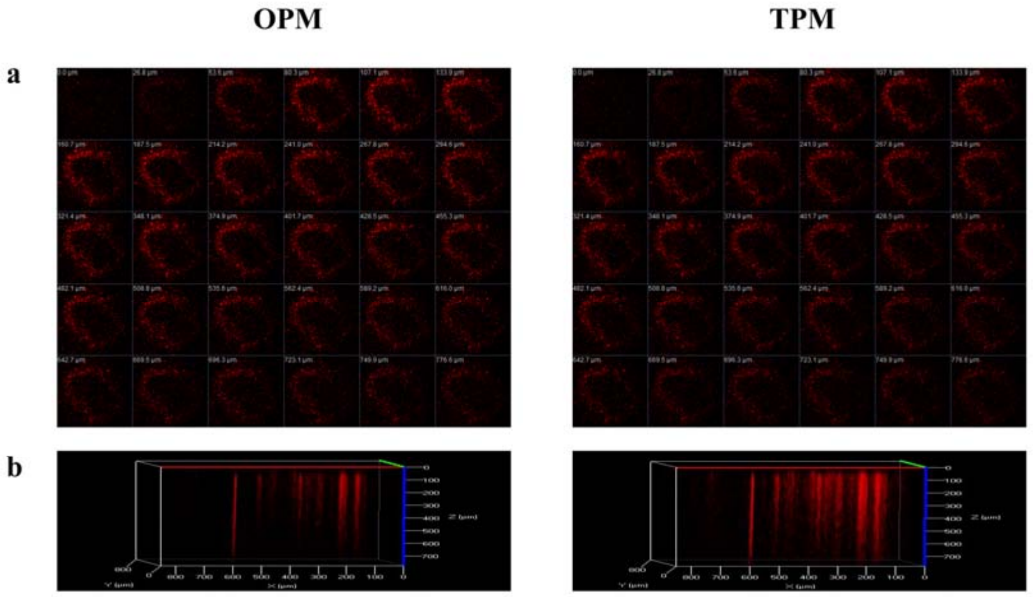

Figure 83. 1- (OPM, λ_{ex} = 458 nm, λ_{em} = 600 – 750 nm) and 2-Photon (TPM, λ_{ex} = 800 nm, λ_{em} = 600 - 750 nm) excited Z-stack images in HeLa MCTS after incubation of **1** after 12 h (20 µM, 2% DMSO, *v*%). **a)** Z-axis images scanning from the top to the bottom of an intact spheroid. **b)** 3D z-stack of an intact spheroid.

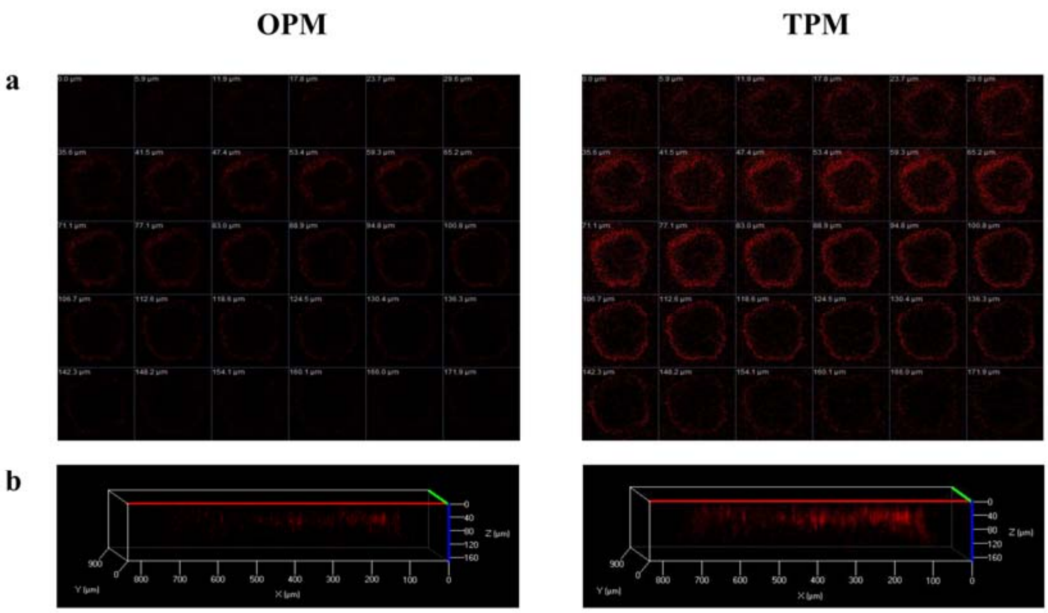

Figure 84. 1- (OPM, λ_{ex} = 458 nm, λ_{em} = 600 – 750 nm) and 2-Photon (TPM, λ_{ex} = 800 nm, λ_{em} = 600 - 750 nm) excited Z-stack images in HeLa MCTS after incubation of **2** after 12 h (20 µM, 2% DMSO, *v*%). **a)** Z-axis images scanning from the top to the bottom of an intact spheroid. **b)** 3D z-stack of an intact spheroid.

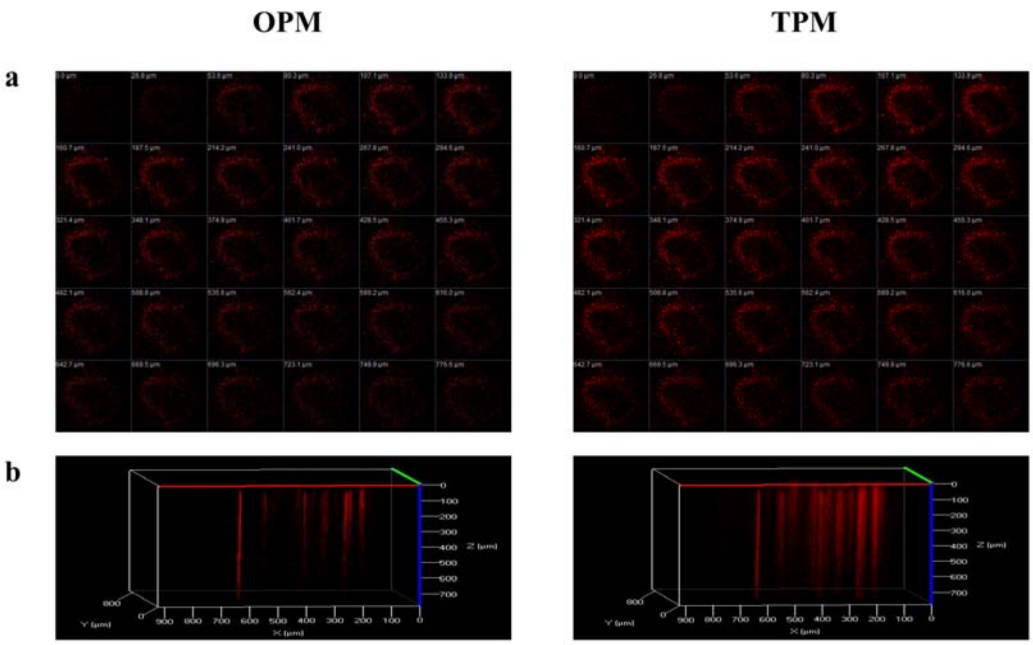

Figure 85. 1- (OPM, λ_{ex} = 458 nm, λ_{em} = 600 – 750 nm) and 2-Photon (TPM, λ_{ex} = 800 nm, λ_{em} = 600 - 750 nm) excited Z-stack images in HeLa MCTS after incubation of **3** after 12 h (20 µM, 2% DMSO, *v*%). **a)** Z-axis images scanning from the top to the bottom of an intact spheroid. **b)** 3D z-stack of an intact spheroid.

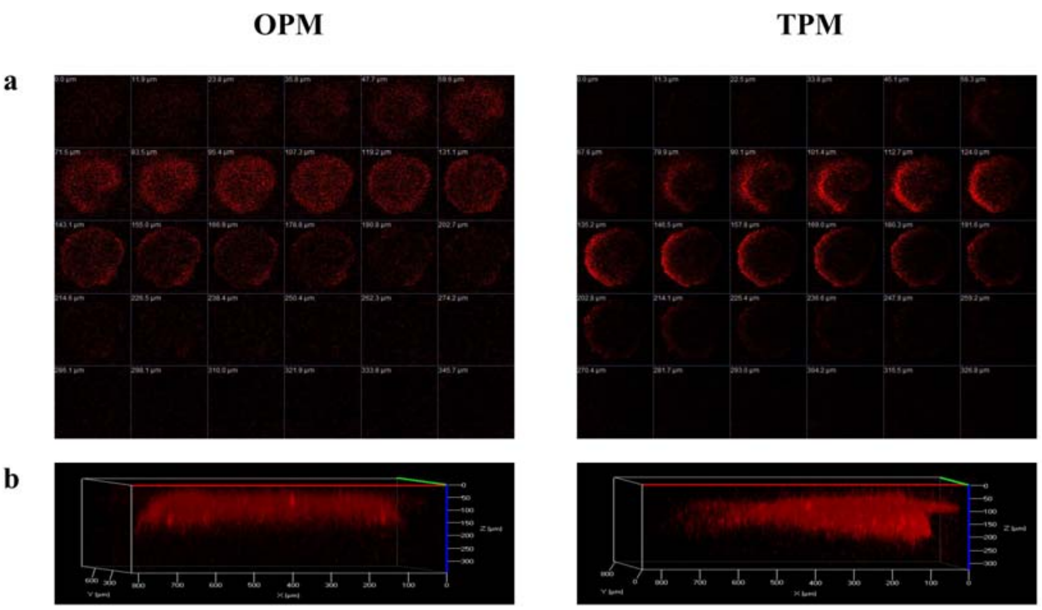

Figure 86. 1- (OPM, λ_{ex} = 458 nm, λ_{em} = 600 – 750 nm) and 2-Photon (TPM, λ_{ex} = 800 nm, λ_{em} = 600 - 750 nm) excited Z-stack images in HeLa MCTS after incubation of **4** after 12 h (20 µM, 2% DMSO, *v*%). **a)** Z-axis images scanning from the top to the bottom of an intact spheroid. **b)** 3D z-stack of an intact spheroid.

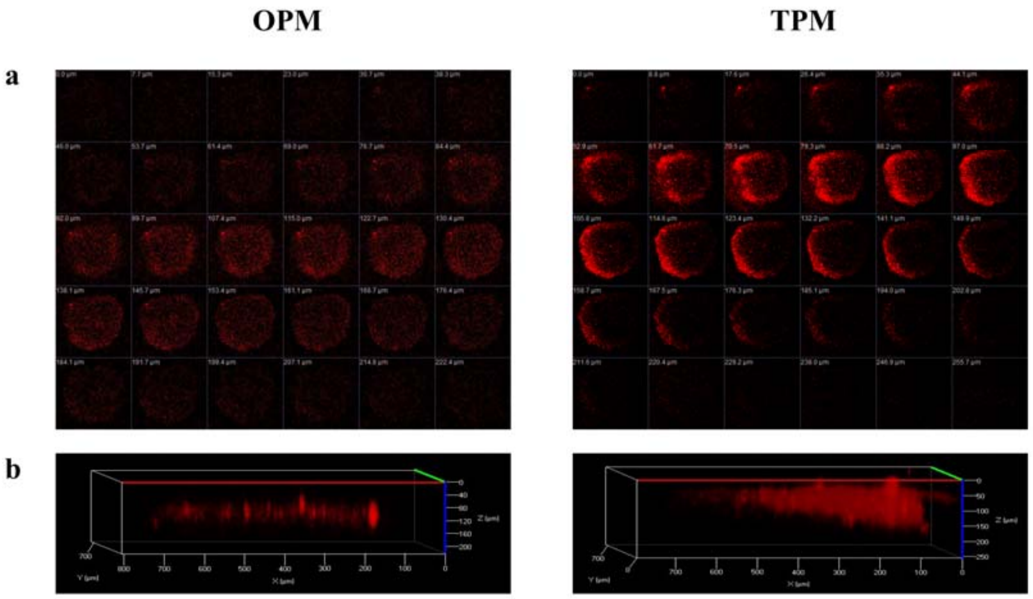

Figure 87. 1- (OPM, λ_{ex} = 458 nm, λ_{em} = 600 – 750 nm) and 2-Photon (TPM, λ_{ex} = 800 nm, λ_{em} = 600 - 750 nm) excited Z-stack images in HeLa MCTS after incubation of **5** after 12 h (20 µM, 2% DMSO, *v*%). **a)** Z-axis images scanning from the top to the bottom of an intact spheroid. **b)** 3D z-stack of an intact spheroid.

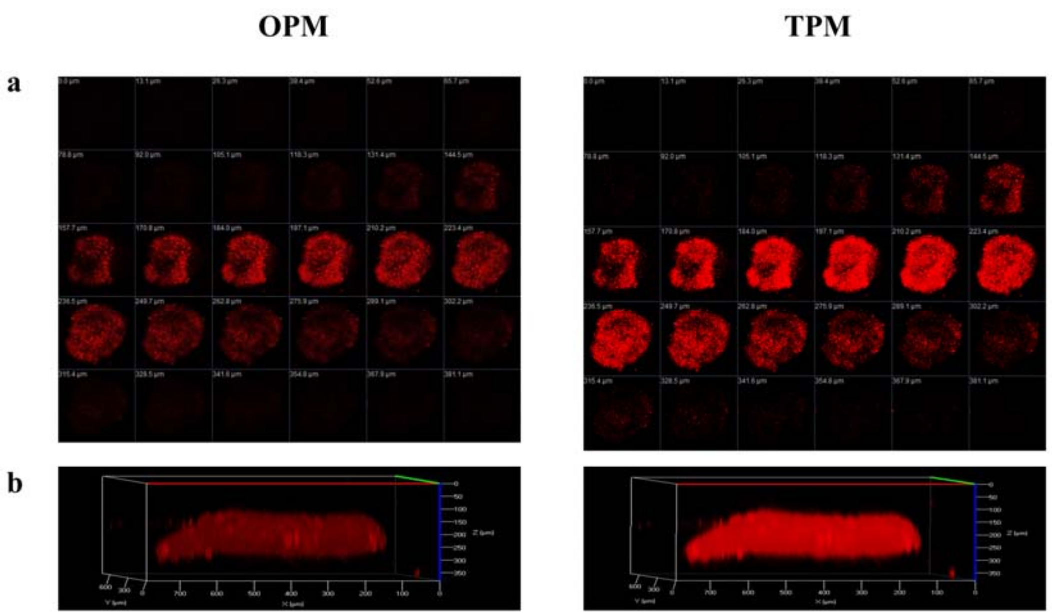

Figure 88. 1- (OPM, λ_{ex} = 458 nm, λ_{em} = 600 – 750 nm) and 2-Photon (TPM, λ_{ex} = 800 nm, λ_{em} = 600 - 750 nm) excited Z-stack images in HeLa MCTS after incubation of **6** after 12 h (20 µM, 2% DMSO, *v*%). **a)** Z-axis images scanning from the top to the bottom of an intact spheroid. **b)** 3D z-stack of an intact spheroid.

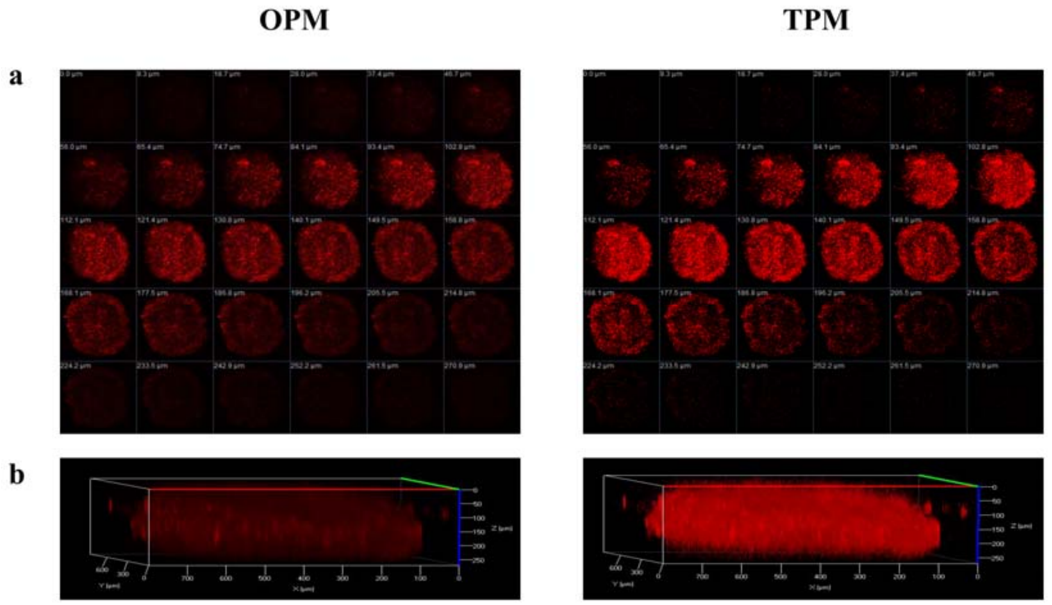

Figure 89. 1- (OPM, λ_{ex} = 458 nm, λ_{em} = 600 – 750 nm) and 2-Photon (TPM, λ_{ex} = 800 nm, λ_{em} = 600 - 750 nm) excited Z-stack images in HeLa MCTS after incubation of **7** after 12 h (20 µM, 2% DMSO, *v*%). **a)** Z-axis images scanning from the top to the bottom of an intact spheroid. **b)** 3D z-stack of an intact spheroid.

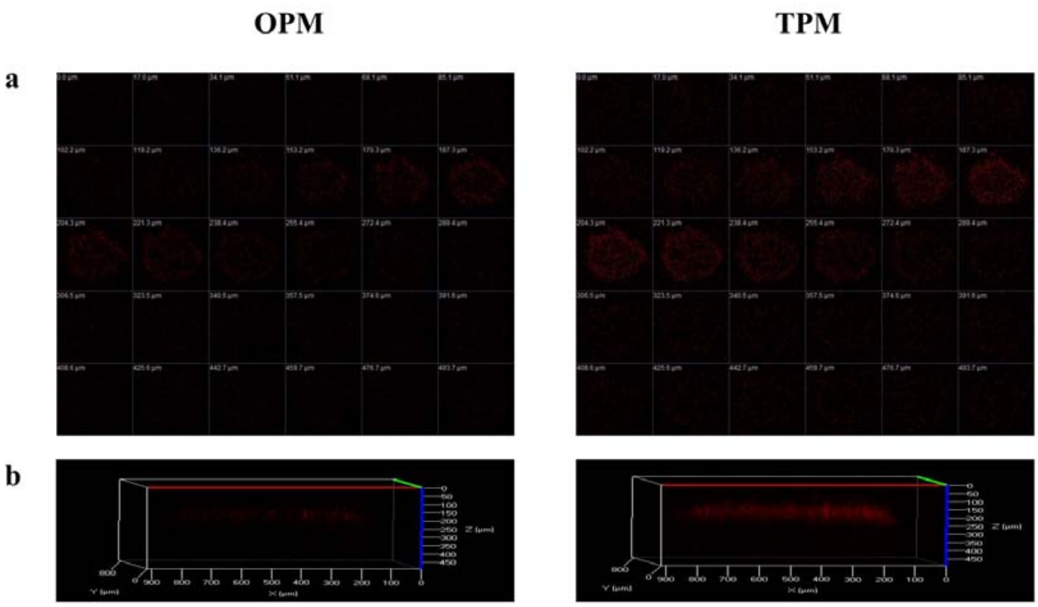

Figure 90. 1- (OPM, λ_{ex} = 458 nm, λ_{em} = 600 – 750 nm) and 2-Photon (TPM, λ_{ex} = 800 nm, λ_{em} = 600 - 750 nm) excited Z-stack images in HeLa MCTS after incubation of **1** after 60 h (20 μM, 2% DMSO, *v*%). **a)** Z-axis images scanning from the top to the bottom of an intact spheroid. **b)** 3D z-stack of an intact spheroid.

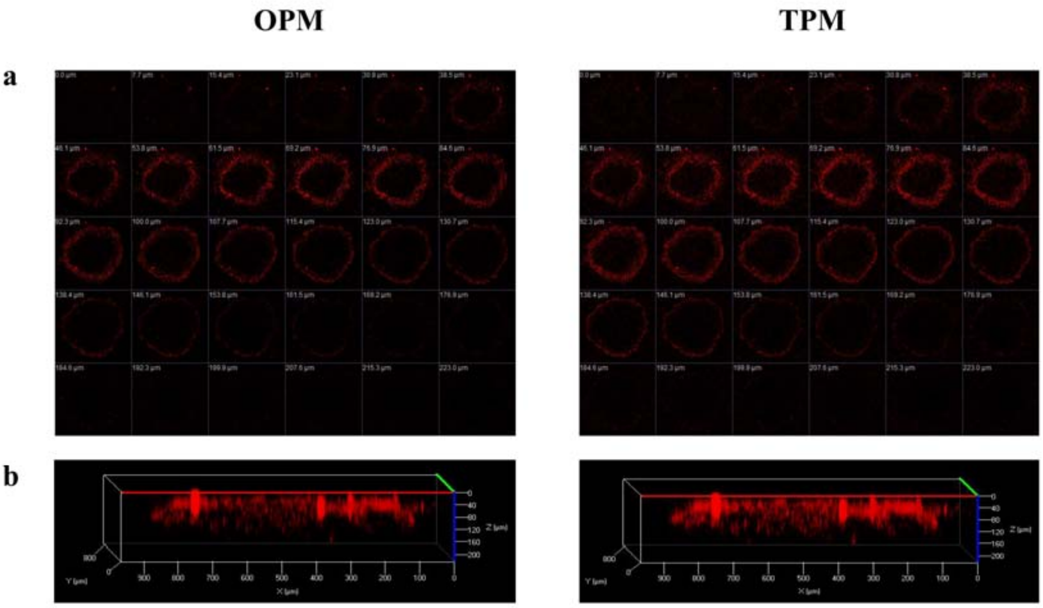

Figure 91. 1- (OPM, λ_{ex} = 458 nm, λ_{em} = 600 – 750 nm) and 2-Photon (TPM, λ_{ex} = 800 nm, λ_{em} = 600 - 750 nm) excited Z-stack images in HeLa MCTS after incubation of **2** after 60 h (20 μM, 2% DMSO, *v*%). **a)** Z-axis images scanning from the top to the bottom of an intact spheroid. **b)** 3D z-stack of an intact spheroid.

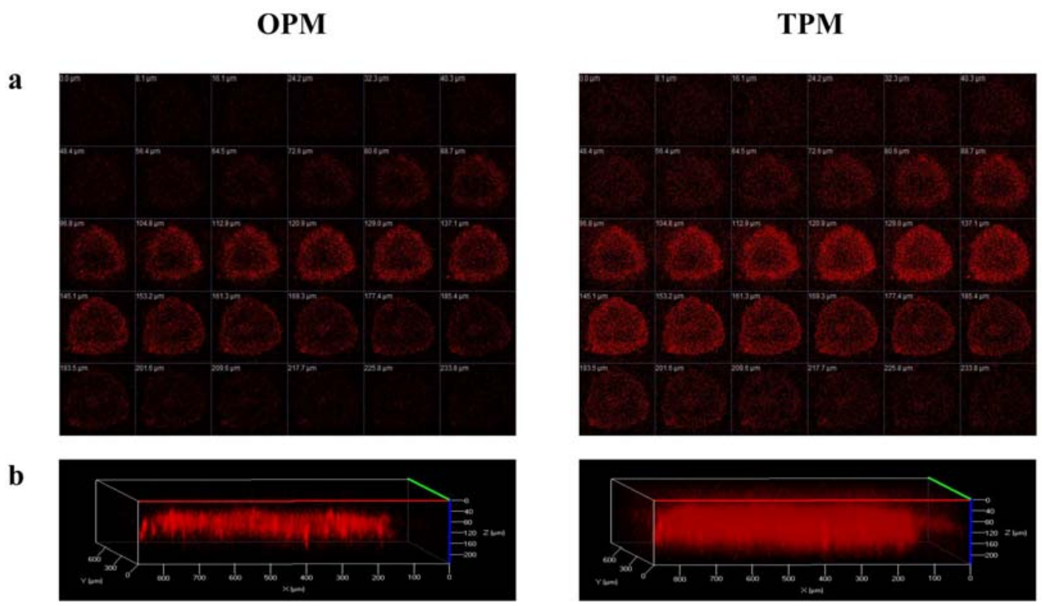

Figure 92. 1- (OPM, λ_{ex} = 458 nm, λ_{em} = 600 – 750 nm) and 2-Photon (TPM, λ_{ex} = 800 nm, λ_{em} = 600 - 750 nm) excited Z-stack images in HeLa MCTS after incubation of **3** after 60 h (20 µM, 2% DMSO, *v*%). **a)** Z-axis images scanning from the top to the bottom of an intact spheroid. **b)** 3D z-stack of an intact spheroid.

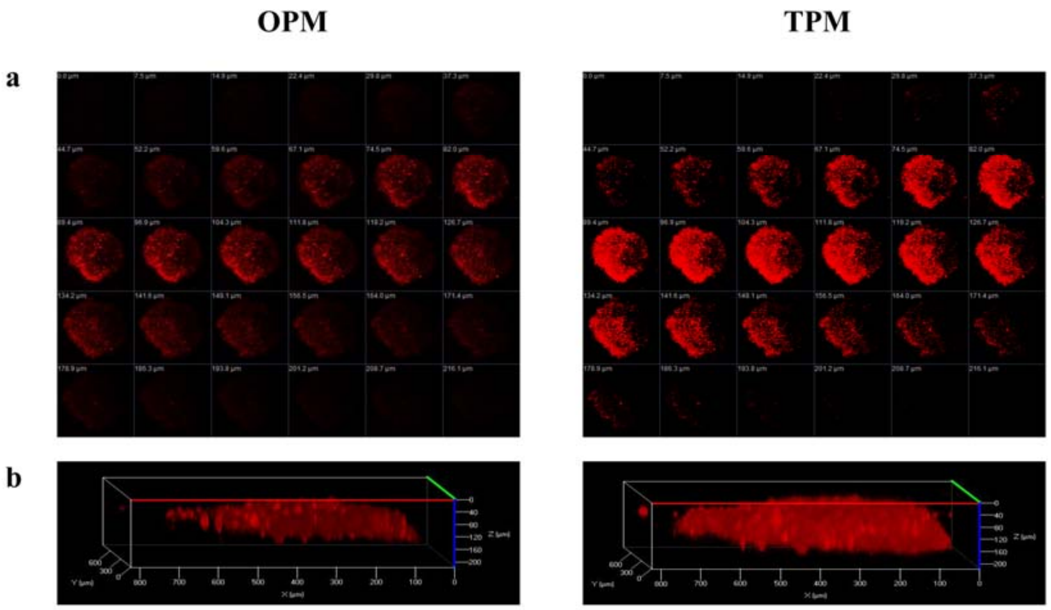

Figure 93. 1- (OPM, λ_{ex} = 458 nm, λ_{em} = 600 – 750 nm) and 2-Photon (TPM, λ_{ex} = 800 nm, λ_{em} = 600 - 750 nm) excited Z-stack images in HeLa MCTS after incubation of **4** after 60 h (20 µM, 2% DMSO, *v*%). **a)** Z-axis images scanning from the top to the bottom of an intact spheroid. **b)** 3D z-stack of an intact spheroid.

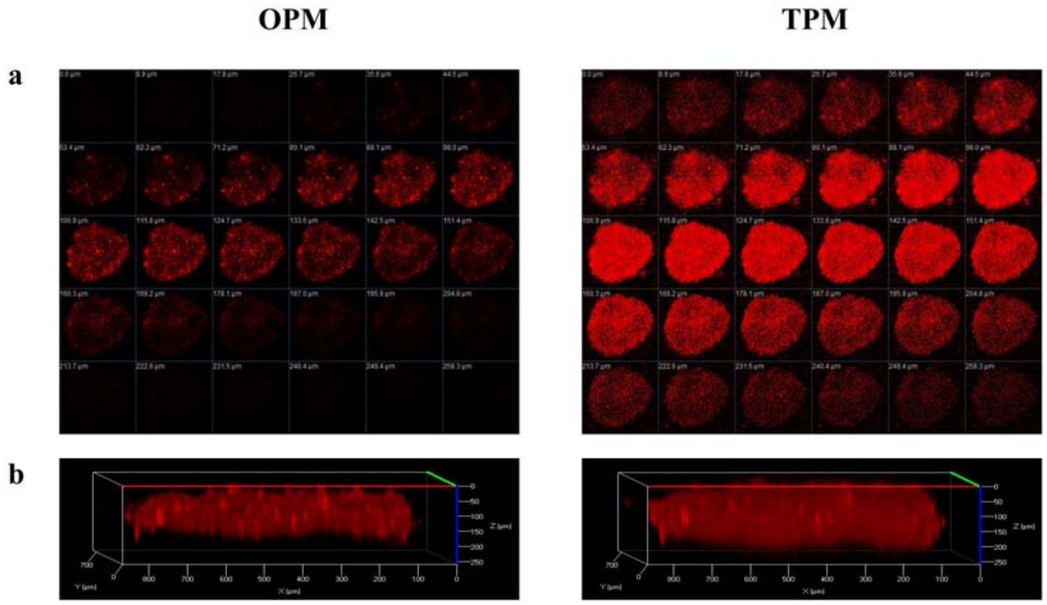

Figure 94. 1- (OPM, λ_{ex} = 458 nm, λ_{em} = 600 – 750 nm) and 2-Photon (TPM, λ_{ex} = 800 nm, λ_{em} = 600 - 750 nm) excited Z-stack images in HeLa MCTS after incubation of **5** after 60 h (20 µM, 2% DMSO, *v*%). **a)** Z-axis images scanning from the top to the bottom of an intact spheroid. **b)** 3D z-stack of an intact spheroid.

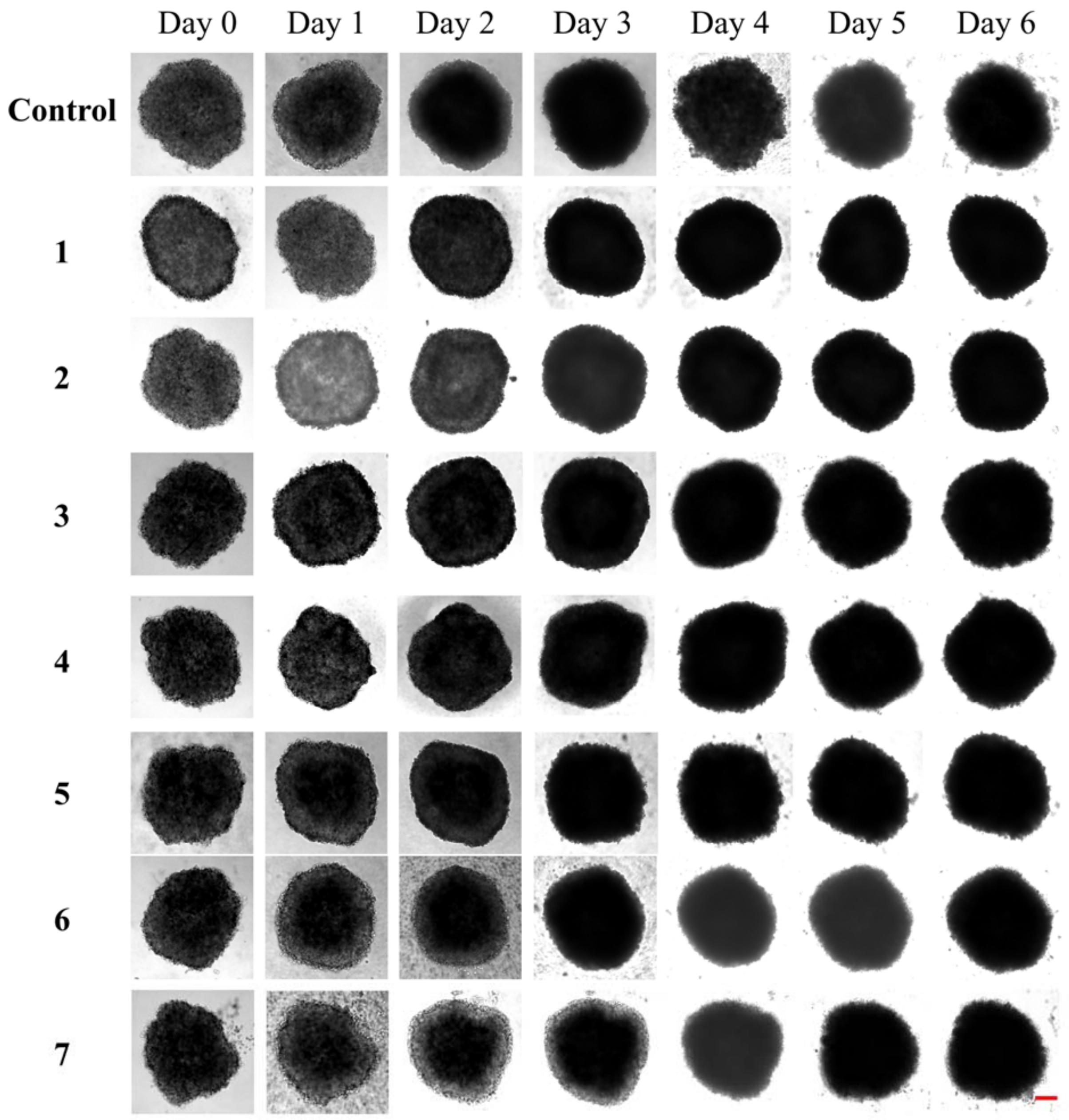

Figure 95. Representative image of the growth inhibition assay in HeLa MCTS. Each MCTS showed to have a diameter of 800 μm on day 0 before the treatment. The MCTS were treated with the compounds **1-7** (20 μM, 2% DMSO, *v*%). MCTS were kept in the dark. The scale bar represents a length of 200 μm.

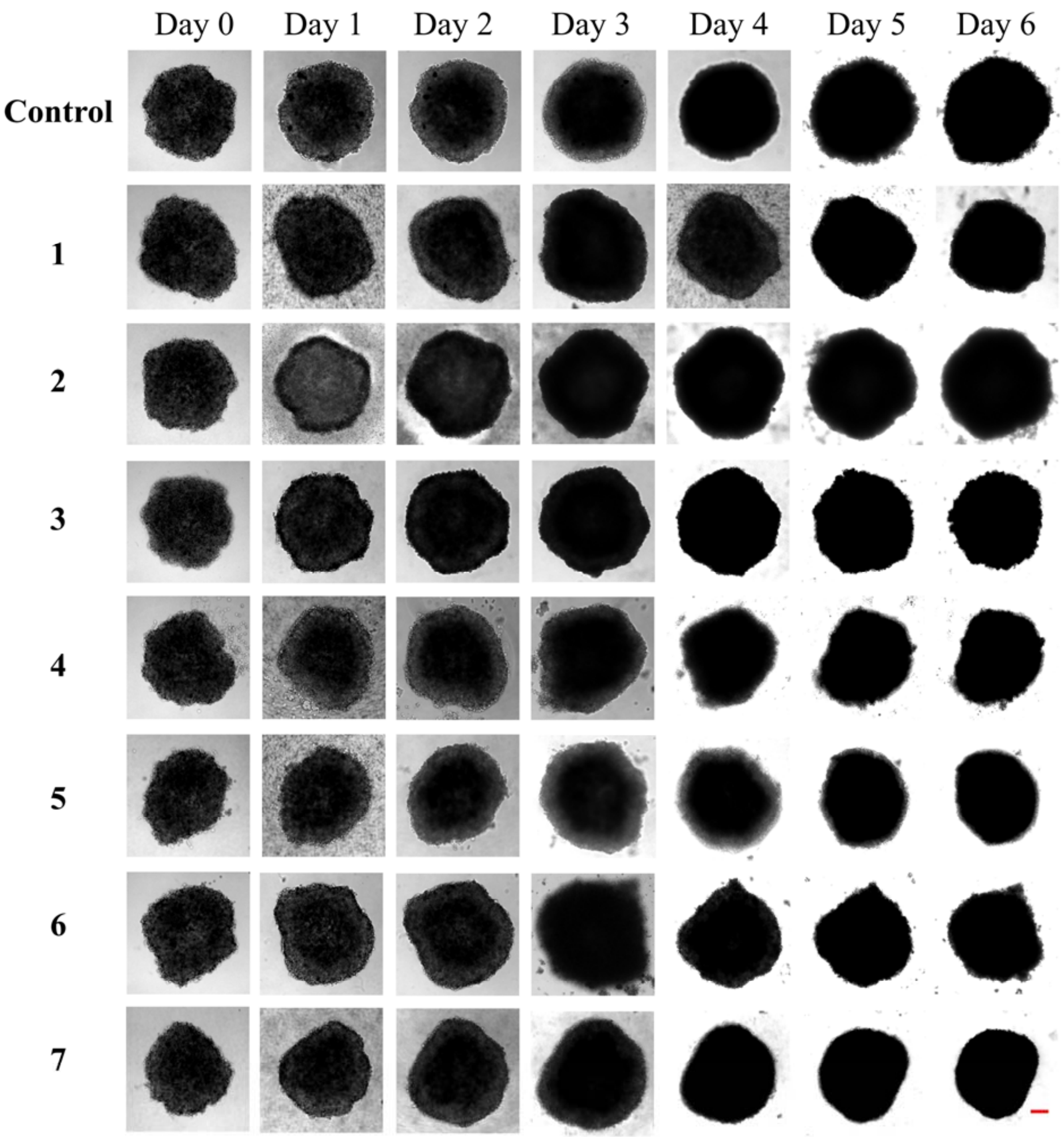

Figure 96. Representative image of the growth inhibition assay in HeLa MCTS. Each MCTS showed to have a diameter of 800 µm on day 0 before the treatment. The MCTS were treated with the compounds **1-7** (20 µM, 2% DMSO, *v*%). The MCTS were exposed to a 1-Photon irradiation (500 nm, 10 J cm^{-2}) on day 3. The scale bar represents a length of 200 µm.

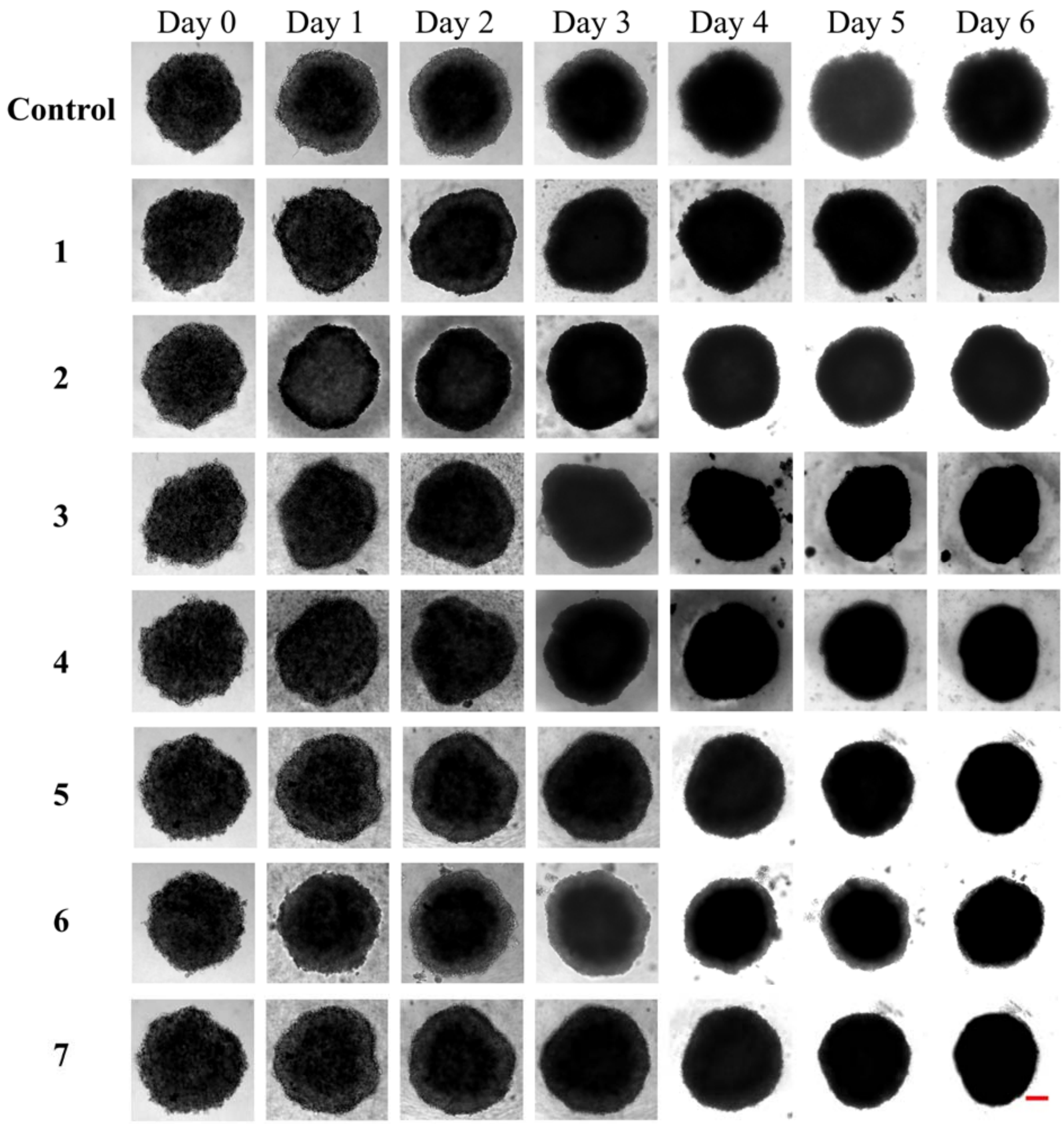

Figure 97. Representative image of the growth inhibition assay in HeLa MCTS. Each MCTS showed to have a diameter of 800 µm on day 0 before the treatment. The MCTS were treated with the compounds **1-7** (20 µM, 2% DMSO, *v*%). The MCTS were exposed to a 2-Photon irradiation (800 nm, 10 J cm^{-2}) with a section interval of 5 µm on day 3. The scale bar represents a length of 200 µm.

Table 13. IC_{50} values in HeLa MCTS in the dark and upon 1P (500 nm, 10 J cm^{-2}) or 2P irradiation (800 nm, 10 J cm^{-2}, section interval of 5 µm) for **1-7** in comparison to cisplatin and tetraphenylporphyrin (H_2TPP). Average of three independent measurements.

	dark	1P irradiation (500 nm, 10 J cm^{-2})	PI	2P irradiation (800 nm, 10 J cm^{-2})	PI
**1**[a)]	>100	>100	n.d.	>100	n.d.
**2**[a)]	>100	>100	n.d.	>100	n.d.
**3**[a)]	>100	>100	n.d.	87.3 ± 6.8	>1.1
**4**	>100	78.3 ± 5.1	>1.3	63.0 ± 4.2	>1.6
**5**	>100	19.3 ± 2.7	>5.2	13.4 ± 3.9	>7.5
**6**	>300	33.8 ± 3.4	>8.9	26.5 ± 2.9	>11.3
**7**	>300	6.8 ± 0.2	>44.1	1.4 ± 0.2	>214.3
**H_2TPP**	>100	>100	n.d.	>100	>100
**cisplatin**	18.6 ± 1.3	-	-	-	-

[a)] due to solubility limitations the compounds were investigated as chloride salts.

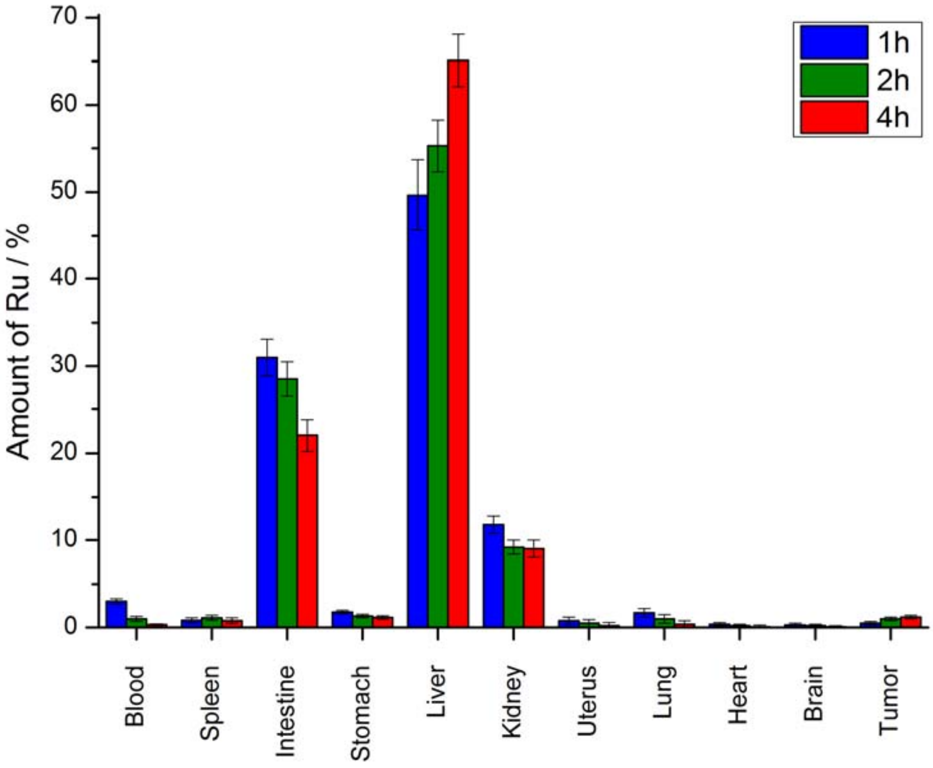

Figure 98. Time dependent biodistribution of **7** in a mouse model with a SW620/AD300 tumour. The error bars correspond to the standard deviation of the three replicates.

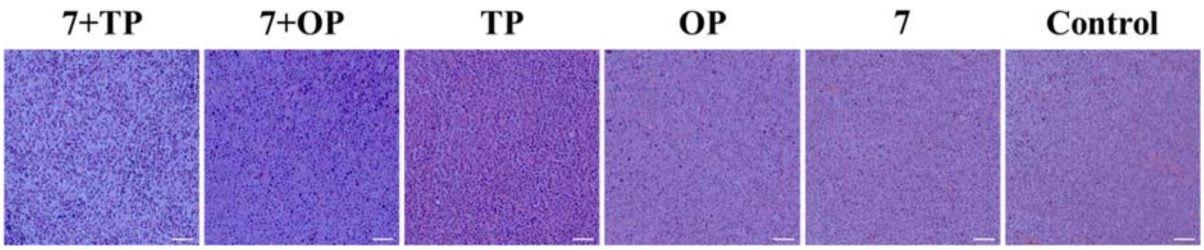

Figure 99. The picture of tumor H&E stain slices after different treatment, from left to right: **7**+TP, **7**+OP, TP, OP, **7** and control. Scale bars: 50 μm.

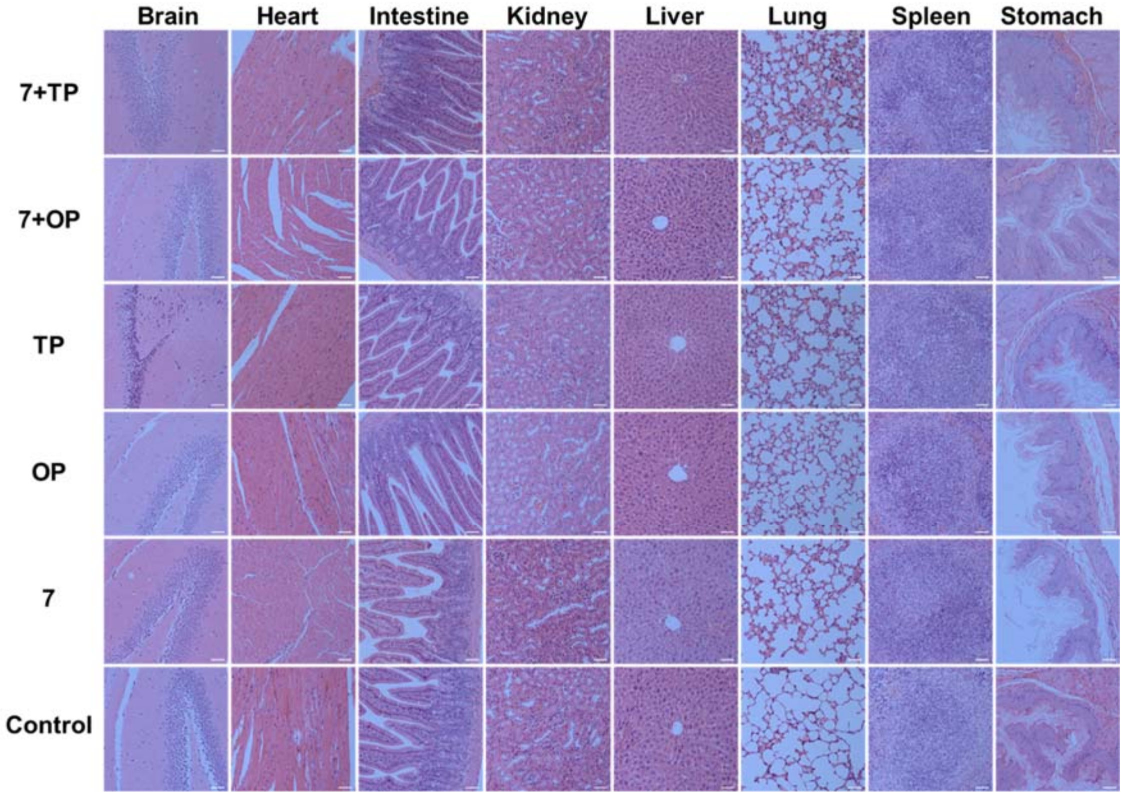

Figure 100. The H&E stained slices for different organs. Scale bars: 50 μm.
